# Consensus Pituitary Atlas, a scalable resource for annotation, novel marker discovery, and analyses in mouse pituitary gland research

**DOI:** 10.1016/j.celrep.2026.117407

**Published:** 2026-06-01

**Authors:** Bence Kövér, Thea L. Willis, Olivia Sherwin, James Kaufman-Cook, Yasmine Kemkem, Miriam Vazquez Segoviano, Emily J. Lodge, Michel Zamojski, Natalia Mendelev, Zidong Zhang, Gregory R. Smith, Daniel J. Bernard, Hui-Chun Lu, Stuart C. Sealfon, Frederique Ruf-Zamojski, Cynthia L. Andoniadou

**Affiliations:** 1 Centre for Craniofacial and Regenerative Biology, King’s College London, London SE1 9RT, UK; 2 Regenerative Medicine Institute, Cedars-Sinai Medical Center, Los Angeles, CA 90048, USA; 3 Department of Neurology, Center for Advanced Research on Diagnostic Assays, Icahn School of Medicine at Mount Sinai (ISMMS), New York, NY, USA; 4 Department of Pharmacology and Therapeutics, McGill University, Montreal, QC H3G 1Y6, Canada; 5 Department of Medicine, Cedars-Sinai Medical Center, Los Angeles, CA 90048, USA; 6 Department of Biomedical Sciences, Cedars-Sinai Medical Center, Los Angeles, CA 90048, USA; 7 Department of Medicine III, Faculty of Medicine Carl Gustav Carus, Technische Universität Dresden, Dresden, Germany; 8 Lead contact

## Abstract

Previous single-cell profiling studies of the pituitary gland have yielded minimally reproducible insights due to their low statistical power and methodological inconsistencies. To address this, we generate the uniformly pre-processed Consensus Pituitary Atlas (CPA) using all 283 existing mouse pituitary single-cell datasets (~1.3 million high-quality cells). The CPA reveals cell typing and lineage markers, including low-expression transcripts that previous analyses could not detect. Leveraging the scale of the CPA, we develop machine learning models to automate and standardize cell type annotation and doublet identification for future studies. Utilizing the curated metadata, we identify sex-biased and age-dependent gene expression patterns at cell type resolution. To uncover drivers of cell fates, first we determine consensus cell communication patterns. Second, we use RNA sequencing and chromatin accessibility data to identify transcription factors associated with cell fates across modalities. The epitome platform provides a user-friendly interface with the CPA and allows streamlined analyses.

## In brief

Single-cell studies of the pituitary gland have been limited by small datasets and inconsistent analyses. Kövér and colleagues integrate 283 datasets (~1.3 million cells) to build the Consensus Pituitary Atlas, which enables robust marker discovery, automated cell annotation with machine learning, and accessible analysis through the epitome platform.

## Graphical abstract

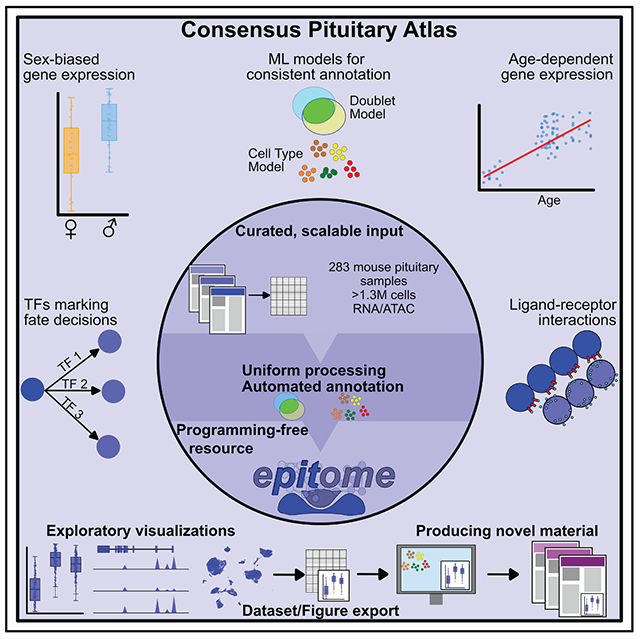

## INTRODUCTION

The pituitary is a key endocrine gland that regulates major hormonal axes across vertebrates and comprises an anterior and a posterior lobe. The anterior pituitary originates from an oral ectoderm invagination termed Rathke’s pouch,^1^ composed of SOX2+ progenitor cells.^2,3^ These progenitors self-renew and give rise to all endocrine pituitary cell types.^4^ Postnatally, SOX2+ cells persist and retain these properties, establishing them as pituitary stem cells.^4^ During differentiation, the stem cells commit to three line-ages, marked by POU1F1 (PIT1),^5^ TBX19 (TPIT),^6^ or NR5A1 (SF1).^7^ POU1F1+ cells give rise to thyroid-stimulating hormone-producing thyrotrophs, prolactin-producing lactotrophs, and growth hormone-producing somatotrophs. The NR5A1+ progenitors differentiate into follicle-stimulating hormone- and luteinizing hormone-producing gonadotrophs. Finally, the TBX19+ lineage gives rise to adrenocorticotropic hormone-producing cortico-trophs and the melanocyte-stimulating hormone-producing mela-notrophs of the intermediate lobe. The posterior pituitary, which is of neural origin, releases the hypothalamic hormones oxytocin and vasopressin/antidiuretic hormone and contains pituicytes. In addition to endocrine and neuroendocrine lineages, the gland includes endothelial, mesenchymal (mostly pericytes postnatally^8,9^), and immune cells.^10^

**Figure 1.**
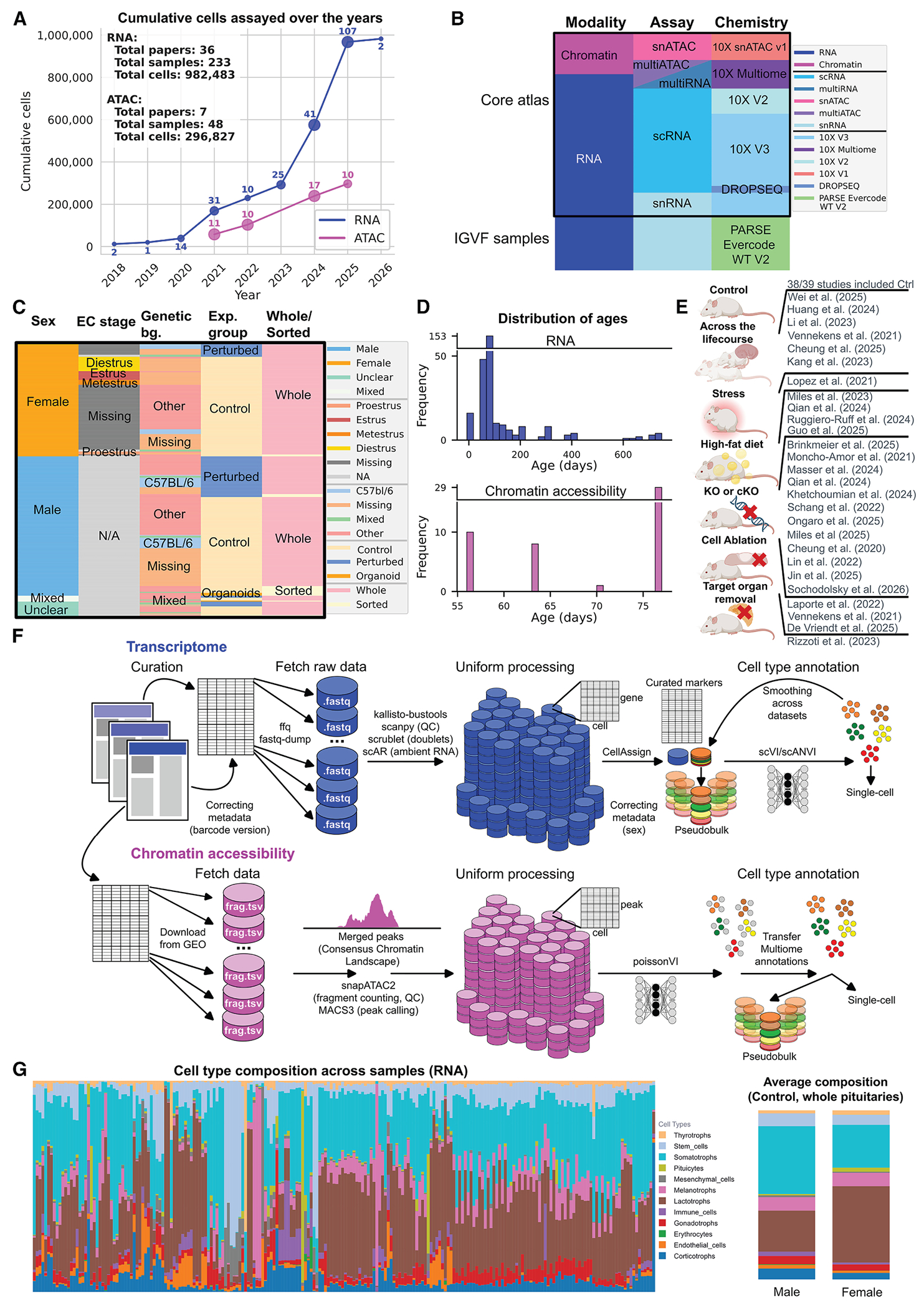
A census of single-cell experiments and generation of the Consensus Pituitary Atlas (A) Cumulative number of cells profiled (RNA in blue and ATAC in pink) up to March 1, 2026. Numbers above dots show the number of datasets; dot size reflects publication count. (B) Metadata summary showing modality, assay type, and chemistry version per sample. (C) Metadata of biological variables: sex, estrous cycle, genetic background, condition (control/perturbed or organoid), and tissue type (whole/sorted). (D) Histogram of sample age distribution (days) for transcriptomic (top) and chromatin accessibility (bottom) samples. Note the broken *y* axes. (E) Schematic of various experimental strategies from the included studies. (F) Schematic of the processing workflow from raw to processed datasets for transcriptomic (top, blue) and chromatin accessibility (bottom, pink) samples. (G) Cell type composition across all RNA samples (left) and mean composition by sex (right).

To characterize pituitary cellular heterogeneity, single-cell and single-nucleus RNA sequencing (sc/snRNA-seq) and assay for transposase-accessible chromatin (ATAC) studies have been performed on 281 samples,^8,10–47^ profiling about 1.3 million cells (Figure 1A). These studies differ in sample preparation, sequencing technologies, read alignment, and downstream analyses, all of which affect outcomes.^48^ Consequently, pituitary single-cell studies report varied numbers of cell types and sub-clusters,^15,34,35,38^ including non-replicable populations such as multihormonal^14^ or “unknown” cells.^41^ Despite abundant data, consensus on pituitary cell type markers remains limited beyond a few canonical examples.

Uniform data processing improves consistency in bulk^49,50^ and scRNA-seq analyses^51,52^ and, when applied across pituitary datasets, can resolve these inconsistencies. To this end, we created the Consensus Pituitary Atlas (CPA), encompassing all publicly available datasets as of March 1, 2026. The CPA enables high-fidelity cell typing and lineage marker identification and reveals cell-type-specific age and sex effects. We further leveraged the CPA to train bespoke models for doublet detection and cell type annotation, validated on a new postnatal day 4 (P4) male multiome dataset. The CPA also identifies transcription factors (TFs) associated with cell fates as well as ligand-receptor interactions, validated by mRNA *in situ* hybridization and immuno-fluorescence. To facilitate access and visualization, we developed *epitome*, a public platform for hypothesis generation, publication-ready figures, and democratized access to uniformly pre-processed pituitary datasets.^53^

## RESULTS

### Generating the CPA

To generate the CPA, all publicly available datasets as of March 1, 2026 were curated, comprising 216 single-cell mouse pituitary datasets (Figures 1B and 1C).^8,10–41,43–47^ The curation process revealed and corrected several metadata errors: 12 of 38 publications or their deposited data contained inaccuracies, including incorrect 10X barcoding kit (12/216 datasets),^18,19,30,32,37^ wrong^19,20,43^ or missing^26,27,32,40,44^ sex information (16/216 datasets), and mixed-up sample metadata (2/216 datasets)^24^ (Table S1). Gene expression datasets spanned a broad age range, while chromatin accessibility datasets were derived exclusively from 8- to 11-week-old animals (Figure 1D). The datasets were generated from control samples (150/216 datasets); genetic, dietary, and physiological perturbations (24 perturbations across 62 datasets); and 4 stem cell organoid samples (Figure 1E).

After uniform pseudoalignment, processing, and quality control (QC; Figure 1F), three datasets (two generated with early DROPSEQ technology^14^) were excluded from further analyses. To the remaining 213 core datasets, we added a recently published set of similarly pseudoaligned datasets from the Impact of Genomic Variation on Function (IGVF) project,^42^ generated using Parse Biosciences’ technology (Figure 1B), contributing an additional 57 datasets after QC. Cell types were annotated using established marker criteria (see STAR Methods).

Uniform processing of chromatin accessibility data^11,21–23,28,36,45^ generated the Consensus Chromatin Landscape (CCL), a set of peaks independently identified in at least 10 datasets. Using CCL-derived peaks increased the average fragment count per dataset to 49.1% of all fragments, compared with 43.4% using dataset-specific peaks (Figure S1A). These CCL peaks do not exhibit study-specific biases (Figure S1B) and capture pituitary cell types similarly, regardless of the cell type abundance bias toward somatotrophs and lactotrophs (Figure S1C). The CCL can complement peak calling in future studies and enable more informative analyses of chromatin accessibility. Cell type annotations were transferred from transcriptomic data to chromatin accessibility datasets using cells assayed in both modalities (multiome assay). Following annotation, all known cell types appeared in their expected proportions across RNA and chromatin modalities (Figures 1G and S1D), also displaying all canonical markers (Figure S1E). In-depth QC analyses of RNA and ATAC processing workflows are displayed in Figures S1, S2, S3, S4, S5, and S6.

Considering both modalities, the atlas comprises 1,278,316 high-quality cells (1,150,001 from the core atlas and 128,315 from IGVF samples), derived from 270 samples out of 281 initially assayed.

### Markers enable doublet detection and scalable cell type annotation

**Figure 2.**
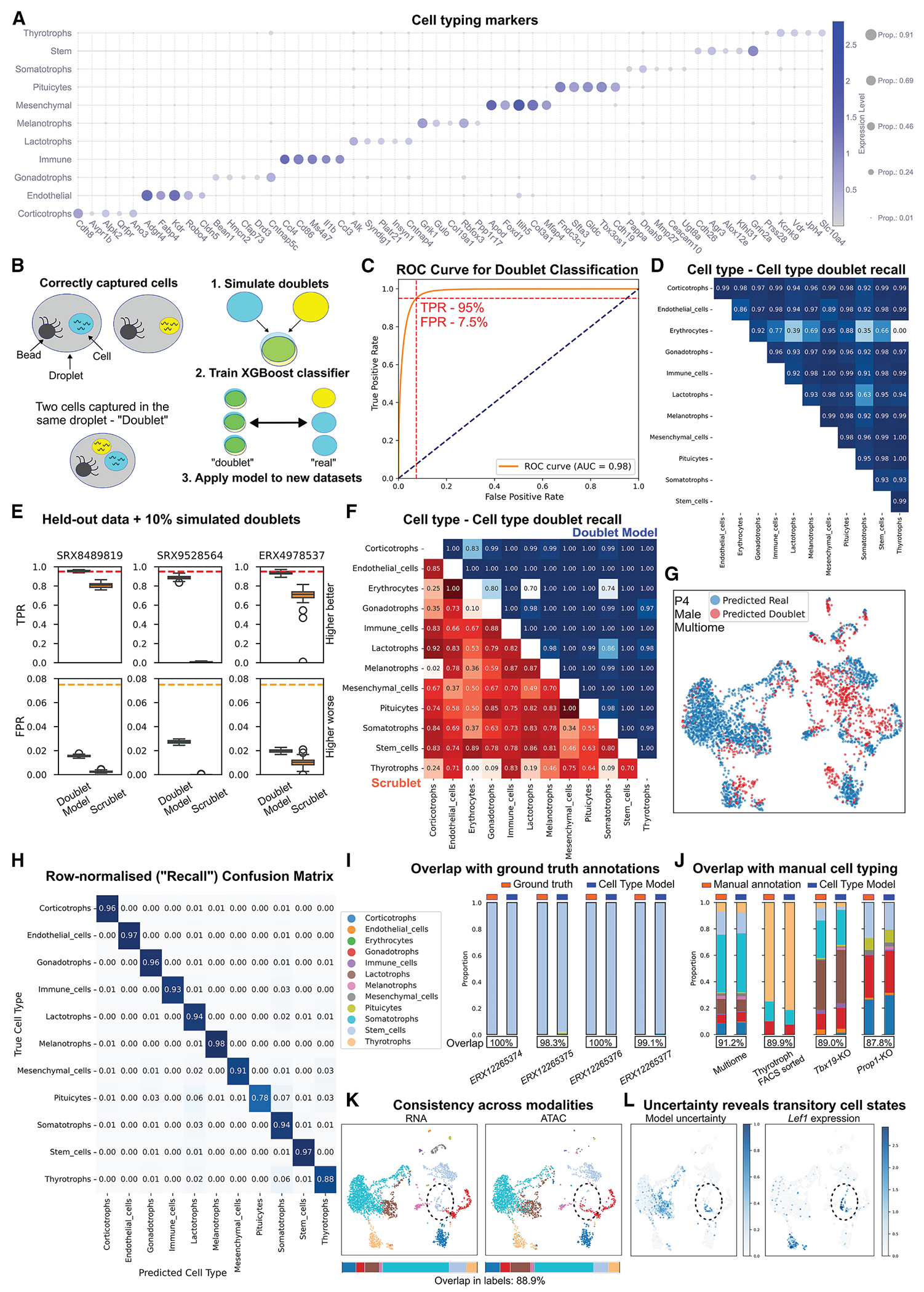
Machine learning models accurately detect simulated doublets and annotate cell types (A) Dot plot of the top 5 cell typing markers from each cell type. (B) (Left) Schematic showing the composition of droplets in droplet-based single-cell RNA sequencing, which might contain single cells (singlets) or two or more cells (doublets). (Right) High-quality filtered and annotated cells were used (1) to simulate doublets and (2) train an XGBoost classifier to separate simulated doublets from real cells, which was then (3) applied to unseen datasets for doublet detection. (C) Receiver operating characteristic curve of the doublet model from the first fold of the 5-fold cross-validation. (D) Recall matrix for simulated doublets of given pairs of cell types. Color intensity of tiles is proportional to the percentage shown. (E) Box plots comparing true and false positive rates (TPR and FPR) of the doublet model and Scrublet across three held-out datasets containing 10% simulated doublets. Red and orange dashed lines indicate calibrated thresholds (95% TPR, 7.5% FPR). (F) Recall matrix for simulated doublets in evaluation datasets, with the Doublet Model (blue) above and Scrublet (orange) below the diagonal. (G) Uniform manifold approximation and projection (UMAP) of the new P4 male multiome dataset showing predicted doublet cells (red) and predicted real cells (blue). (H) Row-normalized confusion matrix comparing cell type model predictions (*x* axis) to true labels (*y* axis), derived from transcriptomic data. (I) Comparison of predicted and ground-truth labels for stem cell organoid samples.^38^ (J) Comparison of predicted and manually assigned labels for the new P4 male multiome dataset and three held-out samples.^11,15,17^ (K) UMAPs of the P4 male multiome dataset colored by RNA- and ATAC-based cell type predictions (top) and corresponding predicted cell type composition (bottom). (L) UMAPs of the same dataset colored by model uncertainty (left) and *Lef1* expression (right).

To address persistent inconsistencies in pituitary cell type labeling, we reasoned that high-quality annotated data could be used to derive robust cell typing markers and train a cell typing model. To expand the repertoire of cell type markers, we identified highly differentially expressed genes (DEGs) by pseudobulking cell profiles, as pseudobulk statistical approaches outperform single-cell-level methods.^54–60^ Canonical markers (e.g., hormone genes) previously used for annotation were excluded, retaining only the freshly identified cell-type-specific genes, shown in Figure 2A (while all markers are found in Table S2). Identified markers were then used to develop doublet detection and cell type annotation models.

Doublets, artifactual pairs of cells captured in the same droplet, complicate cell type annotation (Figure 2B). We hypothesized that a pituitary-specific model would outperform generic approaches. Accordingly, we developed the pituitary-specific “Doublet Model𠆝 (DM) using the XGBoost gradient boosting framework^61^ (Figure 2B; see STAR Methods). Upon 5-fold cross-validation, the DM achieved a mean accuracy of 91.9% (standard deviation (SD): 2.0%) in distinguishing real cells from simulated doublets across studies that were not used in model training. In the first validation fold, the area under the receiver operating characteristic curve was 0.98 (Figure 2C). The prediction threshold was calibrated to yield a 95% true positive rate (TPR) at a 7.5% false positive rate (FPR), i.e., the model correctly identified 95% of doublets, while misclassifying only 7.5% of real cells (Figure 2D).

To benchmark the DM, we compared its performance with the commonly used Scrublet algorithm^62^ applied during the initial workflow (Figure 1F). Three high-quality datasets^11,15,17^ were excluded from training and used for benchmarking. For each, DM and Scrublet were evaluated across 20 simulations containing 10% simulated doublets. The DM consistently outperformed Scrublet (Figure 2E), achieving approximately 95% TPR and only ~2%–3% FPR across all datasets. Scrublet repeatedly failed to detect simulated doublets in one dataset (SRX9528564) and failed to identify many doublet combinations in the remaining two (SRX8489819 and ERX4978537), whereas the DM successfully detected them (Figure 2F). These results highlight the superior accuracy of a tissue-specific model, which can be adapted to other organs.

To demonstrate doublet filtering and subsequent cell typing on new data, we generated a single-nucleus multiome dataset from a P4 male mouse pituitary. The DM predicted 24.9% (921/3,700) of cells as doublets (Figure 2G). These showed higher transcript counts and detected genes than predicted real cells (Figure S7A) and were highly correlated with doublet scores from Scrublet (Figure S7B), overall supporting the validity of the model’s predictions.

We next trained the “Cell Type Model” (CTM) using XGBoost. In a 5-fold cross-validation, the model achieved a mean accuracy of 94.8% (SD: 1.7%) across studies that were not used in model training. All cell types reached high recall (88%–98%; Figure 2H), except pituicytes, which showed a mean recall of 78%. As a first evaluation, the CTM was applied to 4 pituitary stem cell organoid datasets,^38^ showing 98.3%–100% concordance between predicted and ground-truth stem cell annotations (Figure 2I). As a second evaluation, the P4 multiome data (Figure S7C) and three other held-out datasets were tested: one thyrotroph enriched,^24^ one *Tbx19* knockout,^35^ and one *Prop1* knockout.^30^ CTM annotations showed 87.8%–91.2% agreement with manual annotations (STAR Methods; Figure 2J), confirming accurate detection of enriched or absent cell types in the respective datasets. Notably, the CTM identified a subset of immature melanotrophs (*Pomc* negative) in the *Tbx19*-knockout dataset,^35^ which manual annotation had missed (Figure S7D). While the CTM learned the most useful features for cell type annotation (Figure S7E), it also successfully recovered known markers, TFs, and hormones in its results (Figure S7F).

Following this, the CTM was trained on chromatin accessibility data. In a 3-fold cross-validation, the model achieved a mean accuracy of 93% (SD: 0.3%) across studies that were not used in model training (Figure S7G). All cell types showed high recall (84%–97%). Evaluation on the P4 multiome dataset showed 88.9% concordance between chromatin accessibility-based and transcriptomic cell type predictions (Figure 2K). After annotation, the newly generated P4 RNA and ATAC datasets were added to the CPA, bringing the total to 272 datasets and 1,283,270 high-quality cells.

In the multiome dataset, a group of stem cellsin the transcriptomic data was classified as gonadotrophs in the chromatin data (Figure 2K), indicating potentially misaligned annotations. Model uncertainty analysis, however, pointed to a potential transitory state in these cells, which specifically expressed *Lef1* (Figure 2L), a gene recently linked to stem cell commitment toward the gonadotroph lineage.^34^ This supports the view that chromatin changes precede transcriptional activation and highlights model uncertainty as a source of biological insight.

In summary, the CTM accurately identifies cell types and generalizes to unseen data, providing a reproducible framework for cell typing. Both CTM and DM can be run independently or together in a single line of code using the epitome_tools Python package (see STAR Methods).

### Pituitary cell types exhibit sex-biased gene expression downstream of sex hormones

**Figure 3.**
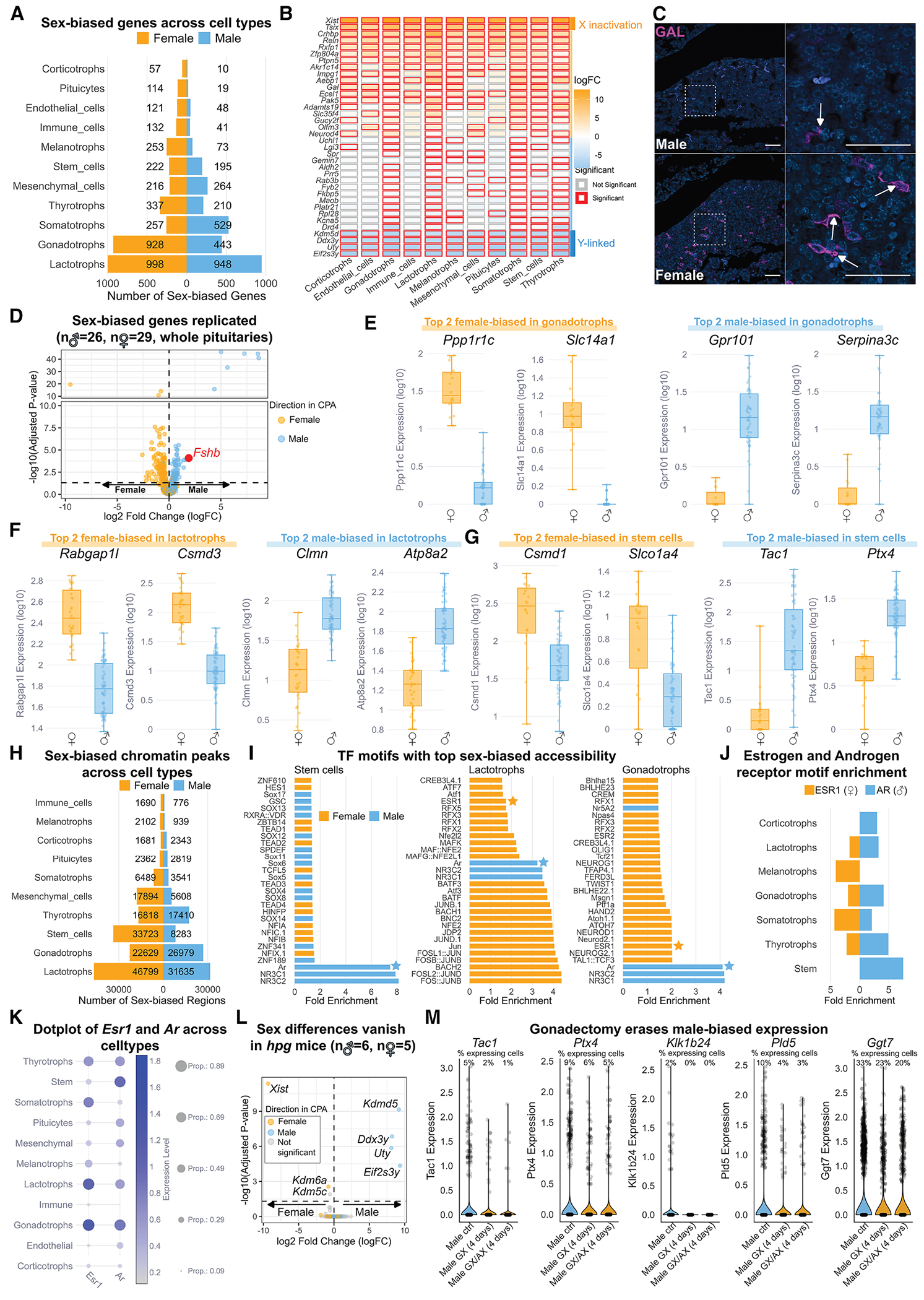
Pituitary cell types exhibit significant sex bias in gene expression, directed by the activity of androgen receptor and estrogen receptor 1 (A) Bar plot showing numbers of statistically significant sex-biased genes (orange: female biased; blue: male biased) across cell types. (B) Heatmap of sex-biased genes (log_2_ fold changes) for top 18 male- and top 18 female-biased genes from those with sex bias in ≥5 cell types. Orange: female biased; blue: male biased. Red rectangles highlight statistically significant cases. (C) Immunofluorescence staining against GAL (magenta) in a male and female pituitary. Scale bars: 50 μm. (D) Volcano plot of sex-biased genes from reanalysis of whole-pituitary male/female bulk RNA-seq samples,^63^ colored according to sex bias in CPA. (E) Box plot of top statistically significant sex-biased genes in gonadotrophs. Left to right: *Ppp1r1c, Slc14a1, Gpr101*, and *Serpina3c*. (F) Box plot of top statistically significant sex-biased genes in lactotrophs. Left to right: *Rabgap1l*, *Csmd3, Clmn,* and *Atp8a2*. (G) Box plot of top statistically significant sex-biased genes in stem cells. Left to right: *Csmd1, Slco1a4, Tac1,* and *Ptx4*. (H) Bar plot of statistically significant sex-biased chromatin accessibility peaks (orange: female biased; blue: male biased) across cell types, ordered by the absolute number of sex-biased peaks. (I) Bar plot of fold enrichment for top sex-biased TF motifs in stem cells, lactotrophs, and gonadotrophs. Orange: female biased; blue: male biased. Stars highlight ESR1 and AR. (J) Bar plot of TF enrichment in sex-biased chromatin accessibility peaks for ESR1 and AR (orange: female biased; blue: male biased). (K) Dot plot of *Esr1* and *Ar* across pituitary cell types. (L) Volcano plot of sex-biased genes from reanalysis of whole pituitary male/female bulk RNA-seq samples from hypogonadal mice^64^, colored according to sex bias in CPA. (M) Violin plots showing *Tac1, Ptx4, Klk1b24, Pld5,* and *Ggt7* expression in male control, GX (gonadectomy), and GX/AX (gonadectomy/adrenalectomy) samples. Percentages indicate fraction of expressing cells.

Previous single-cell profiling studies of the mouse pituitary largely overlooked sex differences,^38^ and the only study addressing them used bulk sequencing, which was confounded by cell type composition, identifying only 75 sex-biased genes.^63^ To fill this knowledge gap, we compared male and female CPA datasets and identified 6,415 instances of sex-biased expression across cell types (Figure 3A). The largest differences were observed in lactotrophs and gonadotrophs (matching findings in rats^9^), with sex bias also present in stem cells (Figure 3A).

We first sought to identify genes with sex-specific expression across multiple cell types. 150 genes showed significant sex bias in ≥5 cell types (top 18 male biased and top 18 female biased shown in Figure 3B). These included known sex-linked genes (female biased: *Xist* and *Tsix*; male biased: *Uty*, *Ddx3y*, *Eif2s3y*, and *Kdmd5*), as well as genes potentially contributing to sex-specific physiological processes, such as *Crhbp*, encoding the corticotropin-releasing hormone-binding protein. Another female-biased gene, termed galanin (*Gal*), encodes a positive regulator of ACTH production in corticotrophs^65,66^ and PRL production in lactotrophs^67,68^ and is stimulated by estrogen.^67,68^ *Gal* levels are also sex biased in rat pituitaries.^9^ Immunostaining for GAL in male and female mice at P30 confirmed the higher abundance of GAL protein in female compared with male pituitaries (5.7% vs. 1.7% of anterior lobe cells; Figure 3C).

To validate CPA findings with an independent approach, we uniformly reprocessed male and female bulk RNA sequencing (RNA-seq) data from whole pituitaries,^63^ gonadotrophs,^69^ and corticotrophs.^70^ Genes significant in both CPA and bulk datasets showed near-complete directional concordance (whole pituitary: 191/197 genes, Figure 3D; gonadotrophs: 232/234 genes, Figure S8A; corticotrophs: 41/42 genes, Figure S8B). We next examined cell-type-specific sex-biased expression, focusing on genes showing bias in no more than three cell types. In gonadotrophs, top hits included *Ppp1r1c*, *Slc14a1*, *Serpina3c*, and *Gpr101* (Figure 3E); the latter is associated with X-linked acrogigantism^71^ and exhibits male-biased expression (Figures S8A and S8C). Additionally, *Fshb* and its regulators *Grem1* and *Gata2* were strongly upregulated in male gonadotrophs, confirming previous findings (Figure S8D).^22^ In lactotrophs, top hits included *Rabgap1l*, *Csmd3*, *Clmn*, and *Atp8a2* (Figure 3F), while stem cell-specific sex-biased genes included *Csmd1*, *Slco1a4*, *Tac1*, and *Ptx4* among others (Figure 3G).

To explore mechanisms underlying sex-biased gene expression, we examined chromatin accessibility patterns. The number of sex-biased peaks across cell types was similar to RNA-level findings, with lactotrophs and gonadotrophs showing the strongest bias (Figure 3H). TF motif analysis revealed strong enrichment of motifs for the androgen receptor (AR) and other nuclear receptors (NR3C1 and NR3C2) in male-specific peaks and for estrogen receptor 1 (ESR1) motifs in female-specific peaks (Figure 3I). This pattern was consistent across most pituitary cell types (Figure 3J). Stem cells lacked a female bias for the ESR1 motif enrichment and showed minimal *Esr1* expression (Figure 3K). At the RNA level, none of the cell types exhibited sex bias in *Ar* or *Esr1* (Table S3), underscoring the added insights from chromatin accessibility analysis.

To validate if sex hormones direct expression differences in the pituitary, we uniformly reprocessed a bulk RNA-seq dataset on hypogonadal (*hpg*) mice,^64^ lacking sex hormone production. In this dataset, significant sex differences vanished (Figure 3L), except those related to sex chromosomes (female biased: *Xist*, *Kdm6a*, and *Kdm5c*; male biased: *Uty*, *Ddx3y*, *Eif2s3y*, and *Kdmd5*). We also retrieved CPA data on stem cells from male controls, males following gonadectomy (GX) mice, and males following both adrenalectomy (AX) and GX.^8^ Male-biased stem cell genes generally (including top 5: *Tac1*, *Ptx4*, *Klk1b24*, *Pld5*, and *Ggt7*; Figure 3M) decreased following GX (Figure S8E), further supporting the role of sex hormones in driving sex-biased expression.

Lastly, we were curious to identify other TFs beyond AR and ESR1 that drive sex-biased gene regulation. As noted earlier, *Gata2* exhibits male-biased expression in gonadotrophs, and one study incorporated in the CPA has assayed mice with *Gata2* conditional knockout (cKO) in gonadotrophs.^22^ Using these datasets, we found 31 genes (including *Fshb*, *Chga*, and *Grem1*) predicted to be activated downstream of GATA2 (levels decrease in *Gata2* cKO) and 19 genes predicted to be repressed downstream of GATA2 (levels increase in *Gata2* cKO) (Figure S8F; Table S4). 7 of these 50 genes were identified as female biased in the atlas and 19 as male biased. Interestingly, 18 out of 19 male-biased genes were GATA2 activated, while 5 out of 7 female-biased genes were GATA2 repressed. The non-random GATA2 regulation of sex-biased genes (*p* = 1.8e–3, Fisher’s exact test; Figure S8G) suggests that GATA2 is a driver of male-biased expression in gonadotrophs.

Overall, these findings highlight AR and ESR1 as central mediators of sex-biased gene regulation in the pituitary and suggest that this regulatory program is in part implemented by an additional layer of TFs, including GATA2 (Figure S8H).

### Pituitary stem cells show an age-dependent inflammatory gene expression program

**Figure 4.**
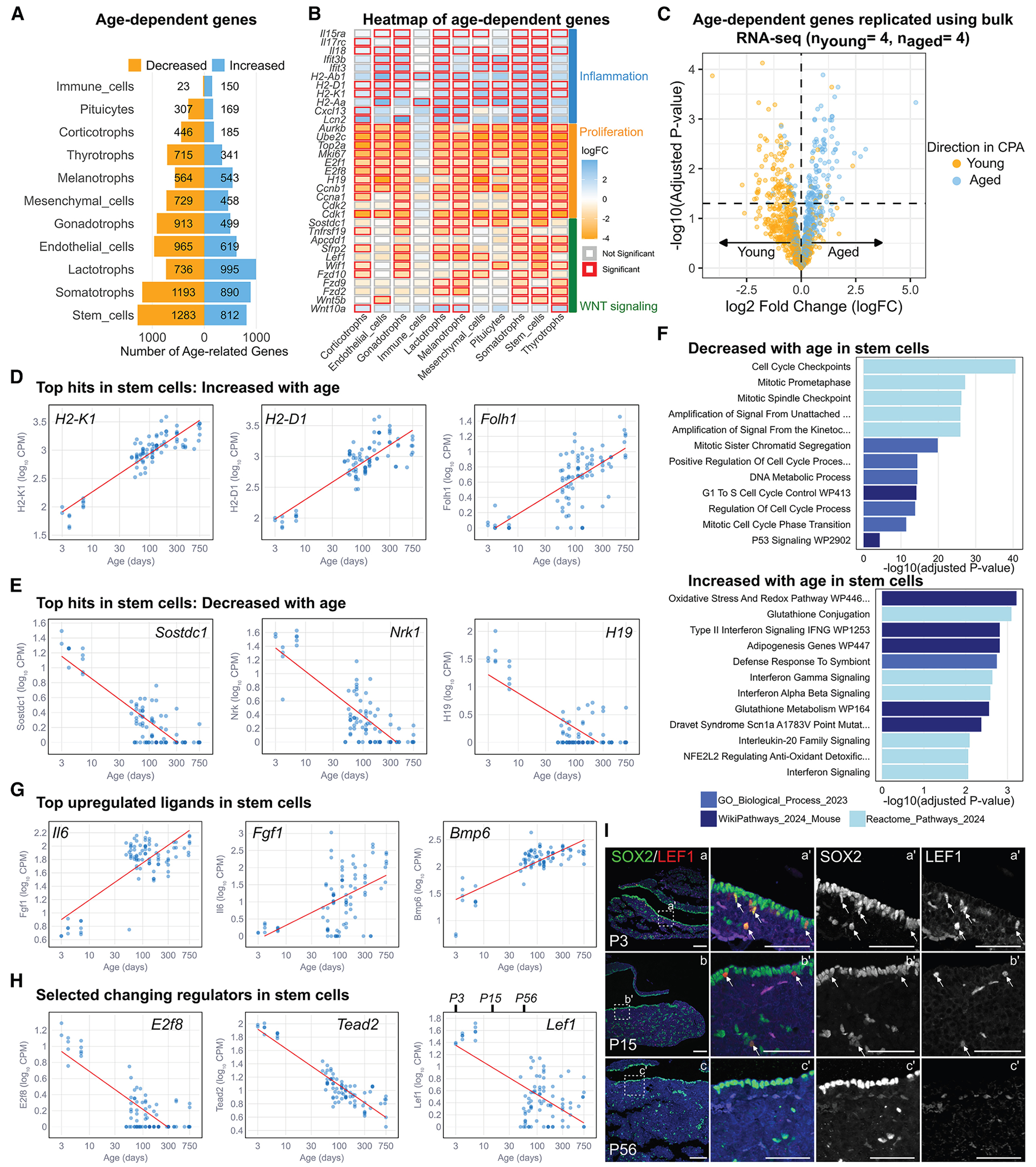
Stem cells exhibit increased inflammation and decreased cell cycle activity with age (A) Bar plot of statistically significant age-dependent genes (orange: higher in young; blue: higher in aged) across cell types. (B) Heatmap of age-dependent genes (log_2_ fold changes) for a curated set of genes that show age dependency in ≥3 cell types. Orange: higher in young, blue: higher in aged. Red rectangles highlight statistically significant cases. (C) Volcano plot from reanalysis of young vs. aged whole-pituitary RNA-seq samples,^19^ colored according to age dependency in CPA. (D) Gene expression versus age (days, spaced out on log_10_ scale) of top 3 statistically significant increasing hits in stem cells. Line of best fit in red. (E) Gene expression versus age (days, spaced out on log_10_ scale) of top 3 statistically significant decreasing hits in stem cells. Line of best fit in red. (F) Bar plot of enriched terms with genes that increase (top) or decrease (bottom) with age in pituitary stem cells. (G) Gene expression versus age (ldays, spaced out on log_10_ scale) of top 3 statistically significant increasing signaling genes in stem cells. Line of best fit in red. (H) Gene expression versus age (days, spaced out on log_10_ scale) of 3 statistically significant selected TF genes in stem cells. Line of best fit in red. Postnatal ages used in (I) are indicated on *Lef1* plot. (I) Immunofluorescence staining against LEF1 (red) and SOX2 (green) in pituitaries of mice at P3, P15, and P56. Dashed squares a′–c′ are magnified. Scale bars: in a–c, 100 μm; in a′–c′ , 50 μm.

We next explored age-related trends for each cell type, revealing 13,535 instances of age-dependent gene expression (Table S5; Figure 4A). DEGs changing in 3 or more cell types with age reveal a pituitary-wide increase in inflammation, decrease in cell cycle activity, and a changing WNT signaling landscape (Figure 4B). To confirm these results, we interrogated a uniformly reprocessed bulk RNA-seq dataset from whole pituitaries of young and aged mice.^19^ There was strong directional agreement between significant genes in both datasets (199/228 showing identical expression trends, Figure 4C).

Stem cells exhibited the highest number of age-dependent DEGs (2,095), nearly 3 times as many as found in a previous study (724 DEGs).^38^ The top changing genes were *H2-K1*, *H2-D1*, and *Folh1*, which increased (Figure 4D), and *Sostdc1*, *Nrk*, and *H19*, which decreased (Figure 4E). Enrichment analysis of stem cell-specific age-dependent genes highlighted terms associated with proliferation/cell cycle activity (decreasing) (Figure 4F) and oxidative stress, inflammatory response, and chemokine/cytokine signaling (increasing). Among increasing hits (Table S5) were genes related to innate immunity (e.g., *Lcn2*, *Ifit3*, and *Ifit3b*), multiple cytokines (e.g., *Il6*, *Il18*, *Cxcl1*, *Cxcl13*, and *Ccl28*), MHC-I/II subunits (e.g., *H2-K1*, *H2-D1*, *H2-Q6*, *H2-Q7*, *H2-Ab1*, and *H2-Aa*), complement-system genes (e.g., *C3* and *C4b*), and interferon-inducible genes (e.g., *ifi47*, *ifi35*, and *ifi203*). Notably, intestinal stem cells also upregulate similar cytokines and MHC-II components with age.^72^ Interestingly, aged pituitaries also had an increased proportion of immune cells (Figure S9A).

The data also revealed an age-dependent shift in the production of paracrine factors. In stem cells, for example, *Fgf16*, *Bmp2*, *Bmp4*, *Wnt5b*, and *Wnt6* showed downregulation, while *Fgf1*, *Fgf2*, *Bmp6*, *Il6*, *Il18*, *Ccl28*, and *C3* were upregulated with age (Figures 4G and S9B). The upregulated secretory profile mirrors a senescence-associated secretory phenotype, associated with certain pituitary tumors,^4,73,74^ with 17 included in the SENMAYO gene signature^75^ (*p-*value: 2.3e–7, Fisher’s exact test). *Cd274*, encoding PD-L1, a well-known immune checkpoint, expressed in pituitary neuroendocrine tumors,^76,77^ was also upregulated with age (Figure S9B).

To understand the drivers of these changes, we queried TFs with age-dependent expression (Figure S9C). Among those decreasing, we found cell cycle regulators *E2f1*, *E2f7*, and *E2f8*, the Hippo pathway effector *Tead2*, and WNT/beta-catenin effector *Lef1* (Figure 4H), all of which may underlie the decreased stem cell contribution with age. Immunofluorescence staining identified LEF1+/SOX2+ cells at P3, and this population decreased by P15 and was undetectable at P56 (Figures 4I, S9D, and S9E). Among increasing TFs, we identified *Nr3c2* (encoding the mineralocorticoid receptor) as well as several others with immune-related functions, such as *Sp100*, *Bhlhe41*, *Stat1*, *Runx1*, and *Tbx21* (encoding T-BET) (Figure S9C).^78–82^ These TFs might drive the upregulation of cytokines and immune-related genes discussed earlier.

Lastly, we aimed to disentangle age-dependent effects into those related to early life changes, adult aging, as well as steady changes over the life course (Figure S10A). We found that in stem cells, most age-dependent changes occur between neonatal and young adult ages (56%; 1,178/2,095), including 72% of downregulated genes (Figure S10B), primarily associated with decreasing cell cycle activity (Figure S10C). From upregulated gene sets, those related to oxidative stress were enriched in the neonatal to young adult transition but not in adult aging. In fact, only 5% of total genes showed a pattern specific to adult aging, and 28% of total genes changed steadily in their expression across the life course. Inflammation-related gene sets were identified among upregulated genes in all three (neonate-to-adult, adult aging, and steadily changing) temporal patterns, suggesting that various aspects of this inflammatory phenotype are triggered at different ages (Figure S10C).

### Consensus lineage markers of stem cells reveal low-expression markers

**Figure 5.**
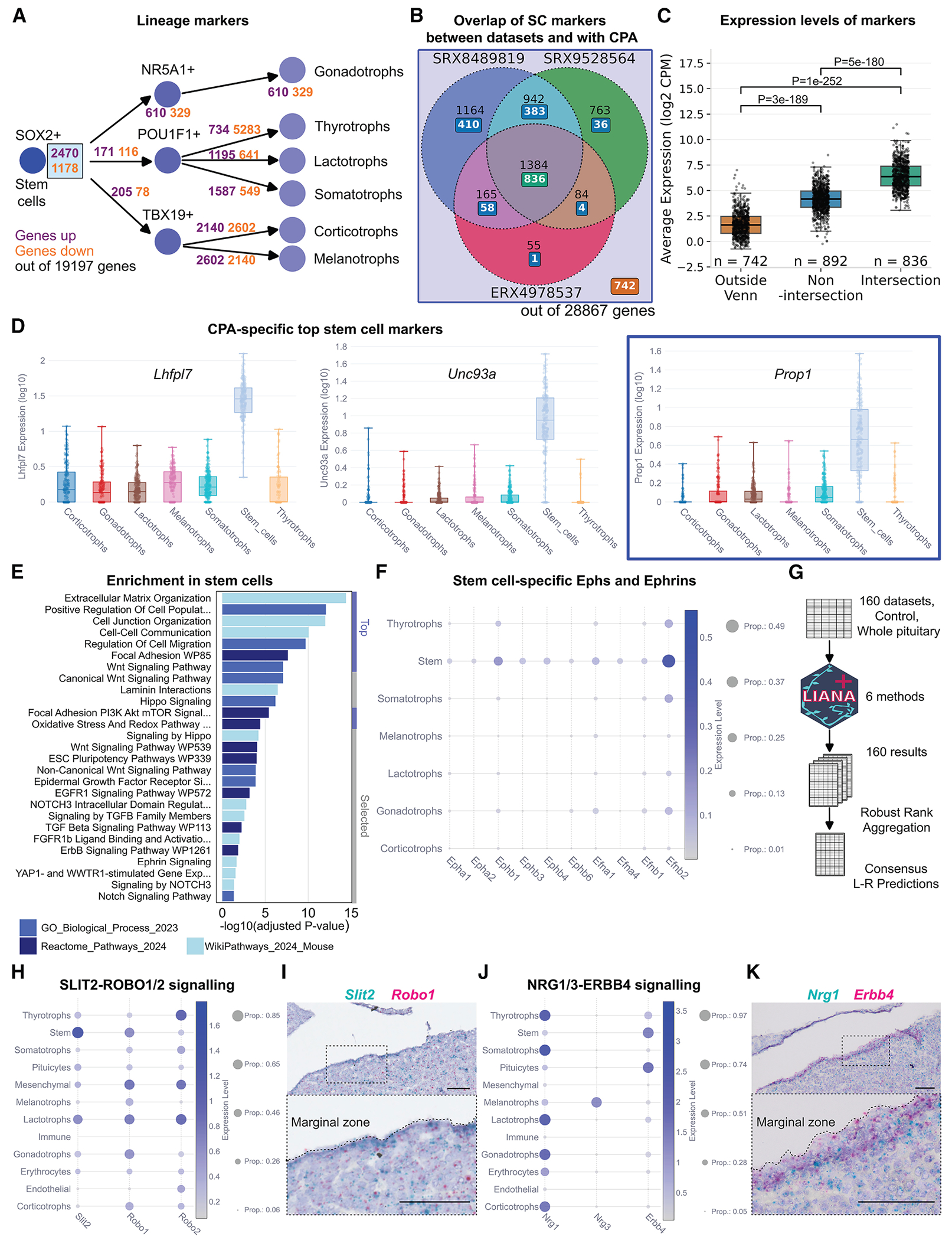
Improved lineage marker detection and transcriptomic analysis using the CPA (A) Schematic listing the number of lineage markers during anterior pituitary differentiation hierarchy (upregulated: purple; downregulated: orange). (B) Venn diagram of stem cell markers from reanalysis of Ruf-Zamojski et al.,^11^ Lopez et al.,^15^ and Vennekens et al.^17^ Boxed numbers indicate overlapping markers derived from the CPA. (C) Box plot of expression values for the three colored groups of CPA markers from (B). P-values derived from Wilcoxon rank-sum test. (D) Box plots of expression values of three low-expression marker genes: (top 2) *Lhfpl7*, *Unc93a* and (selected) *Prop1*. (E) Bar plot of top and selected enriched terms in stem cells using three different databases. (F) Dot plot of Ephs and Ephrins enriched in stem cells. (G) Schematic of ligand-receptor interaction prediction. LIANA+ was run on 160 wild-type datasets, followed by robust rank aggregation for a single consensus set of predictions. (H) Dot plot of *Slit2, Robo1*, and *Robo2* in pituitary cell types. (I) RNAscope mRNA *in situ* hybridization for *Slit2* (red) and *Robo1* (cyan). Scale bars: 50 and 25 μm (inset). (J) Dot plot of *Nrg1*, *Nrg3*, and *Erbb4* in pituitary cell types. (K) RNAscope mRNA *in situ* hybridization for *Nrg1* (cyan) and *Erbb4* (red). Scale bars: 50 and 25 μm (inset).

Differential expression is typically calculated across all cell types present and therefore has limited biological interpretability for specific lineages. To address this, we derived pituitary “lineage markers” by performing differential expression using cell type comparisons corresponding to branchpoints in the pituitary line-age, while accounting for study- and modality-specific effects (Figure S11A; Table S6). Comparing stem cells with all committed cells revealed 2,470 genes associated with stemness and a further 1,178 genes that are lower in stem cells compared with committed cells. Similarly, we carried out these comparisons for all branch-points in the differentiation pathways (Figure 5A).

To determine if these atlas-scale markers provide additional insights compared with interrogating individual datasets, we reanalyzed three high-quality single-cell datasets (Figure 5B).^11,15,17^ There was a great discrepancy between identified stem cell markers, with 1,164, 763, and 55 dataset-specific markers (of all markers, 1,728 overlapped with CPA). 548 out of 1,384 markers consistent across these three datasets (see middle intersection) were not replicable in the CPA (Figures 5B and S11B). We attribute these inconsistencies to the statistical limitations of working with a single dataset, as well as the sample-to-sample variation between animals and labs. Furthermore, the CPA showed a particular advantage over these individual datasets in capturing an additional 742 lowly expressed markers (Figure 5C; top 2 hits were *Lhfpl7* and *Unc93a* for stem cells; Figure 5D) that would not have been identified using either single dataset. Among these low-expression markers was *Prop1* (Figure 5D), a TF with a well-established role in the commitment of pituitary stem cells^83,84^ that has been problematic to detect in single-cell studies.^10^ These results are in line with pseudobulk analysis being more sensitive to low-expression genes.^57^ We then also identified additional markers (including low-expression ones) compared with individual datasets, for all other points in the pituitary lineage (Figure S11C; Table S7). Strikingly, from the 2,602 upregulated genes in corticotrophs compared with melanotrophs in the CPA, only 134 would have been uncovered by the intersection of the 3 individual datasets (Figure S11C). Overall, these results demonstrate the power of using the CPA for marker discovery compared with individual datasets.

We then performed gene set enrichment on stem cell lineage markers, which revealed known pituitary stem cell pathways (Table S8), such as WNT signaling,^85^ epidermal growth factor (EGF) signaling, fibroblast growth factor (FGF) signaling, Notch signaling,^86,87^ and the Hippo pathway^1,88,89^ (Figure 5E). Additional enriched terms included Eph-ephrin signaling and laminin interactions (Figure 5E). Several low-expression ephrins (*Efna1*, *Efna4*, *Efnb1*, and *Efnb2*) and Eph receptors (*Epha1*, *Epha2*, *Ephb1*, *Ephb3*, *Ephb4*, and *Ephb6*) are specific to stem cells (Figure 5F), while other Ephs and Ephrins are expressed across pituitary cell types (Figure S11D). This significantly extends previous work that has already identified EFNB2 in rat pituitary stem cells.^90^ The laminin genes enriched in stem cells included *Lama3*, *Lama5*, *Lamb2*, *Lamb3*, *Lamc1*, and *Lamc2* (Figure S11E).

### Consensus ligand-receptor interactions reveal paracrine signaling between stem and endocrine cells

Since stem cell lineage markers were enriched for cell-cell communication genes (Figure 5E), we next investigated ligand-receptor interactions across all control, whole-pituitary sc/snRNA-seq datasets (160 datasets, 682,983 cells). We applied LIANA+,^91^ which integrates multiple ligand-receptor inference algorithms, and merged results using robust rank aggregation^92^ (STAR Methods; Figure 5G). Cell-type-specific interactions are summarized in Table S9.

Stem cells showed high *Slit2* expression encoding SLIT2, predicted to signal through ROBO1 and ROBO2 on differentiated cell types (Figure 5H). RNAscope mRNA *in situ* hybridization confirmed this pattern showing *Slit2* localization near the marginal zone (stem cell niche) and *Robo1* in adjacent cells (Figure 5I). Additional predicted stem cell interactions included the ERBB4 receptor engaging with NRG3 from melanotrophs and NRG1 from other differentiated cell types (Figure 5J). RNAscope mRNA *in situ* hybridization confirmed *Erbb4* expression near the marginal zone and *Nrg1* in adjacent cells (Figure 5K). Interestingly, stem cells were also predicted to be the sole source of BMP6, while all other cell types variably expressed its receptors (*Bmpr1a*, *Bmpr2a*, *Acvr1*, and *Acvr2a*) (Figure S11F). In addition, stem cells emerged as the main source of *Fgf1* expression along with corticotrophs. Receptor genes *Fgfr1* and *Fgfr2* were expressed primarily by stem cells and broadly across other cell types, respectively (Figure S11G). Finally, this approach also highlighted TGFB2-TGFBR1/2/3 interactions, with *Tgfb2* expression restricted to stem cells and receptor expression detected across all cell types (Figure S11H).

These results revealed several highly specific interactions between all cell types, including many between pituitary stem cells and endocrine cells, reinforcing the notion of pituitary stem cells as paracrine signaling hubs.^93,94^

### Differential expression analysis for perturbations: High-fat diet as a case study

The CPA presents a unique opportunity to compare effects of perturbations across studies as all datasets are uniformly processed. To date, the only perturbation assayed across multiple publications has been the high-fat diet (HFD).^16,20,31,46^ A naive approach of considering the intersections of DEGs across HFD studies revealed remarkably little overlap, possibly explained by differences in background strain and age of samples used in these studies (Figure S11I). We then performed linear mixed modeling to jointly analyze HFD studies, which identified a minimal set of changing genes mostly in stem cells and immune cells (Figure S11J; Table S10) but not in endocrine cells. Interestingly, immune cells upregulated *Cd8a* and *Cd8b1*, suggesting an increase in CD8^+^ cytotoxic T cells following HFD (Figure S11K). An increase in CD8^+^ T cells has been observed in the brains of mice following HFD^95^ and in the hypothalami of obese patients.^96^ Overall, this example highlights the power of jointly analyzing uniformly processed datasets from multiple studies, as well as the minimal reproducibility across experiments in the literature.

### Candidate TFs associated with cell fates across modalities

**Figure 6.**
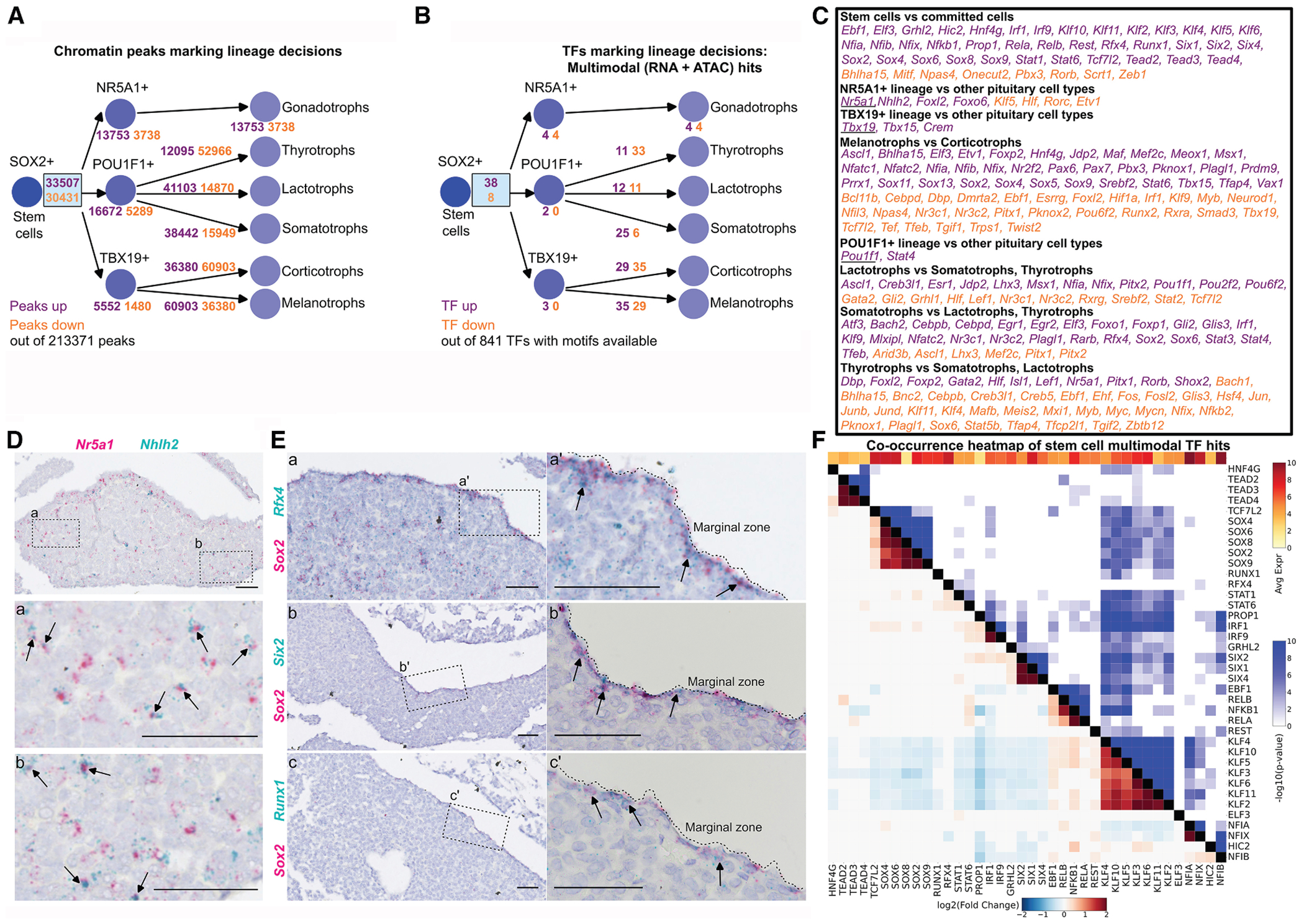
Identification of fate-specific transcription factors through transcriptomics and chromatin accessibility (A) Schematic of the number of lineage marker peaks in the differentiation hierarchy of the pituitary lineage (opening: purple; closing: orange). (B) Schematic of the number of multimodal hit TFs in the differentiation hierarchy of the pituitary lineage (upregulated: purple; downregulated: orange). (C) Table of all multimodal hits (upregulated: purple; downregulated: orange). (D) RNAscope mRNA *in situ* hybridization for *Nr5a1* (red) and *Nhlh2* (cyan). Scale bars: 50 μm. (E) RNAscope mRNA *in situ* hybridization for target TFs. Top: *Sox2* (red) and *Rfx4* (cyan); middle: *Sox2* (red) and *Six2* (cyan); bottom: *Sox2* (red) and *Runx1* (cyan). Scale bars: 50 μm. (F) Motif co-occurrence heatmap of multimodal hit TFs in stem cells. Rows and columns share the same TF order. Colors below the diagonal represent fold change of observed versus expected co-occurrence rates, while colors above indicate statistical significance (−log_10_ *p*-value). P-values derived from Fisher’s exact test.

Building on gene expression analyses, we examined chromatin accessibility changes along the pituitary lineage, while accounting for study- and modality-specific effects (Figures S12A and S12B; Table S11). This identified 33,507 peaks more accessible in stem cells and 30,431 peaks opening in all committed lineages (Figure 6A). Multimodal hits were then defined at each cell fate decision point (Figures 6B and 6C; Table S12), based on TFs that were both differentially expressed and whose motifs were enriched in differentially accessible peaks. Because this approach depends on known binding motifs and also excludes TFs whose broad accessibility remains unchanged, we also generated an “RNA-only” list (Figures S12B and S12C; Table S12).

The TBX19+ lineage contained 3 multimodal hits: *Tbx19*, *Tbx15*, and *Crem* (Figures 6B and 6C). RNA-only hits included *Esrrb*, *Zim3*, *Hopx*, *Scx*, *Zfp474*, *Egr4*, *Nr4a2*, and *Prdm1* (Figure S12C; Table S12). Within this lineage, several multimodal hits differed between corticotrophs and melanotrophs. Among these, the melanotroph TF-encoding gene *Pax7* is well established as a determinant of melanotroph over corticotroph fate,^97^ while *Etv1* encodes a key transcriptional regulator active in this lineage.^98^ Corticotroph-specific TFs included *Neurod1* and the glucocorticoid receptor *Nr3c1*. *Neurod1* is required for proper corticotroph differentiation,^99^ regulates *Pomc* transcription,^100,101^ and is expressed in corticotroph adenomas.^102^

In the NR5A1+ lineage, we identified *Nhlh2*, *Foxl2*, and *Foxo6* as additional multimodal hits (Figures 6B and 6C). To confirm the specificity of *Nhlh2* in gonadotrophs, we performed double RNAscope mRNA *in situ* hybridization for *Nhlh2* and *Nr5a1*, which showed clear co-expression (Figure 6D). In gonadotrophs, *Nhlh2* displayed female-biased expression (Figure S12D), of relevance to future functional studies. RNA-only hits included *Aff2*, *Aff3*, *Foxp2*, *Glis1*, *Hes6*, *Irf8*, *Lhx4*, *Nr0b1*, *Nr0b2*, *Pbx4*, *Pgr*, *Sox13*, *Sox5*, *Sp140*, *St18*, *Tcf24*, *Tcf7*, *Zfp41*, *Zfp579*, *Zfp661*, *Zfp820*, *Zfp872*, and *Zfpm1* (Figure S12C; Table S12).

In addition to *Pou1f1*, the POU1F1+ lineage contained *Stat4* as an additional multimodal hit (Figures 6B and 6C). RNA-only hits included *Vdr*, *Rxrg*, *Shox2*, and *Klf14* (Figure S12C; Table S12). *Vdr* encodes the vitamin D receptor, whose homozygous mutants show reduced growth and IGF-1 levels.^103,104^ *Rxrg* encodes retinoid X receptor gamma, which is involved in growth regulation and thyrotroph function.^29,105,106^ RXRG and VDR can act cooperatively and have previously been implicated in *Pou1f1* regulation.^107–109^

Within the POU1F1+ lineage, we also resolved TF-encoding genes that might drive differentiated cell type identities. Somatotroph-specific multimodal hits included genes previously implicated in somatotroph differentiation *Gli2*,^110,111^ *Foxo1*,^112,113^ and *Nr3c1.*^114,115^ Thyrotroph-specific multimodal hits interestingly included *Nr5a1*, despite its canonical role in gonadotroph fate, as well as various other previously identified genes, such as *Gata2*, *Isl1*, *Foxl2*, and *Shox2*.^24^ Lactotroph multimodal hits included *Esr1* encoding the estrogen receptor, as well as *Pou6f2*. Interestingly, *POU6F2* mutations have been identified in prolactinomas.^116^

Multimodal hits associated with stem cells included genes from the SOX (*Sox2*, *Sox4*, *Sox6*, *Sox8*, and *Sox9*) and TEAD (*Tead2*, *Tead3*, and *Tead4*) families, both known regulators of pituitary stem cells.^1,4,89^ Additional TF families included KLF (*Klf2*, *Klf3*, *Klf4*, *Klf5*, *Klf6*, *Klf10*, and *Klf11*), NFI (*Nfia*, *Nfib*, and *Nfix*), SIX (*Six1*, *Six2*, and *Six4*), IRF (*Irf1* and *Irf9*), STAT (*Stat1* and *Stat6*), and REL (*Rela* and *Relb*), along with single TF-encoding genes (*Ebf1*, *Elf3*, *Grhl2*, *Hic2*, *Hnf4g*, *Nfkb1*, *Prop1*, *Rest*, *Rfx4*, *Runx1*, and *Tcf7l2*). Members of the TEAD, NFI, and SIX families showed the strongest enrichment in stem cell-associated chromatin regions (Figure S12E). Double RNAscope mRNA *in situ* hybridization confirmed *Runx1*, *Rfx4*, and *Six2* expression alongside *Sox2* in pituitary stem cells (Figure 6E).

We then examined motif co-occurrence across stem cell-specific multimodal hits (Figure 6F). Surprisingly, KLF binding sites were significantly less likely than expected to co-occur with other TF motifs within the same peaks (Figure 6F). Genomic annotation of KLF motifs showed that they were enriched in proximal regulatory regions relative to other TF motifs that are more abundant in distal intergenic regions (Figures S12F and S12G), suggesting a functional basis for their limited overlap.

### The epitome: A programming-free interface for the CPA

Democratized access to the CPA is key to advancing reproducibility and collaboration. To this end, we developed the electronic pituitary omics (epitome) platform,^53^ which currently hosts the CPA at epitome-atlas.com and will be continuously updated with published datasets. Through epitome, users can perform extensive exploratory analyses online across published transcriptomic and chromatin accessibility datasets. Available secondary analyses include LIANA+ ligand-receptor interactions,^91^ gene-gene coexpression, age-related expression trends, TF binding motif visualization, TF binding motif enrichment via ChromVAR,^117^ and generating TF motif co-occurrence heat-maps. The platform generates publication-ready figures for all these analyses, facilitating seamless integration into research workflows. Uniformly processed datasets are also available for download, enabling local analysis. Lastly, *epitome* enables automated processing and annotation (through CTM and DM) of users’ own data, all without programming or formal CPA data submission.

## DISCUSSION

Fulfilling their promise of cell-type-specific insights, single-cell genomic methodologies are truly effective when carried out at a grand scale. Here, we present the CPA, a comprehensive and scalable resource, encompassing all currently available mouse pituitary single-cell datasets. The CPA enables statistically robust analyses and serves as a definitive reference for this organ. As a proof of concept, we use the CPA to reveal sex- and age-related effects on gene regulatory programs at unprecedented resolution, underscoring the power of a domain-specific atlas that can be readily extended to other tissues and species.

The CPA also enables more accurate marker identification than individual studies, yielding numerous robust cell-type-specific TF candidates, including previously undescribed regulators. For example, *Rfx4* emerges as a stem cell TF, showing stem cell-specific expression and chromatin accessibility. Pathogenic *RFX4* variants have been reported in six patients with neurodevelopmental disorders, two of whom also presented pituitary anomalies including agenesis,^118^ supporting its functional relevance. Cell-cell communication analyses identified SLIT-ROBO signaling among top pathways. Multiple hypopituitarism patients carry *ROBO1* variants,^119–123^ previously linked to defects in the neural pituitary.^120^ Our finding that stem cells and lactotrophs may secrete SLIT2, signaling via ROBO1/2 receptors across endocrine cell types, offers insights into anterior pituitary phenotypes. Thus, a CPA framework can guide prioritization for future pre-clinical functional studies.

Analysis of multimodal hits at lineage decision points successfully recovered the three key TFs of commitment (NR5A1, POU1F1, and TBX19) and revealed additional candidates whose roles remain unexplored in pituitary differentiation. For instance, *Nhlh2* was an additional TF gene identified alongside *Nr5a1* in gonadotrophs. Although *Nhlh2*^−/−^ knockout mice retain a gonadotroph population, they exhibit hypogonadism,^124^ so far linked to hypothalamic kisspeptin neurons,^125^ and also display pituitary hypoplasia and downregulated *Gnrhr* expression in gonadotrophs,^126^ suggesting a direct role in pituitary development.

Future studies will be required to follow up on the TF hits discussed earlier. Previous benchmarking showed that computational approaches are unable to reliably predict individual TF-gene regulatory interactions.^127^ By contrast, sequence-to-function models,^128^ which link DNA sequence features directly to regulatory activity, hold promise for deciphering TF function. The scale of the CPA will enable such analyses to further characterize the identified TFs (e.g., *Rfx4* or *Nhlh2*) along with necessary functional studies.

The CPA also provides a framework to study gene expression changes across the life course and sexual dimorphism. For example, *Crhbp*, encoding corticotropin-releasing hormone-binding protein, showed strong female bias across all cell types, being nearly absent in males. As CRHBP sequesters corticotropin-releasing hormone, a key hypothalamic regulator of ACTH, this difference may underlie sex-biased stress responses. Indeed, *Crhbp*^−/−^ knockout phenotypes exhibit sex-specific gene expression and stress-related behavioral patterns.^129,130^ The CPA also uncovered an age-associated inflammatory program within pituitary stem cells, revealing coordinated transcriptional and paracrine shifts reminiscent of tissue senescence. Aged stem cells showed marked upregulation of cytokines, interferon-responsive genes, and complement genes, together with immune checkpoint molecules such as *Cd274* (PD-L1). On the other hand, proliferative and stem cell-related factors, including *E2f1*, *Tead2*, and *Lef1*, declined with age. This dual profile — heightened immune-related paracrine signaling and reduced regenerative potential — mirrors a senescence-associated secretory phenotype,^75^ and may contribute to functional decline in the pituitary with age. The age-dependent upregulation of immune-related TFs (*Bhlhe41*, *Sp100*, *Stat1*, *Runx1*, and *Tbx21*) further points to a transcriptional reprogramming of stem cells toward an inflammatory state, similar to those reported in intestinal stem cells^72^ and in the aging brain.^131^ BHLHE41 potentially represses the E2F network that participates in proliferation,^82^ whereas STAT1 has been previously shown to drive inflammaging in intestinal stem cells,^72^ upregulating similar genes to those observed here. Together, these findings suggest that age-dependent changes in the pituitary involve an intrinsic, stem cell-centered inflammatory remodeling, which could influence susceptibility to endocrine insufficiency or tumorigenesis later in life.

Finally, the CPA is complemented by the epitome platform,^53^ a programming-free, user-friendly interface for exploring and analyzing the atlas, in line with the FAIR (Findable, Accessible, Interoperable, Reusable) guiding principles.^132^ Epitome allows users to interrogate all existing mouse datasets or to analyze their own data without prior submission. The accompanying epitome_tools Python package further enables rapid, expert-level cell type annotation and doublet filtering via machine learning, using a single command. Together, these resources democratize data access, promote standardized best practices, and accelerate discovery without requiring programming expertise. Looking forward, both the atlas and platform can expand to additional modalities, such as spatial transcriptomics, single-nucleus methylomics, and single-cell proteomics.

### Limitations of the study

This work highlights TF-encoding genes associated with cell fates based on gene expression and chromatin accessibility. However, even strong sequence enrichment in chromatin peaks does not prove TF binding, and future work will require functional assays.

In addition, across our analyses, results for POU1F1+, TBX19+, and NR5A1+ intermediate progenitors are inferred; however, these populations are transient and may not be sufficiently captured by single-cell approaches in postnatal samples. Future datasets could enrich for these transient cells by various cell sorting strategies to directly resolve their intermediate transcriptomic profiles.

Since datasets were collated from studies with various experimental designs, residual confounding between covariates cannot be fully excluded. For example, in the analysis of age-dependent gene expression patterns, constituent datasets were not uniformly distributed across age groups, with some studies only contributing to specific age ranges.

## RESOURCE AVAILABILITY

### Lead contact

Further information and requests for resources and reagents should be directed to and will be fulfilled by the lead contact, Prof. Cynthia L. Andoniadou (cynthia.andoniadou@kcl.ac.uk).

### Materials availability

This study did not generate new unique reagents or organisms.

## STAR★METHODS

### EXPERIMENTAL MODEL AND STUDY PARTICIPANT DETAILS

Animal husbandry was carried out under compliance of the Animals (Scientific Procedures) Act 1986, Home Office license and KCL ethical review approval. Pituitaries from CD-1 mice were collected from male and randomly-cycling female mice. Animals were on a 12-h on, 12-h off light cycle (lights on at 7 a.m.; off at 7 p.m.).

A male mouse with mixed CD-1 and C57BL6/J background (backcrossed on CD-1 for 5 generations) was collected for multiome data generation at postnatal day 4.

A male and female mouse with CD-1 backgrounds aged P30 were used for immunofluorescence staining against GAL, whereas mice with mixed background and ages P3, P15 and P56 were used for SOX2/LEF1 stainings.

Female mice with CD-1 backgrounds aged P21 were used for RNAscope *in situ* hybridization targeting *Nrg1*-*Erbb4*, *Slit2*-*Robo1*, *Nr5a1*-*Nhlh2*, *Rfx4-Sox2* and mice aged P56 for *Runx1-Sox2* and *Six2-Sox2.*

Based on transcriptomics data, the above tested genes are expressed similarly between sexes, with the exception of *Gal* (female-biased expression) for which both sexes were analyzed.

### METHOD DETAILS

#### Dataset curation

All publications with mouse pituitary gland data have been identified through searching PubMed, Gene Expression Omnibus (GEO) or Sequence Read Archive (SRA) using “pituitary single-cell” or “pituitary single-nucleus” terms. Associated publications were not identified for five datasets (GSE242296,^33^ GSE299835,^43^ GSE239316,^44^ GSE310493,^47^ GSE316726^46^) and metadata was exclusively extracted from GEO in these cases. All respective SRA, European Nucleotide Archive (ENA) or GEO identifiers were extracted manually, and organized in a table with available metadata, including genotype, age, sex, estrous cycle stage, assay modality, and single-cell technology (Table S1). Barcoding kits were confirmed manually by comparing a sample of R1 reads against all 10X Genomics barcode whitelists. This search revealed multiple published datasets with incorrectly stated barcoding kits in the corresponding Methods section of the respective publication, its GEO site, or in some cases both. This approach for identifying the correct barcoding chemistry is similar to that in,^51^ where the authors have also noted that barcoding chemistry is unreliable in SRA/GEO metadata.

#### Accessing transcriptomics datasets

Using the SRA IDs, raw sequencing files for datasets^8,10–41,43–47^ were obtained from the SRA using ffq^135^ and sra-tools, or manually from ArrayExpress. In some cases, we only found the R2 reads uploaded,^10,29^ which then required us to access the bam files and convert them to fastq format using the 10X bamtofastq software (https://github.com/10XGenomics/bamtofastq). The data accession workflow that requires a curated data frame as input is implemented in the atlas module of the epitome_tools Python package to enable future atlasing efforts.

#### Pre-processing - Single-cell transcriptomics

All resulting fastq files were uniformly pre-processed using the kallisto-bustools version 0.29.5 (kallisto v0.51) program^133,134^ using a workflow that quantifies nascent and coding transcripts (‘‘nac’’), which captures intronic, exonic and ambiguous reads. The pseudoalignment was against the default reference transcriptome in the command ‘‘kb ref’’ (ensembl release 108 - GRCm39). QC plots using data from the run_info.json and inspect.json output files are shown in Figure S2A.

The final count matrices for each dataset contained the sum of intronic, exonic, and ambiguous matrices. This approach is both up-to-date with the current default in Cell Ranger, and also enables a more uniform quantification across assays (previously intronic regions would only be included in single-nucleus data, but not for single-cell) as highlighted in.^51^ Output feature names contained ensembl IDs, rather than gene symbols, for various genes such as *Lhb* and *Nrg1*. Where gene symbols were available through Biomart, we substituted the ensembl IDs with gene symbols.

#### Accessing and processing the IGVF dataset

The datasets in the IGVF resource have been produced using an elaborate split-pool design, mixing donors of various mouse strains which were then deconvoluted using their known genotypic differences (see Rebboah et al.^42^). Recovering information on cells from these datasets requires reverse-engineering the split-pool design, as well as mapping to the different strain-specific reference transcriptomes. Given that the alignment software (kallisto) was the same as used in our case, we decided to retrieve the processed AnnData objects (not raw fastq files) from the IGVF data portal (IGVFFI6924ZYXS dataset from https://data.igvf.org/analysis-sets/IGVFDS1666GZSV/). Here the raw counts layer was used. Ambient RNA removal and cell filtering was performed similarly to the other datasets (see “Further processing and quality control of all sc/snRNA-seq datasets”). Given that each sample was a pituitary and diencephalon dissected together, only those cells were taken forward that the authors annotated as one of ‘progenitor cell’, ‘somatotroph’, ‘thyrotroph’, ‘lactotroph’, ‘melanotroph’, ‘gonadotroph’, ‘corticotroph’. Other cell types (e.g., endothelial cells) from these datasets were not taken forward as its unclear whether they were from the pituitary or surrounding tissue.

#### Nuclei isolation from pituitary

The flash-frozen mouse pituitary was processed as an individual sample. Nuclei isolation was performed based on a modified protocol from,^152^ refer to.^153^,^154^ Briefly, and all on ice, RNAse inhibitor (NEB cat# MO314L) was added to the homogenization buffer (0.32 M sucrose, 1 mM EDTA, 10 mM Tris-HCl, pH 7.4, 5mM CaCl_2_, 3mM Mg(Ac)_2_, 0.1% IGEPAL CA-630), 50% OptiPrep (Stock is 60% Media from StemCell cat# 07820), 35% OptiPrep and 30% OptiPrep right before isolation. Each sample (single or pooled pituitaries) was homogenized in a Dounce glass homogenizer (1mL, VWR cat# 71000–514), and the homogenate was filtered through a 40 μm cell strainer. An equal volume of 50% OptiPrep was added, and the gradient was centrifuged (SW41 rotor at 17,792xg; 4°C; 25min). Nuclei were collected from the interphase, washed, resuspended in 1X nuclei dilution buffer, and counted using a fluorescent assay on a K2 Cellometer (Revvity).

#### Sn multiome assay

Sn multiome was performed following the Chromium Single Cell Multiome ATAC and Gene Expression Reagent Kits V1 User Guide (10x Genomics, Pleasanton, CA). Nuclei were counted using propidium iodide fluorescence (Revvity Cellometer counter), transposition was performed in 10 μL at 37°C for 60 min, targeting 500–20,000 nuclei, before loading of the Chromium Chip J (PN-2000264) for GEM generation and barcoding. Following post-GEM cleanup, libraries were pre-amplified by PCR, after which the sample was split into three parts: one part for generating the snRNA-seq library, one part for the snATACseq library, and the rest was kept at −20C. SnATAC and snRNA libraries were indexed for multiplexing (Chromium i7 Sample Index N, Set A kit PN-3000262, and Chromium i7 Sample Index TT, Set A kit PN-3000431 respectively).

#### Quality control (QC) and sequencing of sn libraries

Libraries were quantified by Qubit 3 fluorometer (Invitrogen), and quality was assessed by Bioanalyzer (Agilent). Equivalent molar concentrations of libraries were pooled, and the reads were adjusted after sequencing the pools in a MiSeq (Illumina). The libraries were then sequenced on a NovaSeq 6000 (Illumina) at the New York Genome Center (NYGC) following recommendations from Illumina and 10X Genomics. Refer to^153^ for details.

#### Processing multiome datasets

In accordance with the rest of the atlas, the RNA part of the generated multiome datasets were aligned using kallisto-bustools and further processing and QC was done as for the other datasets.

The ATAC part of the datasets was aligned using Cell Ranger ATAC 2.0, and the rest of the processing and QC was the same as described for the other datasets.

In our analysis, we have found that the P4 male multiome dataset exhibits a relatively high percentage of doublets (~24.9%), which was likely a result of experimental parameters (e.g., loading rate).

#### Further processing and QC of all sc/snRNA-seq datasets

In each dataset, to separate true cells from cell-free droplets, we first filtered to the top 40000 barcodes, which we empirically found close to the knee of the knee plot (log_UMIs vs. log_rank plot), given that all datasets ranged between 500 and 20000 cells. Following this, we used the filter command from the mx package,^52^ which fitted two Gaussians on the data to separate true cells and contaminated droplets. In some cases, mx filter gave too few cells (<2000), often with a very high minimum number of UMIs (e.g., >10000). In such cases, a threshold of >1500 UMIs was enforced, and ‘‘true cells’’ were assigned again.

Knee plots following the initial cell identification are shown in Figure S2B.

We performed QC on a per-dataset basis with scanpy^136^ using median ± X*median absolute deviation filters (X = 4–5 for different metrics). Percentages of ribosomal, mitochondrial and *Malat1* counts were determined using the scanpy calculate_qc_metrics() function with “*Rps*”, “*Rpl*”, or “*mt*-” or “*Malat*” flags. Cut-offs were different for single-cell vs. single-nucleus assays (single-nucleus also encompassing multiome datasets). Specifically, these included: percentage of mitochondrial counts (max_sc = 25, max_sn = 5, X_sc = 5), percentage of ribosomal counts (max_sc = 30, max_sn = 10, X_sc = 5), percentage of MALAT1 counts (max_sc = 30, X_sc = 5), percentage of counts in top 20 genes (X_sc = 5, X_sn = 5), total counts (min_sc = 1000, min_sn = 1000), genes detected (min_sc = 800, min_sn = 800), log1p total counts (X_sc = 4, X_sn = 4), log1p genes detected (X_sc = 4, X_sn = 4).

Filtering functions are implemented in the atlas module of the epitome_tools Python package to enable future atlasing efforts.

Following filtering, doublets were identified and removed using the Scrublet^62^ algorithm on a per-dataset basis using the implementation in scanpy with default settings.

The effects of filtering steps are shown across datasets on Figures S2C and S2D. In addition, median qc statistics of the finalized datasets are also shown in Figure S2E.

Ambient RNA was then removed using scAR^144^ on a per-dataset basis for the top 100 most contaminating transcripts, as determined from cell-free droplets. We relied on the official documentation at https://scar-tutorials.readthedocs.io/en/v0.6.0/tutorials/scAR_tutorial_denoising_scRNAseq.html. We estimated the ambient profile in each dataset using the following function:


setup_anndata(



 adata = adata,



 raw_adata = adata2,



 prob = 0.99,



 min_raw_counts = 1, iterations = 1,



 kneeplot = True)


We found scAR to overestimate the contribution of ambient transcripts (often entirely removing them during correction) and therefore applied shrinking (toward the median value of proportional contributions) to balance this effect. scAR was run for 600 epochs, with batch size = 50 and with a target of achieving 0.5% increased sparsity in the expression matrices. This left us with the original values for all transcripts, except for changes in the top 100 contaminating transcripts, often with most hormone transcripts in the top 5. Notably, a recent benchmarking study of ambient RNA removal approaches found that scAR successfully removes the most ambient RNA across methods, but does indeed overcorrect, and removes true RNA abundance as well.^155^ Overall this reinforces our rationale for using scAR to remove the highly abundant contaminating hormone transcripts, but only applying the correction to the top 100 ambient transcripts.

QC plots showing the effects of ambient RNA removal across datasets are shown in Figures S3A and S3B.

Following this, we performed an initial round of cell typing using CellAssign^145^ with a curated cell type marker matrix of established pituitary markers. These were all taken from domain knowledge of pituitary physiology, main dot plots in publications, or from supplementary tables of highly significant marker genes in previous studies. Only genes that occurred at least twice across resources were used. Specifically, we used the following genes (in no specific order):

Stem_cells: *Sox2, Rbpms, Mia, Aqp3, Krt8, Krt18, Lcn2, Cyp2f2, Aldh1a2, Folr1, Pla2g7, Aldoc, Mgst1, Glul*

Corticotrophs: *Pomc, Crhr1, Tbx19, Gpc5, Tnt1, AW551984, Atp1a2*

Melanotrophs: *Pomc, Tbx19, Pax7, Oacyl, Pcsk2, Pkib, Megf11, Esm1, Etv1, Ascl1, Sparcl1*

Gonadotrophs: *Nr5a1, Lhb, Fshb, Spp1, Tgfbr3l, Gnrhr*

Somatotrophs: *Pappa2, Car10, Rxrg, Gh, Ghrhr*

Lactotrophs: *Prl, Hepacam2, Edil3, Angpt1, Olfm1, Six6*

Thyrotrophs: *Shox2, Dio2, Ttr, Rbp4, Trhr, Tshb*

Endothelial_cells: *Pecam1, Plvap, Igfbp7, Igfbp3, Emcn, Flt1*

Mesenchymal_cells: *Col1a1, Pdgfra, Ogn, Dcn, Inmt, Lum*

Pituicytes: *Fgf10, Rax, Scn7a, Gpc3, Nkx2-1*

Immune_cells: *Cd4, Tyrobp, Cd14, Trbc2, Cd68, C1qa, C1qc, Lyz2*

Erythrocytes: *Hba-a1, Hba-a2, Hbb-, Hba-x, Hbb-bs, Hbb-bt*

Following this coarse cell typing, all datasets were integrated using scVI. Initial cell type annotations were then smoothed using the 100 nearest neighbors of each cell obtained in the scVI latent space. Specifically, cell type values were adjusted if at least two-thirds of neighbors belonged to another cell type. This was repeated three times, reassigning the cell type of 48902 cells (about 5% of the RNA portion of the atlas). This step was applied to correct for dataset-specific inaccuracies introduced by CellAssign. Lastly, we noted a cluster in the scVI embedding which represented a mixture of cell types, indicative of low-quality cells that were retained past QC. We then found that most of these cells came from diphtheria toxin ablated samples^17,18,38^ marking truly dying cells. These cells were removed from pseudobulking, and downstream analysis. In addition, we observed that some low-quality cells scattered in the embedding were mis-annotated as “Erythrocytes”. To only keep real erythrocytes, we have removed those with less than 1 count for *Hbb-bt* (which appeared to correctly identify the diffusely-spread low-quality cells). However, these cells are still retained (to be as consistent with previous work as possible) in the individual downloadable datasets shared on *epitome*.

### Dataset inclusion criteria for analyses

Only datasets with ≥300 total cells were taken forward for any analysis. From core samples, this removed SRX6699503, SRX7874743 and SRX7874744. In addition, the 3 embryonic and 4 organoid samples were not used in any differential expression analysis (though the organoid samples were used for benchmarking the Cell Type Model). However, all datasets are available on the epitome platform (epitome-atlas.com).

From the IGVF samples, 7 were filtered out as no pituitary cells were detected.

### Accessing and processing chromatin accessibility datasets

Chromatin-accessibility data were obtained from Cell Ranger output files (specifically fragments.tsv files) from GEO for all studies.^11,21–23,28,36,45^ For one study,^21^ two samples were found to have been merged into a single count table. For these samples specifically, we realigned the reads using Cell Ranger ATAC 2.0 to generate two separate datasets. In another study, two donors had two biological replicates sequenced, but fragment files were only available for one in both cases.^36^ We accessed raw files for all four (2 × 2 replicates) and realigned these using Cell Ranger. All Cell Ranger fragment files were then loaded into SnapATAC2^137^ with the initial filter min_num_fragments >1500.

We performed QC using a median ±3*median absolute deviation filter, for log_n_frags. In addition, a hard cut-off of >7 was used for per cell transcription start site enrichment. For each dataset, fragments were initially counted across genomic bins of 5000bp, and following the selection of the top 30000 features, Scrublet was used to identify and filter out doublets, leaving us high-quality cells. Changes in cell numbers following filtering are tracked in Figure S4A.

Once high-quality cells were identified, peak calling was repeated with MACS3^156^ as implemented in SnapATAC2. Following this, peaks were filtered against the problematic regions from the ENCODE blacklist described in^157^ using the file from https://github.com/Boyle-Lab/Blacklist/blob/master/lists/mm10-blacklist.v2.bed.gz with the SnapATAC2 function snapatac2.pp.select_features(). Notably the blacklist filtering only removed 3271 (about 1.3% of all) peaks, suggesting that blacklist regions are generally not problematic in snATAC-seq assays. The resulting peaks were then merged using a uniform 500 bp width, giving a Consensus Chromatin Landscape (CCL). Peaks were retained if they were independently called by MACS3 in ≥10 datasets. Quantification was then performed by counting fragments (rather than reads, for reasons discussed in^147^) falling in each peak. Peaks in the Consensus Chromatin Landscape were compared with dataset-specific peaks by counting the fragments in each set of peaks, and comparing them to fragments found in all genomic tiles/bins before peak calling (total fragment count) (Figure S1A). This revealed that CCL peaks capture an additional ~5.7% of all sequenced fragments compared with dataset-specific peaks.

To examine study-specific peaks in the CCL, we have calculated the percentage of CCL peaks that would be identified independently in each study and dataset (Figure S1B). In addition, we tested dropping out each study to see its effects on the CCL (Figure S1B). Across these analyses, we have observed no evidence for study-specific biases in CCL peaks. Lastly, we made pairwise comparisons of pseudobulk accessibility profiles across all studies (studies were further split by sex) (Figure S4B). This identified high correlation (Spearman correlation coefficient >0.79) in fragment counts across peaks in all pairs of studies. Importantly, differences between sexes in the same study (e.g., see Ruf-Zamojski et al. Male vs. Ruf-Zamojski et al. Female) were larger than differences between studies of the same sex (e.g., see Ruf-Zamojski et al. Male vs. Schang et al. Male). Overall, this demonstrates little to no study-specific effects (lower than sex-bias) on accessibility profiles. Furthermore, at least 99.80% of CCL peaks were available in all pairwise comparisons, suggesting that none of the studies exhibit a highly distinct accessibility profile.

For cell typing, we integrated all cells using poissonVI,^147^ and used cells from multiome studies as a bridge to transfer annotations previously made on the RNA level. Specifically, each cell was annotated by a “majority vote” system based on their 100 nearest neighbors in the poissonVI latent space. This step was repeated twice, such that all cells were then assigned to a given cell type. For robust analysis, we then pseudobulked each cell type in every dataset using DecoupleR, such that each cell type was only pseudobulked if it had ≥100 cells in a given dataset.

To ensure that cell types are similarly represented across CCL peaks, we counted peaks with at least 1 fragment mapped in each pseudobulk across cell types (Figure S1C). In addition, we have also calculated the average fragment count in pseudobulks across cell types (Figure S1C). Both of these analyses suggested that cell types are similarly covered by CCL peaks, despite the large differences in cell type abundance (somatotrophs and lactotrophs are highly overrepresented in numbers).

A bed file of CCL peaks can be downloaded from https://github.com/Andoniadou-Lab/consensus_pituitary_atlas/blob/main/Consensus_Chromatin_Landscape/consensus_chromatin_landscape.bed or from epitome-atlas.com in the downloads tab.

### Per-dataset QC reports

For all RNA and ATAC datasets we have generated one-page reports containing figures that illustrate the filtering steps. A compiled PDF is available on doi:10.5281/zenodo.18472039, while individual QC reports can be accessed on epitome-atlas.com.

### scVI, scANVI and poissonVI models

To jointly analyze single cells from all transcriptomic datasets, we used the scVI variational auto-encoder framework.^146^ Specifically, we set each SRA_ID (one per dataset) as the batch_key, and set Comp_sex (computationally corrected sex assignment) and technology (single-cell, single-nucleus, multiome RNA, Parse) as categorical covariates. To increase mixing between technologies, percentages of mitochondrial, ribosomal, and intronic reads (using *Malat1* as proxy^158^) were added as continuous covariates. Furthermore, to correct for age-dependent gene expression changes, we added “Age_numeric” as an additional continuous covariate. The model was set up using 2 hidden layers and a latent space with 30 dimensions. The generative end of the model was specified to be negative binomial, following considerations of the classic two-telegraph bursty transcription model. The model was then trained for 100 epochs (until convergence). Cell type smoothing was performed on this scVI embedding. For UMAP visualizations (displayed in Figure S5 and on epitome-atlas.com), we have further trained our scVI model within the scANVI^159^ framework for another 50 epochs.

For chromatin accessibility datasets, we used the poissonVI variational auto-encoder framework,^147^ as implemented in scVI-tools.^146^ Here we also set SRA_ID (one per dataset) as the batch_key, and set sex and technologies (single-nucleus, or multiome ATAC) as categorical covariates. The model was set up using 2 hidden layers and a latent space with 30 dimensions, and a Poisson likelihood function (hence its name).

The quality of dataset integration was qualitatively assessed with UMAPs (Figures S5 and S6) and more quantitatively using the scib-metrics package^148^ as shown in (Figures S5 and S6).

### UMAP visualizations

Despite the known caveats of UMAP plots^160^ we have generated these to display mixing cells across various covariates (sex, experimental procedure, datasets, publications) in the learned latent space by scANVI and poissonVI. UMAPs were generated using the scanpy functions sc.pp.neighbors(adata), sc.tl.umap(adata, min_dist) and sc.pl.umap(adata) with default settings unless otherwise specified. The argument min_dist was set as 0.2 for the ATAC UMAP and 0.3 for the RNA UMAP.

### Dot plot construction

Generating dot plots for an entire atlas has the risk of introducing biases from datasets with higher cell counts. To ensure that datasets contribute evenly, we calculated the proportion of expression (dot plot circle size) and expression magnitude (dot plot circle color - calculated as mean log1p(CP10K)) per dataset. We then calculated the mean proportion of expression across datasets, and the mean of magnitude values. Because the mean values were calculated on a per-dataset basis, the aggregated means should not be biased by cell type abundance of individual datasets. These values were used to construct dot plots on the *epitome* platform, and all dot plots in the manuscript were exported from there, meaning they can also be reproduced by the reader.

### Pseudobulk differential expression analysis

From each sc/snRNA-seq dataset, pseudobulk (summing all counts per cell type per dataset) versions were generated using decoupleR,^150^ which were merged in a final object. Only cell types with ≥50 cells were pseudobulked in each dataset. We performed differential expression analysis for cell type markers and sex- and age-dependent effects using limma^138^ and dream,^140^ referred to as “limma/dream” workflow hereafter. Similar workflows have been adopted by others using limma,^161^ or pyDEseq2.^42^ The limma/dream workflow was chosen because it accommodates a mixed-effects model and also has a significant advantage in handling large datasets (>1000 pseudobulk samples in this case). Here each sample was normalized by its constituent number of cells, and then multiplied by the median number of cells across all samples. We used the filterByExpr function to remove low-expression genes. Then, TMM normalization within limma was used. Following this, a mixed-effects design was pursued where study and modality were defined as random effects. We have included these factors as random effects following variance partition analysis with the variancePartition^139^ package. Specifically, this revealed a 17.9% median percentage of variance explained by “Study” (levels for each study) and 11.3% for “Modality” (levels for “sc”, “sn”, “multiome”) (Figure S11A). The following model was used to retrieve marker genes: ~0 + cell_type + (1|study) + (1|modality), with contrasts defined between every pair of cell types.

In the analysis presented here, we defined “cell typing markers” as genes that might have high utility for annotating pituitary datasets in the future, or for planning spatial transcriptomics experiments. These were determined based on two criteria.

- Significantly higher expression in cell type X, compared with all other cell types (including non-anterior-pituitary cells, such as mesenchymal cells, immune cells, endothelial cells, pituicytes)
- Expression difference with a fold change value of 4 (equivalent to log2 fold change of 2).

We also defined “lineage markers” which were evaluated only for cell types that belong in the anterior pituitary lineage, and comparisons were made for cell types at various branchpoints according to the current model for the lineage hierarchy. These included 8 such comparisons, between

1. Stem cells vs. Differentiated cells
2. Gonadotrophs vs. Stem cells and other differentiated cells
3. Corticotrophs/Melanotrophs vs. Stem cells and other differentiated cells
4. Melanotrophs vs. Corticotrophs
5. Lactotrophs/Somatotrophs/Thyrotrophs vs. Stem cells and other differentiated cells
6. Lactotrophs vs. Somatotrophs/Thyrotrophs
7. Somatotrophs vs. Lactotrophs/Thyrotrophs
8. Thytrotrophs vs. Somatotrophs/Lactotrophs

In each case, for a gene to be a lineage marker, it had to be significantly (FDR adjusted *p*-values <0.05, and absolute log_2_FC > 0.5) different between the left-hand cell type(s) compared with every single right-hand cell type separately and with the same direction. In other words, we took the signed intersection of all differential expression results (and not the union), resulting in stringent markers.

For the number of replicates used in each comparison, refer to quantification and statistical analysis.

### Modeling the effects of perturbations

Given that the atlas includes various physiological, dietary and genetic perturbations, we were interested in deriving results for these. A considerable challenge in calculating differentially expressed genes for such experiments is that most of them include only 1 replicate for a given condition. We therefore only addressed two perturbations, one experiment analysing a *Gata2* conditional knockout,^22^ and multiple experiments with high-fat diets.^16,20,31,46^

To model the effect of *Gata2* conditional knockout, we used limma-voom on the 3 WT vs. 3 cKO experimental setup using the formula: ~0 + assignments + assignments:gata2_cko. Results are included in Table S4.

To model the effect of high-fat diet, we considered samples from 4 studies and used the limma/dream workflow, which allowed us to include study/author as a random effect. Here we used the following model: ~0 + assignments + assignments:hfd + (1 | Author). Results are included in Table S10.

### Correcting sex information

Following processing of the data, we checked whether the available metadata on sex was consistent throughout transcriptomic datasets. To this end, we constructed a logistic regression model using scikit-learn^149^ on 3 sex-specific genes, the X-linked gene *Xist*, and two Y-linked genes *Ddx3y* and *Kdmd5*. Re-running the model on all samples highlighted outlier samples, that indicated switching of sexes. Corrections were applied as follows:

SRX21170825: female -> male

SRX21986110: female -> male

SRX21986112: male -> female

SRX29174028: female -> male

SRX29174029: male -> female

Two of these (SRX21986110, SRX21986112) samples came from a publication that directly studied sex-specific effects of high-fat diet.^20^

For chromatin accessibility datasets, we plotted fragment counts mapping to the Y chromosome and observed that sexes reported by authors matched the expected pattern, such that only male samples had fragments mapping to the Y chromosome. For these data, no corrections were required.

### Correcting assay modality information

Similar to correcting metadata on sex, we have constructed a logistic regression model to predict assay modality, specifically whether a sample was single-cell or single-nucleus RNA-seq. Multiome samples were removed from this analysis, as those are identified by having an ATAC counterpart, and therefore are not prone to mislabeling. This identified only 3 datasets (SRX8489840, SRX8489841, SRX8489842) that were originally labeled as single-nucleus, but are predicted to be single-cell. Discussion with the authors from the study of origin^11^ revealed that these three samples were from early optimisation of single-nucleus protocols, which might explain their unexpected single-cell-like profile. Nevertheless, for filtering and processing these samples were considered single-cell.

### Age/sex-dependent expression analysis

To identify age or sex-dependent gene expression changes the following models were used: ~0 + cell_type + cell_type:Age_numeric with limma-voom and ~0 + cell_type + cell_type:Sex + (1|modality) with limma/dream. In the age-dependent analysis only single-cell (not single-nucleus/multiome) data was used, therefore modality was not included as a random effect. In the sex-dependent analysis, only single-cell and single-nucleus (not multiome) datasets were included and “modality” was included as a random effect. From single-nucleus datasets, samples from Rebboah et al.^42^ were removed, as some samples showed unclear expression patterns of ground-truth sex-biased genes (*Xist*, *Tsix* etc.), likely originating from some contamination during the split-pool assay used in that study.

### Temporal patterns analysis

In our age-dependent gene expression analysis, we observed that most of the age-dependent effects rely on the inclusion of young samples (<P10). To address temporal patterns rigorously, we have considered that genes can either exhibit a (i) steady-change, (ii) stagnation-then-change, or (iii) change-then-stagnation behavior. Importantly all three of these can occur for both genes that overall decrease or increase with age, making a total of 6 possible temporal patterns. Following the limma/dream analysis, we grouped hit genes for each cell type into these temporal patterns (see analysis on stem cells in Figure S10). Specifically, we fit a simple linear model and a segmented regression model using the segmented R package. In the segmented regression model, there is some initial slope s1, and after some breakpoint age x_bp, there is a second slope s2. We then performed model selection between the linear and segmented models using the Akaike Information Criterion. If the linear model was selected (AIC_linear < AIC_segmented), the patterns “Increase steadily” or “Decrease steadily” were chosen. For increasing genes, if the segmented model was selected, we assigned an “Increase-stagnate” pattern, if s1>s2, and “Stagnate-increase” pattern if s2>s1. Similarly, for decreasing genes, we assigned a “Decrease-stagnate” pattern if abs(s1)>abs(s2) and a “Stagnate-decrease” pattern if abs(s2)>abs(s1). The temporal pattern associated with each age-dependent gene in the temp_pattern column in Table S5.

### Gene expression box plot, and age-dependence scatter plot construction

Counts per million values for each transcript were calculated following TMM-correction in limma-voom and normalizing counts to a million. Subsequently, a pseudocount of 1 was added to the count matrix and log10 transformation was performed. These values were used to construct box plots, and age-dependence scatter plots on the *epitome* platform. All such plots in the manuscript were exported from the epitome website (epitome-atlas.com), meaning they can also be reproduced by the reader. Specifically, the sex-dependence plots can be reproduced with the “Reproduce sex-specific analysis” option ticked, while the “Reproduce age-dependent analysis” setting allows reproducing the age-dependence plots.

### Differential accessibility analysis

Pseudobulk analysis was carried out in limma-voom to identify cell type-specific open chromatin peaks. Variance partition analysis with the variancePartition^139^ package showed minimal percentage of variance explained by “Study” (median: 3e–12) (Figure S12A). Therefore we proceeded by simply using limma-voom, rather than limma/dream as for RNA-seq. Each pseudobulk sample was normalized by its constituent number of cells, and then multiplied by the median number of cells across all pseudobulk samples. We used the filterByExpr() function to remove low-accessibility peaks. Then TMMwsp normalization within limma was used. The following model was used to retrieve marker peaks: ~0 + assignments.

Lineage marking peaks were derived using the same methodology used for lineage marker genes.

Given the lack of samples with young or old ages, we did not test for accessibility changes with age, however, we were able to look at sex differences. This was performed with the following model ~0 + assignments + assignments:Sex.

### Gradient boosting models with XGBoost (Doublet Model and Cell Type Model)

The Doublet and Cell Type models were built using the XGBoost gradient boosting framework,^61^ because of its ease of use and robustness. Before training, we normalized all datasets as log1p(CP10K).

For the Doublet Model, we performed feature selection by running XGBoost on one dataset from every study (except those held-out), following which we have identified the most important 1500 features across these results, using “model.feature_importances_”. We then subsetted for these features as well as the previously identified Cell Typing markers.

Simulated doublets were generated for all datasets separately, to avoid introducing doublets from pairs of cells coming from different experiments. For example, this would have introduced male-female doublets, leading the model to learn to identify those, rather than learning general doublet properties. Specifically, we looked for all cell types with ≥25 cells in a given dataset, and sampled 150 pairs of cells with replacement (e.g., the same cell was allowed to pair up again with another cell, until reaching 150 total chosen cells) for each pair of cell types. Importantly we did not simulate doublets coming from the same cell type (known as homotypic doublets), as these cannot be discriminated from true cells (singlets). Using this approach, we generated a balanced training dataset of 936,300 simulated doublets and 907,380 real cells (singlets).

We ran XGBoost with the following hyperparameters to fit models


params = {

 ’n_estimators’: 300,

 ’max_depth’: 12,

 ’subsample’: 0.8,

 “min_child_weight”: 2,

 ’learning_rate’:0.1,

 ’colsample_bytree’: 0.7,

 ’colsample_bylevel’: 0.7,

 ’colsample_bynode’: 0.7,

 ’objective’: ’binary:logistic’,

 ’random_state’: 42,

 ’eval_metric’: ’logloss’,

 ’tree_method’: “hist”

}

In addition, we used the GroupKFold() function in scikit-learn for cross-validation with 5 splits, and groups being source publications. We found this to be a more faithful representation of performance over splitting randomly on cells or even datasets, as there is some correlation across cells assayed in the same dataset and even in the same publication. In each fold we observed successful convergence and a lack of overfitting by plotting training and validation loss after sequentially built trees.

For later use, we took the model from the first fold of the 5-fold cross validation. This is because we performed TPR/FPR calibration on this fold of the model, and the prediction threshold would have slightly changed upon retraining the model on the entire atlas.

During cross-validation, we took the direct model output; however, for later evaluations in the manuscript we applied smoothing to cell type labels based on the closest 10 neighbors in PCA space. If ≥ 6 of the neighbors had the same initial cell type assignment, then that was assigned to the cell as a final label. This correction increased consistency on dimensionality reduction plots. In the evaluations we automatically set every missing gene (not present in the dataset as it was filtered out) to zero.

For evaluation, we picked three high-quality whole-pituitary datasets from three different labs,^11,15,17^ and generated doublets comprising 10% of the total dataset. A 10% doublet rate is typical for single-cell experiments, we therefore created a fair setup for comparison with Scrublet.^62^ These doublets were simulated by randomly pairing cells of different cell types. Because of the random sampling we assumed that the resulting proportion of doublet types will reflect those in real experiments. In addition, this sampling approach allowed us to repeat this *in silico* experiment 20 times, to derive accurate estimates for the performance of the two approaches (Doublet Model vs. Scrublet). Importantly, in each iteration we subsampled the dataset by 80%, and only then generated the 10% doublets. The 80% subsampling was introduced to sufficiently differentiate between rounds of simulations, particularly by introducing a large variability in rare cell types. We ran the scanpy implementation of Scrublet with default parameters on raw counts.

For the Cell Type Model, we performed feature selection by running XGBoost on one dataset from every single study (except those held-out), following which we have identified the most important 1000 (3000 for ATAC) features across these results, using “model.-feature_importances_”. We then subsetted for these features as well as the previously identified Cell Typing markers. Importantly we removed hormone transcripts as these are typically the noisiest in every dataset. This should facilitate easier adoption by the community even if ambient RNA is not sufficiently removed by users.

We ran XGBoost with the following hyperparameters to fit models

params = {

 ’n_estimators’: 200,

 ’max_depth’: 8,

 ’subsample’: 0.8, “min_child_weight ”:5,

 ’learning_rate’:0.05,

 ’colsample_bytree’: 0.7,

 ’colsample_bylevel’: 0.7,

 ’colsample_bynode’: 0.7,

 ’objective’: ’multi:softmax’,

 ’num_class’: len(label_encoder.classes_),

 ’random_state’: 42,

 ’eval_metric’: ’mlogloss’,

 ’tree_method’: “hist ”

}

Here too, we used the GroupKFold function in scikit-learn for cross-validation with 5 splits, for reasons discussed above. The confusion matrix was calculated in the first fold of the cross-validation, however, for later evaluations we use a version of the model that was re-trained on the whole atlas (excluding held-out datasets). In each fold we observed convergence and a lack of overfitting by plotting training and validation loss after sequentially built trees.

For interpretability purposes, we extracted the top global features from the final model using.

“model.feature_importances_”. Dot plot of the top features for the Cell Type models is shown in Figure S7E.

### Doublet Model and Cell Type Model implementation

The models are implemented in the workflow module of epitome_tools Python package, which also facilitates cell typing and doublet detection on the epitome platform. We recommend users perform the joint workflow using:

from epitome_tools.workflow import celltype_doublet_workflow

annotated_adata = celltype_doublet_workflow(

 adata,

 active_assay=data_type,

 modality=“rna”

)

Here active_assay can take the value of “sc”, “sn” or “multi_rna” for single-cell, single-nucleus and multiome data respectively, while modality can take the values “rna” and “atac”. Notably, this function expects the data in a normalized (to 10K transcripts or fragments) and log-transformed (log1p) format. Throughout all evaluations, we used this programmatic implementation of the model.

We provide a Jupyter Notebook for demonstration purposes (https://github.com/Andoniadou-Lab/epitome_tools/blob/main/cell_typing_demo.ipynb), showing how to apply celltype_doublet_workflow to datasets. In this demo, we have obtained counts for datasets GSE120410 and GSE239390 directly from GEO. As these datasets have been pre-processed differently from our workflow (and lack ambient RNA removal), they provide a good benchmark for the general performance of the Cell Type model.

### Manual annotation of cell types

To benchmark the Cell Type Model we compared its performance against manual annotation. Cell type annotation is typically done through iterations of unbiased clustering, plotting of known marker genes, and merging clusters, to annotate each cell type.

We tasked a co-author (O.S.) to analyze each dataset and return it with labels from the following categories: Corticotrophs, Gonadotrophs, Lactotrophs, Melanotrophs, Pituicytes, Somatotrophs, Stem cells, Thyrotrophs, Immune cells, Mesenchymal cells, Endothelial cells, Erythrocytes. We then ran the Cell Type Model with default settings and compared the predicted labels on the same cells.

### Model evaluation and definitions

Throughout the manuscript we use various machine learning terms which are defined below.

True positive rate (TPR): The proportion of true cases that were identified as positive by the classifier.

False positive rate (FPR): The proportion of negative cases that were identified as positive by classifier.

Accuracy: Percentage of correct classifications.

Recall: Synonymous with the TPR, here used for multi-class classification. The proportion of true instances of a class that were correctly identified by the classifier.

Receiver Operating Characteristic (ROC) Curve: Varying the decision threshold of the binary classifier results in different pairs of TPR/FPR values. The ROC curve plots (TPR against FPR) all such pairs using each prediction probability as a potential threshold.

Area under the ROC curve: Metric used to evaluate binary classifiers, ranging between 0.5 and 1 for naive and perfect models respectively. The value can also be interpreted as the probability that the classifier will correctly distinguish between any pair of positive and negative cases. The ROC curve was calculated using the roc_curve() function in scikit-learn.

Overlap in labels was calculated as the fraction of cells that share the same label over all cells. For the multiome data, this was evaluated only on cells that were present in both the ATAC and RNA parts of the data.

Confusion matrices were always row-normalized (e.g., divided by the total number of true values for a given class). This returns recall values on the diagonal, which describe the number of cells that were correctly identified from each true class.

Model uncertainty was calculated as 1-P_max_, where P_max_ is the probability assigned to the most likely class. Using this definition, model uncertainty can range between 0 and 1-1/n_classes_ (in this case, 12 classes: ~0.9167), as a fully uncertain model would predict all classes with the same probability.

### Processing bulk RNA-sequencing data

Bulk RNA-sequencing datasets were retrieved as described for single-cell datasets. To be consistent with the single-cell results, the same reference genome (ensembl release 108 - GRCm39), pseudoalignment software (kallisto-bustools) and differential expression analysis workflow (limma-voom) were used. All bulk datasets are curated in Table S13. Only those genes were visualized that reached significance in the CPA (except in Figure 3L, showing non-significant genes as well). Directional agreement was then evaluated for only those genes that reached significance in both the CPA and the bulk dataset as well. This ensured that only those genes are considered that had sufficient evidence on their direction in both datasets.

### Gene set enrichment

Enrichment of hit genes was performed using EnrichR^142^ with a set of *a priori* determined (to avoid cherry-picking results) databases: Reactome Pathways 2024, Wikipathways 2024 Mouse, and GO Biological Process 2023. All significantly enriched terms for stem cell lineage markers are found in Table S8.

### Ligand-receptor interaction prediction

Ligand-receptor interaction prediction was performed using LIANA+.^91^ This approach was chosen as it allows using an ensemble of ligand-receptor algorithms, as well as an integrated ligand-receptor interaction database. Here 6 algorithms (CellPhoneDB with 1000 permutations, CellChat with 1000 permutations, Connectome, log2FC, NATMI, SingleCellSignalR) were used as implemented in the aggregate_rank() function using a merged version of three databases (LIANA CellPhoneDB, LIANA CellChatDB, and CellPhoneDB 5.0.0). The difference between LIANA CellPhoneDB and CellPhoneDB 5.0.0 was that the latter was directly accessed from the CellPhoneDB website (https://www.cellphonedb.org/), however, both resources contained important interactions not covered by the other. By default LIANA+ performs robust rank aggregation across algorithms; however, it has no option to handle multiple datasets. We therefore run LIANA+ separately (so as not to bias results by dataset size) for all wild-type, whole-pituitary transcriptome datasets (*n* = 160). This resulted in 160 lists of magnitude (how strong the expression is) and specificity (how unique this ligand-receptor signaling is across cell type pairs) values for all interactions. Following the LIANA+ step, individual lists of interactions were aggregated using a reimplementation of robust rank aggregation (RRA), which follows that in LIANA+, as well as in the original RRA paper.^92^ We used only whole-pituitary samples so as not to bias the results of robust rank aggregations with rankings from FACS-sorted datasets. Hits were called for genes with adjusted ρ values (these are the outputs of RRA) for magnitude and specificity ranks <0.05.

### Annotating genes as TFs, ligands and receptors

TF annotations were retrieved from the list of genes found at: https://esbl.nhlbi.nih.gov/Databases/KSBP2/Targets/Lists/TranscriptionFactors/. This database includes 1473 TFs. Ligands and receptors were annotated using the same curation that was used for the LIANA+ analysis.

### TF motif enrichment

We performed enrichment for TF binding motifs in differentially accessible peaks with Signac’s FindMotifs() function^141^ and using the JASPAR2022 database^162^ of TF binding motifs. This database includes 841 non-redundant vertebrate TF motifs.

### TF co-occurrence analysis

TF motif positions have been identified using the AddMotifs() Signac function using the “BSgenome.Mmusculus.UCSC.mm10” genome, and the JASPAR2022 CORE vertebrates motif collection. Following this, we have counted the co-occurrence of each motif alone and estimated the frequency of co-occurrence by chance. This was then compared with the observed co-occurrence using a Fisher’s exact test. As an example, for TFs “A” and “B”, we used a 2 × 2 contingency table with columns “A occurring” and “A not occurring”, and rows “B occurring” and “B not occurring”.

### Mapping motifs to annotated genomic regions

Following extraction of motif positions, we used the annotatr package^143^ to assign genomic annotations to motif positions using the build_annotations() (with mm10 genome and the following annotation groups: ‘mm10_basicgenes’, ‘mm10_cpgs’, ‘mm10_genes_intergenic’, ‘mm10_genes_cds’, ‘mm10_genes_firstexons’, ‘mm10_genes_intronexonboundaries’, ‘mm10_genes_exonintronboundaries’, ‘mm10_lncrna_gencode’, ‘mm10_enhancers_fantom’) and annotate_regions() functions and summarized with the summarize_annotations() function.

### RNAscope mRNA *in situ* hybridization

RNAscope mRNA *in situ* hybridization experiments were performed using the RNAscope 2.5 HD Duplex Reagent kit (ACD Bio #322430) as per manufacturer’s instructions. Stained sections were mounted in VectaMount Permanent Mounting Medium (Vector Laboratories, H-5000-60). The following probes were used: *Nrg1 (#418181), Erbb4 (#318721-C2), Slit2 (#449691), Robo1 (#475951-C2), Nr5a1 (#445731-C2), Nhlh2 (#527811), Sox2 (#401041-C2), Rfx4 (#520551), Six2 (#500011), Runx1 (#406671).*

### Immunofluorescence microscopy

Microscopy slides with mounted sections were deparaffinized and rehydrated through a descending graded ethanol series. Antigen retrieval was carried out using DeClere citrate retrieval buffer (pH 6.0) in a decloaking chamber NXGEN (Meraini Diagnostics) for 3 min at 110°C. Hydrophobic pap pen was applied around the sample and all incubation steps were carried out in a humified chamber.

Sections were incubated in Blocking Buffer (0.15% glycine, 2 mg/mL bovine serum albumine, 0.1% Triton X- in PBS), containing 10% serum (sheep or donkey) for 1 h at room temperature. Sections were then incubated overnight with the respective primary antibodies at 4°C, in Blocking Buffer with 1% serum. Primary antibodies used were against SOX2 (1:300, R&D AF2018), LEF1 (1:100, Cell Signaling 2230S) and GAL (1:300, Invitrogen PA5-62069). Slides were washed in PBS with Triton X-0.1% (PBST) then incubated with secondary antibodies in Blocking Buffer for 1 h at room temperature (biotinylated anti-rabbit (1:350, Abcam ab207999), anti-goat Alexa Fluor 488 (1:500, Abcam ab150129), anti-rabbit Alexa Fluor 594 (1:500, Abcam ab150080)). Where appropriate, slides were washed using PBST and incubated with fluorophore-conjugated Streptavidin (1:500, Life Technologies S32355) for 1 h at room temperature. Slides were then washed with PBST and incubated with Hoechst (1:10000, Life Technologies H3570). Slides were washed in PBST and mounted with VectaMount (Vector Laboratories, H1000).

### Imaging

RNAscope slides were scanned with Nanozoomer-XR Digital slide scanner (Hamamatsu) and the images were processed using the Nanozoomer Digital Pathology (NDP) View software. In addition, some close-up images were taken with an Olympus BX34F Bright-field microscope using a 100X objective. Fluorescent stainings were imaged with a Leica TCS SP5 or a Zeiss LSM980 confocal microscope and the resulting images were processed using the Fiji software.^151^

## QUANTIFICATION AND STATISTICAL ANALYSIS

Statistical analyses were performed with dedicated software for bulk and single-cell genomics in either R or Python, as discussed above. For all analyses, we used a *p*-value (adjusted for multiple testing where appropriate) threshold of 0.05 to determine statistical significance. Multiple-testing corrections were applied as follows: Benjamini-Hochberg correction for marker discovery and sex-analysis, and Bonferroni corrections for age-analysis. In addition, we thresholded for absolute log2 fold change in marker discovery (>0.5) and sex-analysis (>1).

Throughout the text, we use “sample” to refer to cells originating from a single experimental replicate (animal/pool) and from a single 10X lane (with exceptions for Parse split-pool technology). In practice, samples are represented by their own SRA or ENA IDs. While some samples are whole pituitaries, some are sorted for a certain cell type. In each analysis, keeping in mind the statistical formulation of the question, these samples all function as replicates. We also count multiome samples as two during our census presented in Figure 1B (1 RNA and 1 ATAC), as they contribute to both RNA and ATAC analyses separately, and are at no point treated as a single sample. In addition, multiome datasets include some cells with high-quality data only in one modality, such that the final number of cells often differs between the ATAC and RNA counterparts. As such, we argued that a more complete census is to sum up the available cells across both modalities. However, to avoid confusion, we also report the total number of independent final (passed QC) datasets with each multiome considered as a single dataset, which is 254 datasets (205 only RNA, 31 in ATAC, 18 in multiome, with 75.430 barcodes present in both RNA and ATAC).

Sample sizes for the various differential expression/accessibility analyses were as follows (note that certain cell types might not have been present in sufficient numbers in every single sample - e.g., 100 samples might not mean 100 pseudobulks for cell type X):

CPA analyses (RNA) - these do not include organoid and developmental samples:

Cell typing and lineage marker genes (all technologies, postnatal samples only): 216 samples and 19197 genes after filtering.

Age-dependent expression (single-cell, not single-nucleus/multiome, postnatal samples only): 114 samples and 20595 genes after filtering.

Sex-biased expression (10X single-cell or single-nucleus, but not multiome or Parse, samples aged between P10-P200): 71 male vs. 32 female samples and 20217 genes after filtering.

CPA analyses (ATAC):

Cell typing and lineage marker peaks (all samples): 49 samples and 213371 peaks after filtering.

Sex-dependent peaks (P4 male multiome not used - only the rest of the samples that reached sexual maturity): 27 male vs. 21 female samples and 228723 peaks after filtering.

Bulk analyses:

Age-dependent genes: 4 young vs. 4 aged samples and 15729 genes after filtering

*hpg* mice sex-biased genes: 6 male vs. 5 female samples and 15901 genes after filtering

Corticotroph sex-biased genes: 3 male vs. 3 female samples and 16787 genes after filtering

Gonadotrophs sex-biased genes: 4 male vs. 4 female samples and 16594 genes after filtering

Whole-pituitary sex-biased genes: 26 male vs. 29 female samples and 15510 genes after filtering

### ADDITIONAL RESOURCES

We have generated the electronic pituitary omics (*epitome*) platform, which is accessible at epitome-atlas.com.

## Supplementary Material

1

2

3

4

5

6

8

9

10

11

12

13

14

15

Supplemental information can be found online at https://doi.org/10.1016/j.celrep.2026.117407.

## Figures and Tables

**Table:** KEY RESOURCES TABLE

REAGENT or RESOURCE	SOURCE	IDENTIFIER
Antibodies		
Anti-GAL antibody (rabbit)	Invitrogen	PA5-62069; RRID:AB_2641826
Anti-SOX2 antibody (goat)	R&D	AF2018; RRID:AB_355110
Anti-LEF1 antibody (rabbit)	Cell Signaling	2230S; RRID:AB_823558
Anti-rabbit Biotinylated (donkey)	Abcam	ab207999
Anti-goat AF 488 (donkey)	Abcam	ab150129; RRID:AB_2687506
Anti-rabbit Alexa Fluor 594 (goat)	Abcam	ab150080; RRID:AB_2650602
Streptavidin Alexa Fluor 555	Life Technologies	S32355; RRID:AB_2571525
Biological samples		
Genetic reagent (Mus musculus) CD-1 IGS	Charles River	Strain code 022 Crl:CD1(ICR)
Chemicals, peptides, and recombinant proteins		
RNAse inhibitor	NEB	# MO314L
OptiPrep	StemCell Tech	07820
Sucrose	Sigma	S0389
EDTA	Corning	46-034-Cl
Tris-HCl, pH 7.4	Sigma	T2663
CaCl_2_	Sigma	21115
Mg(Ac)2Boston BioproductsMT-190IGEPAL CA-630	Sigma	I3021
Critical commercial assays		
RNAscope 2.5 HD Duplex Reagent kit	ACD Bio	#322430
Chromium Single Cell Multiome ATAC and Gene Expression Reagents	10x Genomics	V1
Chromium Chip J	10x Genomics	PN-2000264
Chromium i7 Sample Index TT, Set A	10x Genomics	PN-3000431
Chromium i7 Sample Index N, Set A	10x Genomics	PN-3000262
Deposited data		
P4 male mouse multiome	This paper	NCBI: PRJNA1329231
Experimental models: Organisms/strains		
Genetic reagent (Mus musculus) CD-1 IGS	Charles River	Strain code 022 Crl:CD1(ICR)
Oligonucleotides		
Nrg1	ACD Bio	#418181
Erbb4	ACD Bio	#318721-C2
Slit2	ACD Bio	#449691
Robo1	ACD Bio	#475951-C2
Nr5a1	ACD Bio	#445731-C2
Nhlh2	ACD Bio	#527811
Sox2	ACD Bio	#401041-C2
Rfx4	ACD Bio	#520551
Six2	ACD Bio	#500011
Runx1	ACD Bio	#406671
Software and algorithms		
kallisto-bustools (kb-python) - 0.29.5	Sullivan et al.^133^	https://github.com/pachterlab/kb_python
kallisto - 0.51.1	Bray et al.^134^	https://github.com/pachterlab/kallisto
mx	Booeshaghi et al.^52^	https://github.com/cellatlas/mx
ffq - 0.3.1	Galvez-Merchan et al.^135^	https://github.com/pachterlab/ffq
sra-tools - 3.3.0	National Center for Biotechnology Information (NCBI)	https://github.com/ncbi/sra-tools
scanpy - 1.11.4	Wolf et al.^136^	https://github.com/scverse/scanpy
SnapATAC2 - 2.8.0	Zhang et al.^137^	https://github.com/scverse/SnapATAC2
limma - 3.64.3	Law et al.^138^	https://www.bioconductor.org/packages/release/bioc/html/limma.html
variancePartition and dream - 1.41.3	Hoffman et al.^139^; Hoffman et al.^140^	https://bioconductor.org/packages/release/bioc/html/variancePartition.html
Signac - 1.16.0	Stuart et al.^141^	https://github.com/stuart-lab/signac
enrichR - 3.4	Kuleshov et al.^142^	https://github.com/wjawaid/enrichR
annotatr - 1.36.0	Cavalcante et al.^143^	https://bioconductor.org/packages/devel/bioc/html/annotatr.html
Cell Ranger ATAC - V2.2	10X Genomics	https://github.com/10XGenomics/cellranger-atac
scAR - 0.7.0	Sheng et al.^144^	https://github.com/Novartis/scar
Cellassign (scvi-tools implementation - 1.4.0)	Zhang et al.^145^	https://github.com/scverse/scvi-tools
scVI (scvi-tools 1.4.0)	Gayoso et al.^146^	https://github.com/scverse/scvi-tools
poissonVI (scvi-tools 1.4.0)	Martens et al.^147^	https://github.com/scverse/scvi-tools
scib-metrics - 0.5.9	Luecken et al.^148^	https://github.com/yoseflab/scib-metrics
epitome_tools - 1.0.4	This paper	https://github.com/Andoniadou-Lab/epitome_tools
epitome website and database - v_0.02	This paper	https://github.com/Andoniadou-Lab/epitome; https://epitome-atlas.com/; https://doi.org/10.5281/zenodo.17154161
QC reports for single-cell datasets	This paper	https://doi.org/10.5281/zenodo.18472039
xgboost - 3.2.0	Chen et al.^61^	https://github.com/dmlc/xgboost
scikit-learn - 1.6.1	Pedregosa et al.^149^	https://github.com/scikit-learn/scikit-learn
decoupler - 1.9.2	Badia-I-Mompel et al.^150^	https://github.com/scverse/decoupler
LIANA+ - 1.6.0	Dimitrov et al.^91^	https://github.com/saezlab/liana-py
Fiji	Schindelin et al.^151^	https://imagej.net/software/fiji
R - 4.4.1	R Core Team	https://www.r-project.org/
Python - 3.10	Python Software Foundation	https://www.python.org/
Other		
VectaMount Permanent Mounting Medium	Vector Laboratories	H-5000-60
Dounce glass homogenizer	VWR	# 71000-514
40 mm cell strainer	Falcon	352340
Centrifuge SW41 rotor	Beckman Coulter	#331362
Fluorometer	Invitrogen	Qubit 4
Tapestation	Agilent	4150

## Data Availability

• The generated multiome dataset is deposited in the Sequence Read Archive (NCBI: PRJNA1329231). Processed files and single-cell objects are available on the epitome platform (epitome-atlas.com “Downloads” tab). • The complete code for analysis is found at https://github.com/Andoniadou-Lab/consensus_pituitary_atlas; the source code for the epitome platform and the epitome_tools package is maintained at https://github.com/Andoniadou-Lab/epitome and https://github.com/Andoniadou-Lab/epitome_tools, respectively. • Any additional information required to reanalyze the data reported in this paper is available from the lead contact upon request.

## Highlights

- Uniform processing of 283 mouse pituitary single-cell datasets (~1.3 M cells)
- The statistical power enables cell-type-, sex-, and age-specific marker discovery
- Machine learning models facilitate doublet detection and cell typing in new datasets
- The *epitome* platform provides programming-free data access and visualizations
