# Supplementary material for: Consensus Pituitary Atlas, a scalable resource for annotation, novel marker discovery, and analyses in mouse pituitary gland research
Source: Cell Rep. Author manuscript; Available in PMC 2026 Jul 24. (PMC13397322; doi:10.1016/j.celrep.2026.117407)

Cell Reports, Volume *45*

Supplemental information

Consensus Pituitary Atlas, a scalable resource for annotation, novel marker discovery, and analyses in mouse pituitary gland research

Bence Kövér, Thea L. Willis, Olivia Sherwin, James Kaufman-Cook, Yasmine Kemkem, Miriam Vazquez Segoviano, Emily J. Lodge, Michel Zamojski, Natalia Mendelev, Zidong Zhang, Gregory R. Smith, Daniel J. Bernard, Hui-Chun Lu, Stuart C. Sealfon, Frederique Ruf-Zamojski, and Cynthia L. Andoniadou

SUPPLEMENTAL INFORMATION

Document S1. Figures S1-S12.

Figure S1., related to Figure 1
Figure S2., related to Figure 1
Figure S3., related to Figure 1
Figure S4., related to Figure 1
Figure S5., related to Figure 1
Figure S6., related to Figure 1
Figure S7., related to Figure 2
Figure S8., related to Figure 3
Figure S9., related to Figure 4
Figure S10., related to Figure 4
Figure S11., related to Figure 5
Figure S12., related to Figure 6

Document S2. Tables S1-S13.

Table S1: Curation of single-cell datasets, related to Figure 1
Table S2: Table of all cell typing markers, related to Figure 2
Table S3: Table of all sex-biased genes, related to Figure 3
Table S4: GATA2 results, related to Figure 3
Table S5: Table of all age-dependent genes, related to Figure 4
Table S6: Table of all lineage marker genes, related to Figure 5
Table S7: Table of all low expression markers, related to Figure 5
Table S8: Table of enrichment results for stem cells, related to Figure 5
Table S9: Table of all signaling results, related to Figure 5
Table S10: High-fat diet results, related to Figure 5
Table S11: Table of all lineage marker peaks, related to Figure 6
Table S12: Table of all TF hits, related to Figure 6
Table S13: Curation of bulk datasets, related to Figure 3 and 4

SUPPLEMENTARY FIGURE LEGENDS

Figure S1., related to Figure 1

(A) Double bar plot of the number of captured fragments using per-dataset called peaks (gray - lower) versus using the Consensus Chromatin Landscape (CCL; blue - higher).

(B) (Left) Bar plot of CCL peaks identified at least once per study. This shows that most peaks are identified at least once across all studies, except for Zhang et al.[28] (Middle) Bar plot of CCL peaks identified in each dataset (dots) across all studies (bars). This shows that every sample identifies a similar amount (~70%) of CCL peaks on its own irrespective of the study of origin. (Right) Barplot of CCL peaks identified in the atlas upon dropping out each study. This shows that the CCL is robust to removing studies and is minimally affected by study-specific peaks.

(C) (Left) Box plot of cell numbers per cell type across single-nucleus ATAC-seq datasets (each dot is a pseudobulk sample), showing the overrepresentation of somatotrophs and lactotrophs. Bar heights are showing the median values. (Middle) Box plot of the number of peaks with ≥1 fragment captured per cell type across single-nucleus ATAC-seq datasets (each dot is a pseudobulk sample). This shows that despite the overrepresentation of somatotrophs and lactotrophs, the CCL peaks represent all cell types in a balanced way. Bar heights are showing the median values. (Right) Box plot of the average fragment count per cell type across single-nucleus ATAC-seq datasets (each dot is a pseudobulk sample). This shows that despite the overrepresentation of somatotrophs and lactotrophs, the peaks represent all cell types in a balanced way. Bar heights are showing the median values.

(D) (Left) Stacked bar plot of cell type composition across all ATAC samples and (Right) mean cell type composition of male and female samples.

(E) Dot plot of canonical pituitary cell type markers using aggregated data from the CPA. This shows that canonical markers are recovered accurately following our processing and cell typing workflow.

Figure S2., related to Figure 1

(A) Quality control box plots for metrics associated with pseudoalignment using kallisto-bustools. These plots show consistent performance across datasets, with the only outliers being the DROPSEQ samples for "% reads on barcode on-list". However, as DROPSEQ datasets do not actually have a barcode on-list these values are expected and are not true outliers.
(B) Knee-plots of all datasets (except IGVF datasets from Rebboah et al.[42]) showing the retained (red) and discarded (black) cells. Overall, this shows that our automated workflow correctly identified the knee in all datasets.
(C) Box plots of changing cell numbers following filtering according to various metrics.
(D) Box plots of the number of cells tagged for removal by each metric during the filtering process.
(E) Final quality control metrics for all datasets.

Figure S3., related to Figure 1

(A) Dot plots of canonical pituitary cell type marker genes (including hormones) before and after ambient RNA removal with scAR. Dot plots were aggregated across datasets (Methods). This analysis shows that ambient RNA removal was successful in constraining hormone expression to the correct cell types.
(B) Quality control box plots for metrics associated with ambient RNA removal. These show that ambient RNA removal effects were consistent across datasets, and as expected: sparsity slightly increased, but overall, most of the counts were retained in the datasets.

Figure S4., related to Figure 1

(A) Box plots of cell numbers following quality-control filtering in the ATAC-seq datasets. This shows that filtering was not too excessive on any of the datasets.
(B) Pairwise scatter plot between pseudobulk fragment profiles of all study + sex combinations. To generate the plot, sum pseudobulk profiles were normalized to 1 million fragments and log10 transformed following the addition of a pseudocount of 0.0001. A box of values larger than 0.0001 shows the percentage of fragments detected in both studies, while dots outside of the box represent fragments with 0 counts (e.g. only the pseudocount). The fraction of peaks accessible in both studies is shown in red. All pairs of studies exhibited very high concordance in accessibility across all peaks (Spearman correlation coefficient > 0.79), and at least 99.8% of peaks were accessible in all pairs of studies. Importantly, differences between sexes in the same study (see Ruf-Zamojski et al.[11] Male vs Ruf-Zamojski et al.[11] Female) were larger than differences between studies in the same sex (see Ruf-Zamojski et al.[11] Male vs Schang et al.[22] Male). Overall, this demonstrates minimal study-specific effects on accessibility profiles.

Figure S5., related to Figure 1

(A) Panel of UMAPs showing integrated RNA dataset. First cell types were smoothed from the initial CellAssign assignments. Following this two groups of cells were removed due to their low-quality, (i) cells from DT-ablated samples[17,18,38] and (ii) cells that were mis-annotated as Erythrocytes, but did not express *Hbb-bt* - used as a ground-truth marker of Erythrocytes. The rest of the UMAPs shows final cell type assignments, and integration across modalities, RNA-seq kits, sexes and publications.
(B) scIB metrics for the integrated embeddings (scVI and scANVI) as compared to PCA. This shows an overall improvement over PCA during the integration process, with values for each metric that is within expected range.
(C) PCA of all pseudobulk samples showing that assay platform explains most of the variability across pseudobulk samples. UMAPs of pseudobulk samples colored by cell type for multiome, single-cell and single-nucleus (including 10X and Parse). These figures show that pseudobulk

samples cluster based on cell types and not based on study identity, without any integration approach.

Figure S6., related to Figure 1

(A) Panel of UMAPs showing the process of annotating the chromatin accessibility datasets. First annotations were identified for multiome cells from the RNA counterpart of each cell. Then annotations were transferred to nearby "unknown" cells that did not have an RNA counterpart. Lastly, a low-quality cluster of cells coming from mostly 3 datasets were removed. The final dataset shows good integration across publications and modalities.

(B) scIB metrics for the integrated embedding as compared to PCA. This shows an overall improvement over PCA during the integration process, with values for each metric that is within expected range.

(C) PCA of pseudobulk samples colored by assay type and UMAPs colored by cell type and sex. These plots show that pseudobulks group according to cell type without any integration approach.

Figure S7., related to Figure 2

(A) Violin plots of log1p total counts and log1p number of genes in doublets and real cells (as identified by the Doublet Model) in the newly generated P4 male multiome dataset.

(B) Scatter plot of Doublet Model doublet scores vs Scrublet doublet scores in the P4 male multiome dataset.

(C) Dot plot of canonical pituitary cell type markers in the P4 male multiome dataset annotated using the Cell Type model.

(D) Panel of UMAPs for *Tbx19*-KO sample.[35] Cell types were assigned by the Cell Type Model. The various genes shown are melanotroph markers determined using the CPA.

(E) Bar plot of feature importance ("gain") scores for the top 20 features in the cell type model.

(F) Dot plot of canonical markers for each pituitary cell type plotted in the cells from the fold 1 validation set.

(G) Row-normalized (giving recall percentages on the diagonal) confusion matrix of ATAC Cell Type Model predicted labels (X axis) and true cell type labels (Y axis).

Figure S8., related to Figure 3

(A) Volcano plot of sex-biased genes from reanalysis of male/female gonadotroph bulk RNA-seq samples,[68] colored according to sex-bias in CPA. Gonadotroph sex-biased genes *Fshb*, *Gpr101* and *Grem1* are highlighted in red.

(B) Volcano plot of sex-biased genes from reanalysis of male/female corticotroph bulk RNA-seq samples,[69] colored according to sex-bias in CPA.

(C) Box plot of *Gpr101* expression across cell types and sexes (blue: male; orange: female).

(D) Box plot of *Gata2, Grem1, and Fshb* expression in gonadotrophs showing sex-biased expression (blue: male; orange: female).

(E) Horizontal strip plot of $\log_2$ fold-changes in Ctrl vs AX and Ctrl vs AX/GX comparisons (blue: upregulated in AX or AX/GX; orange: downregulated in AX or AX/GX).

(F) Schematic of the experimental design in the *Gata2*-cKO study[22] . Bar plot of differentially expressed genes across cell types (only these 5 cell types had enough cells for pseudobulk analysis). Graph showing inferred GATA2 regulated (activated: edges in purple; repressed: edges in orange) genes, including those related to hormone secretion (*Fshb*, *Chga*, *Pcsk1* - red) and a transcription factor (*Zfp618* - blue).

(G) Volcano plot of sex-biased gene expression in gonadotrophs in the entire CPA, colored by predicted activation or repression by GATA2. P-value is derived using Fisher's exact test.

(H) Schematic of how ESR1 and AR - downstream of sex hormones - lead to sex-biased gene expression directly or indirectly (e.g. through TFs like GATA2).

Figure S9., related to Figure 4

(A) Scatter plot of immune cell proportions across all RNA samples versus $\log_{10}$ age. The data suggest an increase in immune cells with age.

(B) Scatter plots of gene expression versus age (in $\log_{10}$ days) of 10 selected statistically significant changing signaling genes in stem cells.

(C) Scatter plots of gene expression versus age (in $\log_{10}$ days) of statistically significant changing TF genes in stem cells.

(D) Immunofluorescence staining against LEF1 (red) and SOX2 (green) in anterior pituitary parenchyma of mice at P3, P15 and P56. Scale bars 50 µm.

(E) Quantification of LEF1+SOX2+ double expressing cells over SOX2+ cells in the parenchyma (P, green) and marginal zone (MZ, blue) in P3, P15 and P56 animals. Each dot is a biological replicate. Two-sided paired T-test, *p*-values indicated.

Figure S10., related to Figure 4

(A) Scatter plots of 6 representative example genes with temporal patterns of each of the six categories: “decrease steadily”, “decrease-stagnate”, “stagnate-decrease”, “increase steadily”, “increase-stagnate”, “stagnate-increase”.

(B) Bar plot summarising age-dependent gene expression patterns in stem cells in the six temporal pattern categories.

(C) Gene set enrichment analysis of age-dependent gene expression hits in stem cells for the six temporal patterns. The “stagnate-decrease” pattern only had 7 genes and enrichment analysis was skipped.

Figure S11., related to Figure 5

(A) variancePartition box plot for RNA datasets showing the percentage variance explained by author, modality, sex and the residuals (everything else, including cell type specific biological signal).

(B) Dot plot of 20 genes (chosen at random) identified as stem cell markers in all three individual datasets examined in Figure 5B, but not using the CPA. Dot plot shows expression is not selective for stem cells.

(C) Line plot showing the same analysis as presented in Figure 5B, except repeated for all lineage comparisons, indicating the number of genes that fall within the various parts of the Venn diagram. The highest discrepancy is in the Thyrotroph vs Somatotroph/Lactotroph comparison comparisons, where 5097 genes are detected as significantly downregulated in the CPA, compared to 4 genes using the intersection of individual datasets.

(D) Dot plot of all Eph-Ephrin signaling genes.

(E) Dot plot of stem cell-specific Laminin signaling genes.

(F) Dot plot of BMP6 interaction genes, showing the possible signaling interaction between stem cells and other cells of the pituitary.

(G) Dot plot of FGF1-FGFR1/2 interaction genes, showing stem cells as the main *Fgf* source, in addition to pituicytes and corticotrophs. The main receptors *Fgfr1* and *Fgfr2* are highly expressed across cells, though *Fgfr1* is primarily specific to stem cells from anterior pituitary cell types.

(H) Dot plot of TGFB2-TGFBR interaction genes, showing stem cells as the main source of signals.

(I) Upset plot showing the intersection of cell type - differential expressed gene pairs across four high-fat diet studies. This analysis shows remarkably small overlap across the four studies.

(J) Bar plot of statistically significant upregulated (light blue) and downregulated (orange) genes across cell types in mice fed high-fat diets. These results are derived from joint linear mixed-modelling of all four studies.

(K) Bar plots of top 4 differentially expressed genes in stem cells (left) and immune cells (right), which includes *Cd8a* and *Cd8b1*.

Figure S12., related to Figure 6

(A) variancePartition box plot for ATAC datasets showing the percentage variance explained by author, modality, sex and the residuals (everything else, including cell type specific biological signal).

(B) Schematic of the number of RNA-only hit TFs in the differentiation hierarchy of the pituitary lineage. Purple numbers mark upregulated TFs, while orange numbers mark downregulated TFs at each branch point.

(C) Table of all RNA-only hits. Upregulated in purple, downregulated in orange.

(D) Box plot of *Nhlh2* expression, revealing female bias.

(E) Bar plot of TF motif fold-enrichment for each Multimodal hit TF in stem cells.

(F) Heatmap of the number of peaks marked by the motif of a stem cell Multimodal TF falling into various annotated regions. This plot offers an explanation as to why KLF motifs appear to be separated from the binding sites of other TFs. Specifically, KLF motifs appear enriched in proximal regulatory regions compared to other TFs.

(G) Strip plot of motif to TSS distances, with a threshold at 5kb. The X-axis is ordered according to the percentage of motif distances below 5kb. This further supports the hypothesis that KLF motifs are enriched in promoter proximal regions compared to other TF motifs.

Figure S1

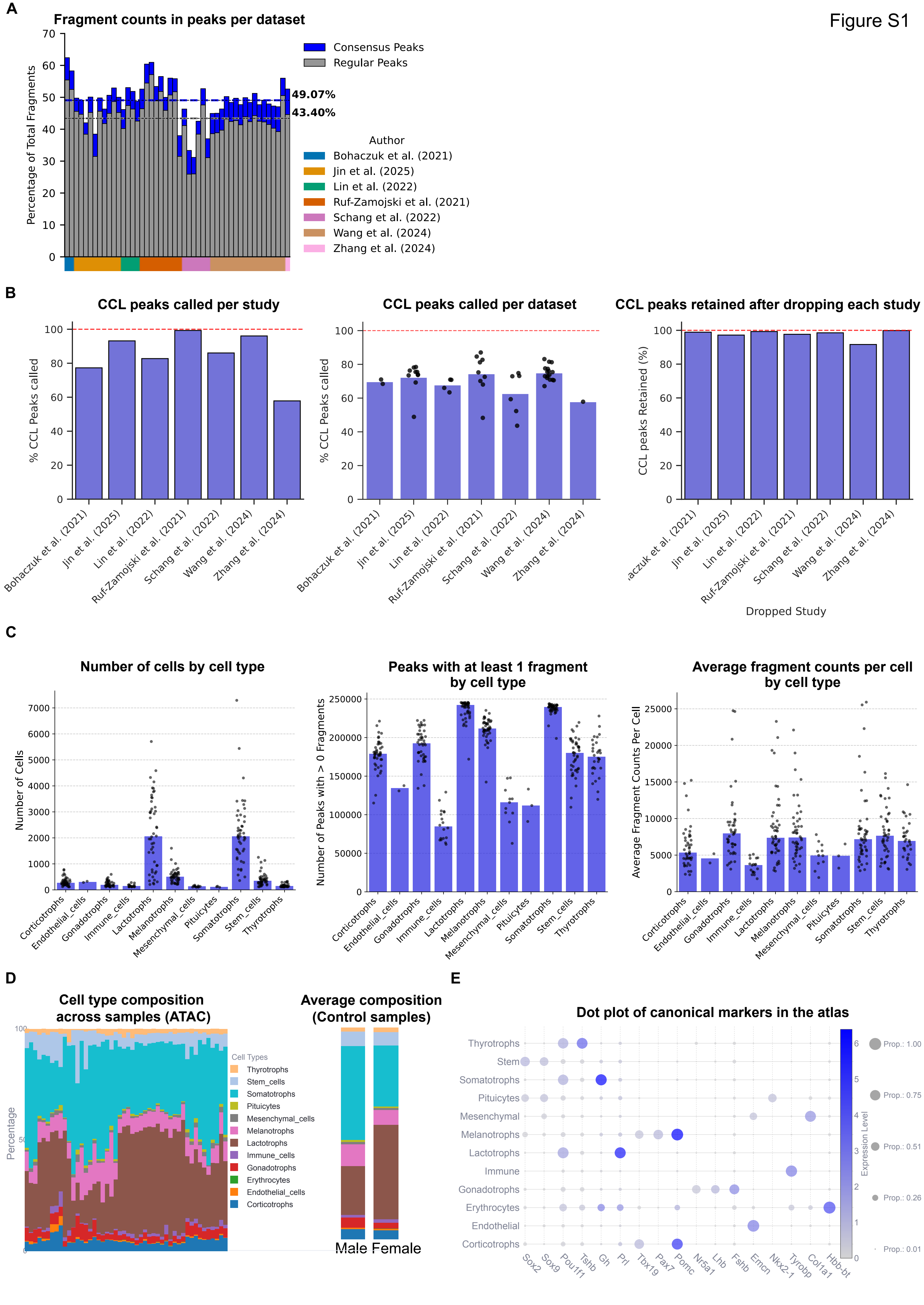
A
Fragment counts in peaks per dataset
Percentage of Total Fragments
Consensus Peaks
Regular Peaks
49.07%
43.40%
Author
Bohaczuk et al. (2021)
Jin et al. (2025)
Lin et al. (2022)
Ruf-Zamojski et al. (2021)
Schang et al. (2022)
Wang et al. (2024)
Zhang et al. (2024)
B
CCL peaks called per study
% CCL Peaks called
CCL peaks called per dataset
CCL peaks retained after dropping each study
CCL peaks Retained (%)
Dropped Study
C
Number of cells by cell type
Number of Cells
Peaks with at least 1 fragment by cell type
Number of Peaks with > 0 Fragments
Average fragment counts per cell by cell type
Average Fragment Counts Per Cell
Corticotrophs
Endothelial_cells
Gonadotrophs
Immune_cells
Lactotrophs
Melanotrophs
Mesenchymal_cells
Pituicytes
Somatotrophs
Stem_cells
Thyrotrophs
D
Cell type composition across samples (ATAC)
Percentage
Average composition (Control samples)
Cell Types
Erythrocytes
Male
Female
E
Dot plot of canonical markers in the atlas
Thyrotrophs
Stem
Somatotrophs
Pituicytes
Mesenchymal
Melanotrophs
Lactotrophs
Immune
Gonadotrophs
Erythrocytes
Endothelial
Corticotrophs
Sox2
Sox9
Pou1f1
Tshb
Gh
Prl
Tbx19
Pax7
Pomc
Nr5a1
Lhb
Fshb
Emcn
Nkx2-1
Tyrobp
Col1a1
Hbb-bt
Expression Level
Prop.: 1.00
Prop.: 0.75
Prop.: 0.51
Prop.: 0.26
Prop.: 0.01

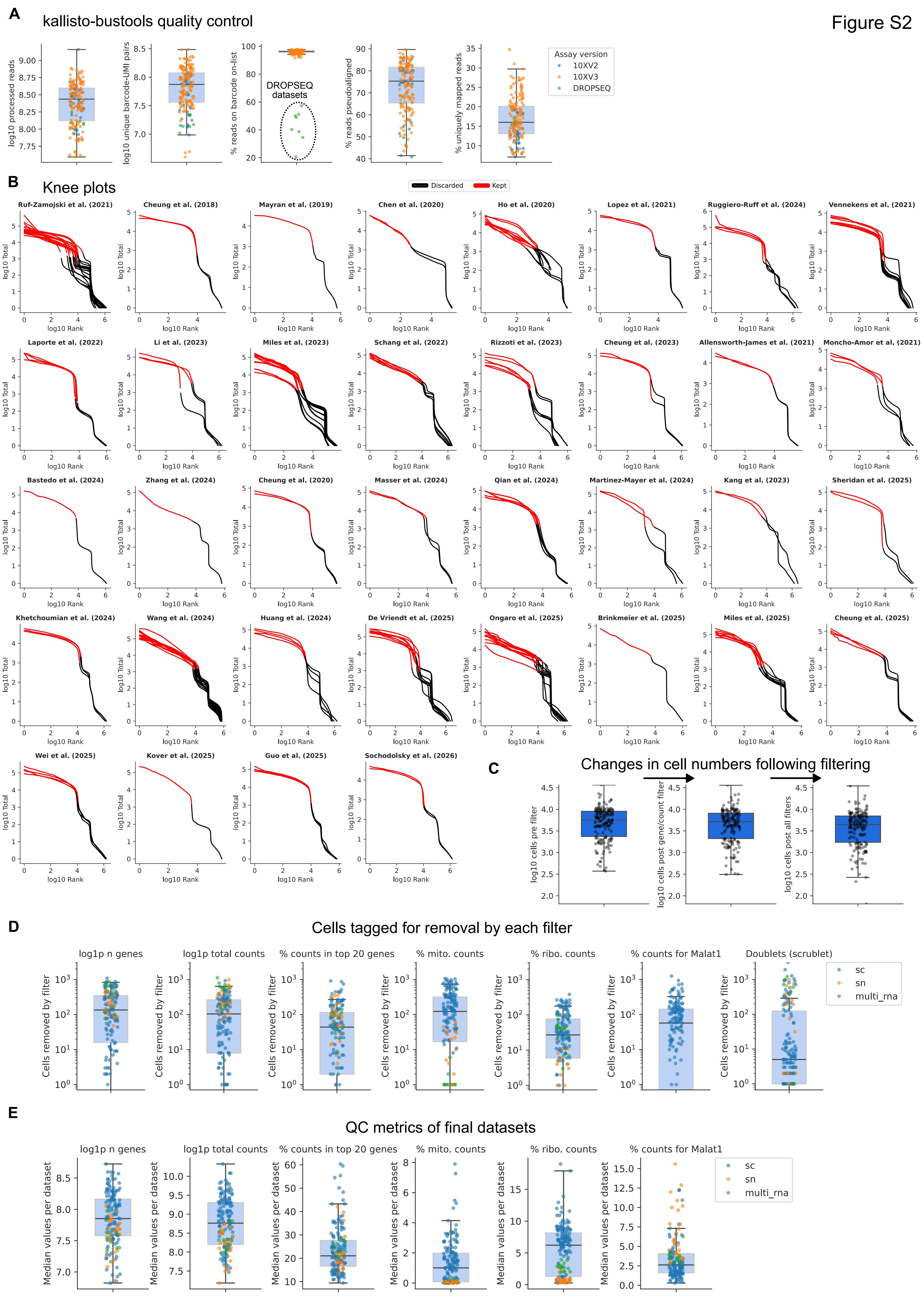
Figure S2
A
kallisto-bustools quality control
log10 processed reads
log10 unique barcode-UMI pairs
% reads on barcode on-list
DROPSEQ datasets
% reads pseudoaligned
% uniquely mapped reads
Assay version
10XV2
10XV3
DROPSEQ
B
Knee plots
Discarded
Kept
Ruf-Zamojski et al. (2021)
Cheung et al. (2018)
Mayran et al. (2019)
Chen et al. (2020)
Ho et al. (2020)
Lopez et al. (2021)
Ruggiero-Ruff et al. (2024)
Vennekens et al. (2021)
Laporte et al. (2022)
Li et al. (2023)
Miles et al. (2023)
Schang et al. (2022)
Rizzoti et al. (2023)
Cheung et al. (2023)
Allensworth-James et al. (2021)
Moncho-Amor et al. (2021)
Bastedo et al. (2024)
Zhang et al. (2024)
Cheung et al. (2020)
Masser et al. (2024)
Qian et al. (2024)
Martinez-Mayer et al. (2024)
Kang et al. (2023)
Sheridan et al. (2025)
Ketchoumian et al. (2024)
Wang et al. (2024)
Huang et al. (2024)
De Vriendt et al. (2025)
Ongaro et al. (2025)
Brinkmeier et al. (2025)
Miles et al. (2025)
Cheung et al. (2025)
Wei et al. (2025)
Kover et al. (2025)
Guo et al. (2025)
Sochodolsky et al. (2026)
log10 Total
log10 Rank
C
Changes in cell numbers following filtering
log10 cells pre filter
log10 cells post gene/count filter
log10 cells post all filters
D
Cells tagged for removal by each filter
log1p n genes
log1p total counts
% counts in top 20 genes
% mito. counts
% ribo. counts
% counts for Malat1
Doublets (scrublet)
Cells removed by filter
sc
sn
multi_rna
E
QC metrics of final datasets
log1p n genes
log1p total counts
% counts in top 20 genes
% mito. counts
% ribo. counts
% counts for Malat1
Median values per dataset
sc
sn
multi_rna

Figure S3

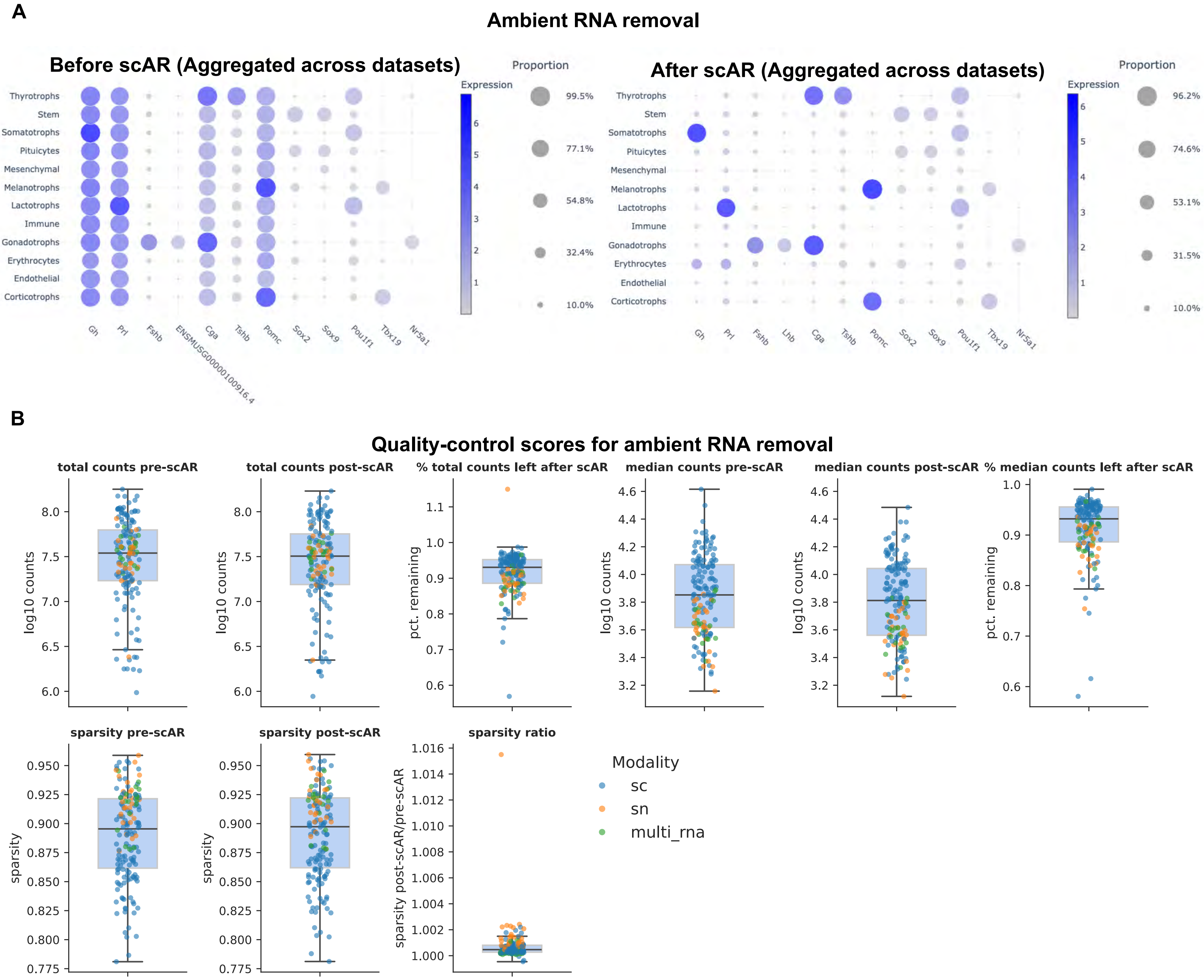
A
Ambient RNA removal
Before scAR (Aggregated across datasets)
After scAR (Aggregated across datasets)
Expression
Proportion
99.5%
77.1%
54.8%
32.4%
10.0%
96.2%
74.6%
53.1%
31.5%
Thyrotrophs
Stem
Somatotrophs
Pituicytes
Mesenchymal
Melanotrophs
Lactotrophs
Immune
Gonadotrophs
Erythrocytes
Endothelial
Corticotrophs
Gh
Prl
Fshb
ENSMUSG00000100916.4
Lhb
Cga
Tshb
Pomc
Sox2
Sox9
Pou1f1
Tbx19
Nr5a1
B
Quality-control scores for ambient RNA removal
total counts pre-scAR
total counts post-scAR
% total counts left after scAR
median counts pre-scAR
median counts post-scAR
% median counts left after scAR
log10 counts
pct. remaining
sparsity pre-scAR
sparsity post-scAR
sparsity ratio
sparsity
sparsity post-scAR/pre-scAR
Modality
sc
sn
multi_rna

Figure S4

A

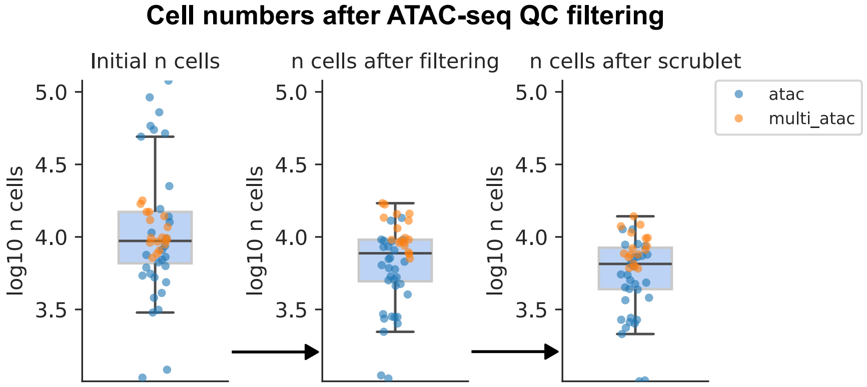
Cell numbers after ATAC-seq QC filtering
Initial n cells
n cells after filtering
n cells after scrublet
log10 n cells
5.0
4.5
4.0
3.5
atac
multi_atac

B

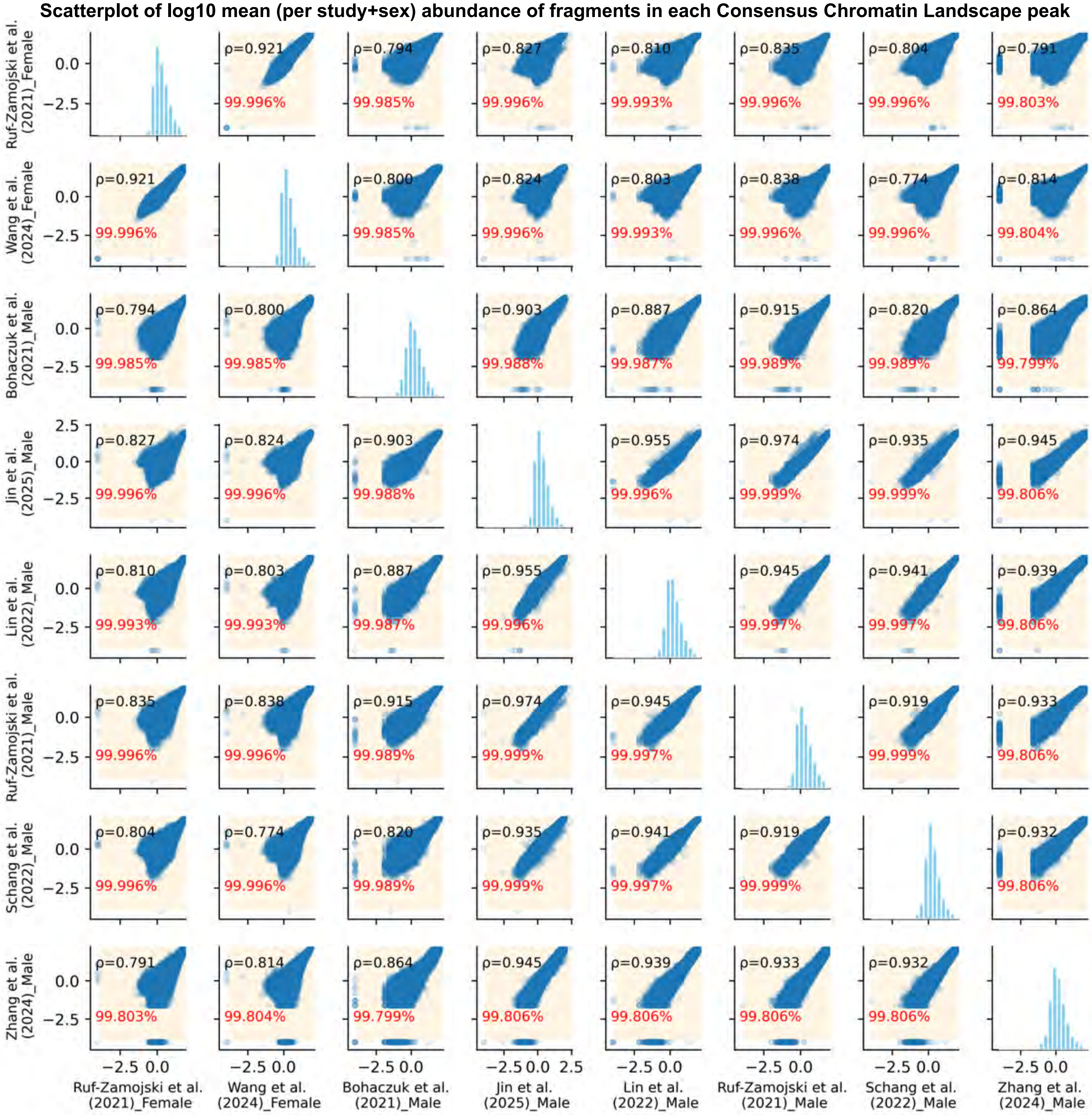
Scatterplot of log10 mean (per study+sex) abundance of fragments in each Consensus Chromatin Landscape peak
Ruf-Zamojski et al. (2021)_Female
ρ=0.921 99.996%
ρ=0.794 99.985%
ρ=0.827 99.996%
ρ=0.810 99.993%
ρ=0.835 99.996%
ρ=0.804 99.996%
ρ=0.791 99.803%
Wang et al. (2024)_Female
ρ=0.921 99.996%
ρ=0.800 99.985%
ρ=0.824 99.996%
ρ=0.803 99.993%
ρ=0.838 99.996%
ρ=0.774 99.996%
ρ=0.814 99.804%
Bohaczuk et al. (2021)_Male
ρ=0.794 99.985%
ρ=0.800 99.985%
ρ=0.903 99.988%
ρ=0.887 99.987%
ρ=0.915 99.989%
ρ=0.820 99.989%
ρ=0.864 99.799%
Jin et al. (2025)_Male
ρ=0.827 99.996%
ρ=0.824 99.996%
ρ=0.903 99.988%
ρ=0.955 99.996%
ρ=0.974 99.999%
ρ=0.935 99.999%
ρ=0.945 99.806%
Lin et al. (2022)_Male
ρ=0.810 99.993%
ρ=0.803 99.993%
ρ=0.887 99.987%
ρ=0.955 99.996%
ρ=0.945 99.997%
ρ=0.941 99.997%
ρ=0.939 99.806%
Ruf-Zamojski et al. (2021)_Male
ρ=0.835 99.996%
ρ=0.838 99.996%
ρ=0.915 99.989%
ρ=0.974 99.999%
ρ=0.945 99.997%
ρ=0.919 99.999%
ρ=0.933 99.806%
Schang et al. (2022)_Male
ρ=0.804 99.996%
ρ=0.774 99.996%
ρ=0.820 99.989%
ρ=0.935 99.999%
ρ=0.941 99.997%
ρ=0.919 99.999%
ρ=0.932 99.806%
Zhang et al. (2024)_Male
ρ=0.791 99.803%
ρ=0.814 99.804%
ρ=0.864 99.799%
ρ=0.945 99.806%
ρ=0.939 99.806%
ρ=0.933 99.806%
ρ=0.932 99.806%
2.5
0.0
−2.5
Ruf-Zamojski et al. (2021)_Female
Wang et al. (2024)_Female
Bohaczuk et al. (2021)_Male
Jin et al. (2025)_Male
Lin et al. (2022)_Male
Ruf-Zamojski et al. (2021)_Male
Schang et al. (2022)_Male
Zhang et al. (2024)_Male

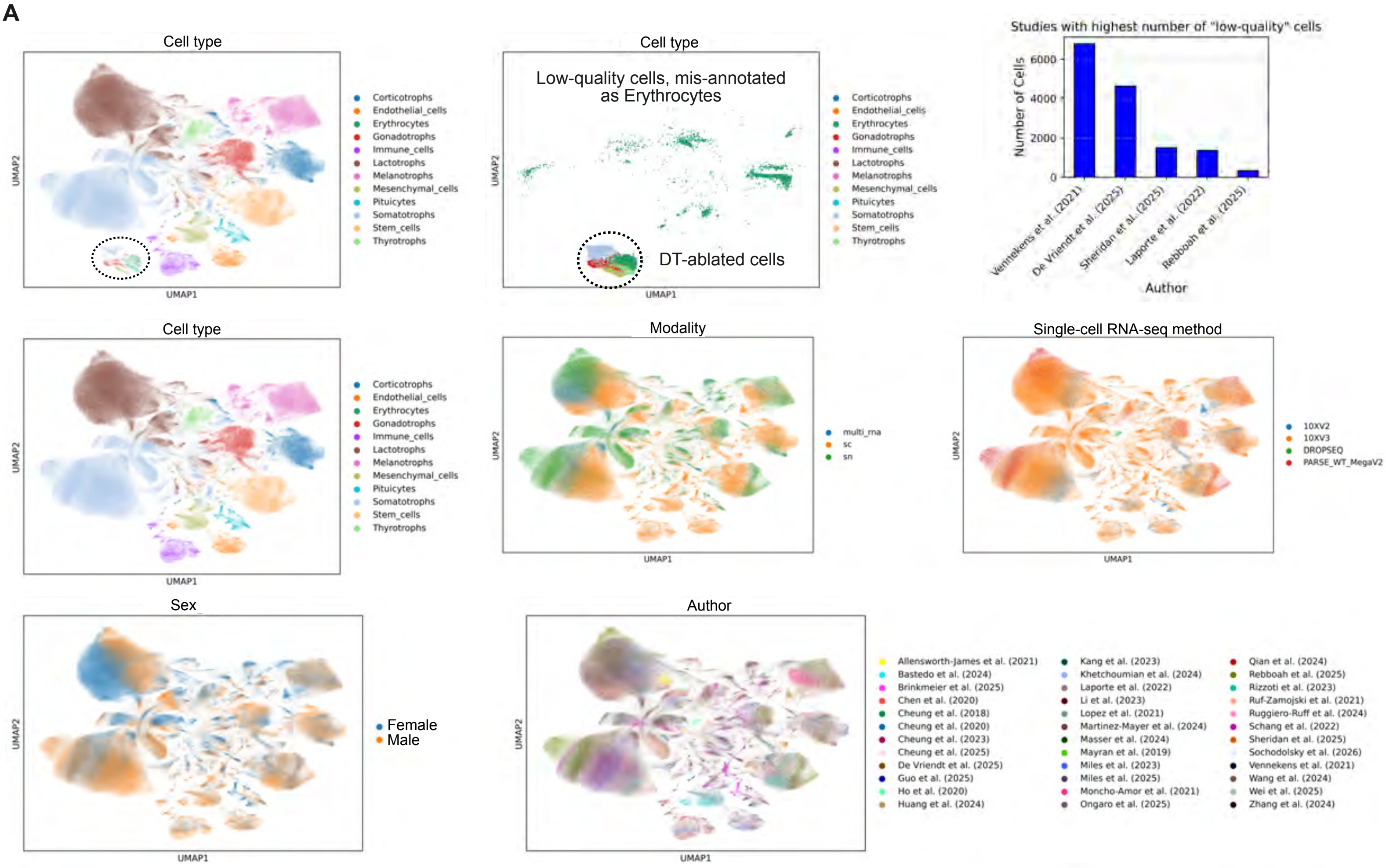

B

Method	Bio conservation: Isolated labels	KMeans NMI	KMeans ARI	Silhouette label	cLISI	Batch correction: Silhouette batch	iLISI	KBET	Graph connectivity	PCR comparison	Aggregate score: Batch correction	Bio conservation	Total
**X_scANVI**	0.53	0.74	0.58	0.68	1.00	0.79	0.02		0.87	0.33	0.50	0.71	0.62
**X_scVI**	0.48	0.67	0.48	0.57	1.00	0.85	0.02		0.86	0.34	0.52	0.64	0.59
**X_pca**	0.30	0.19	-0.07	0.29	1.00	0.62	0.01		0.83	0.00	0.36	0.34	0.35

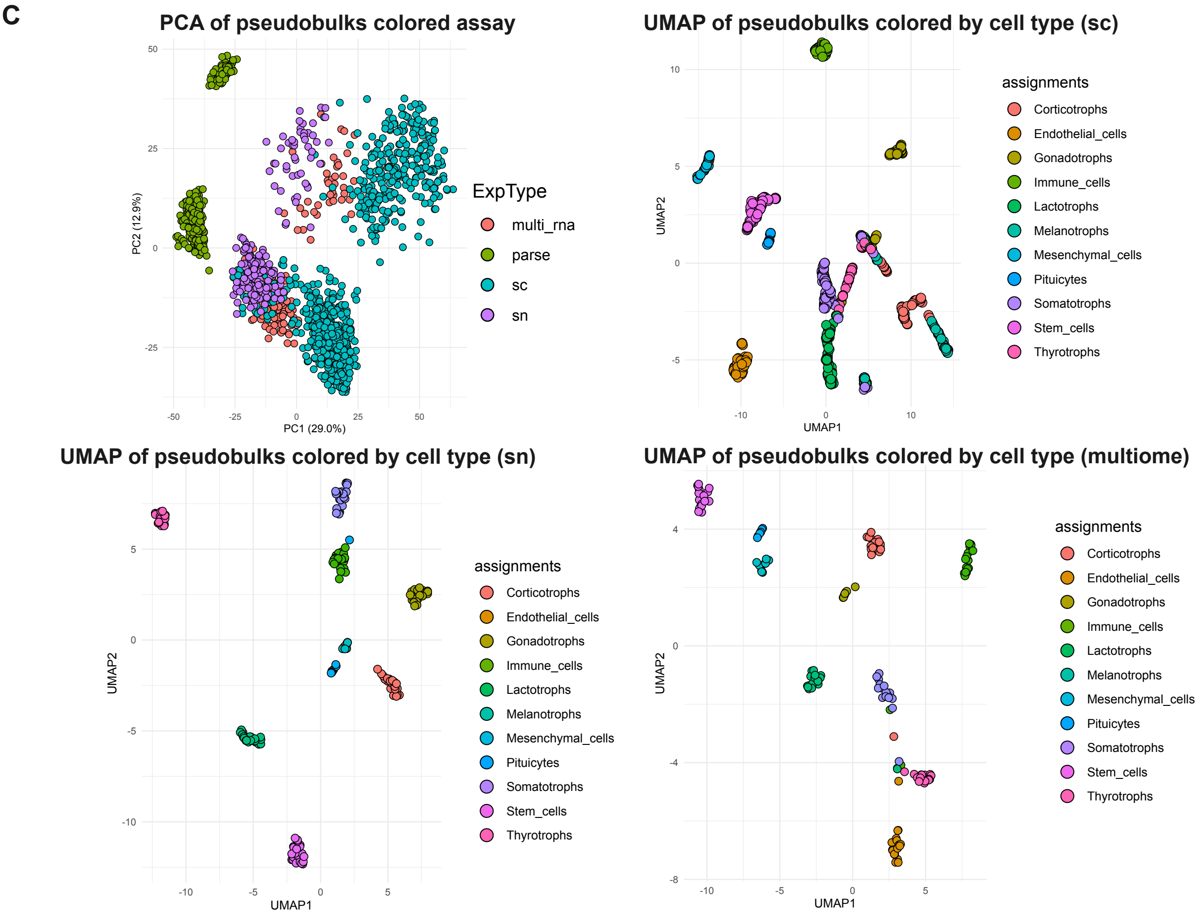

Figure S5

Figure S6

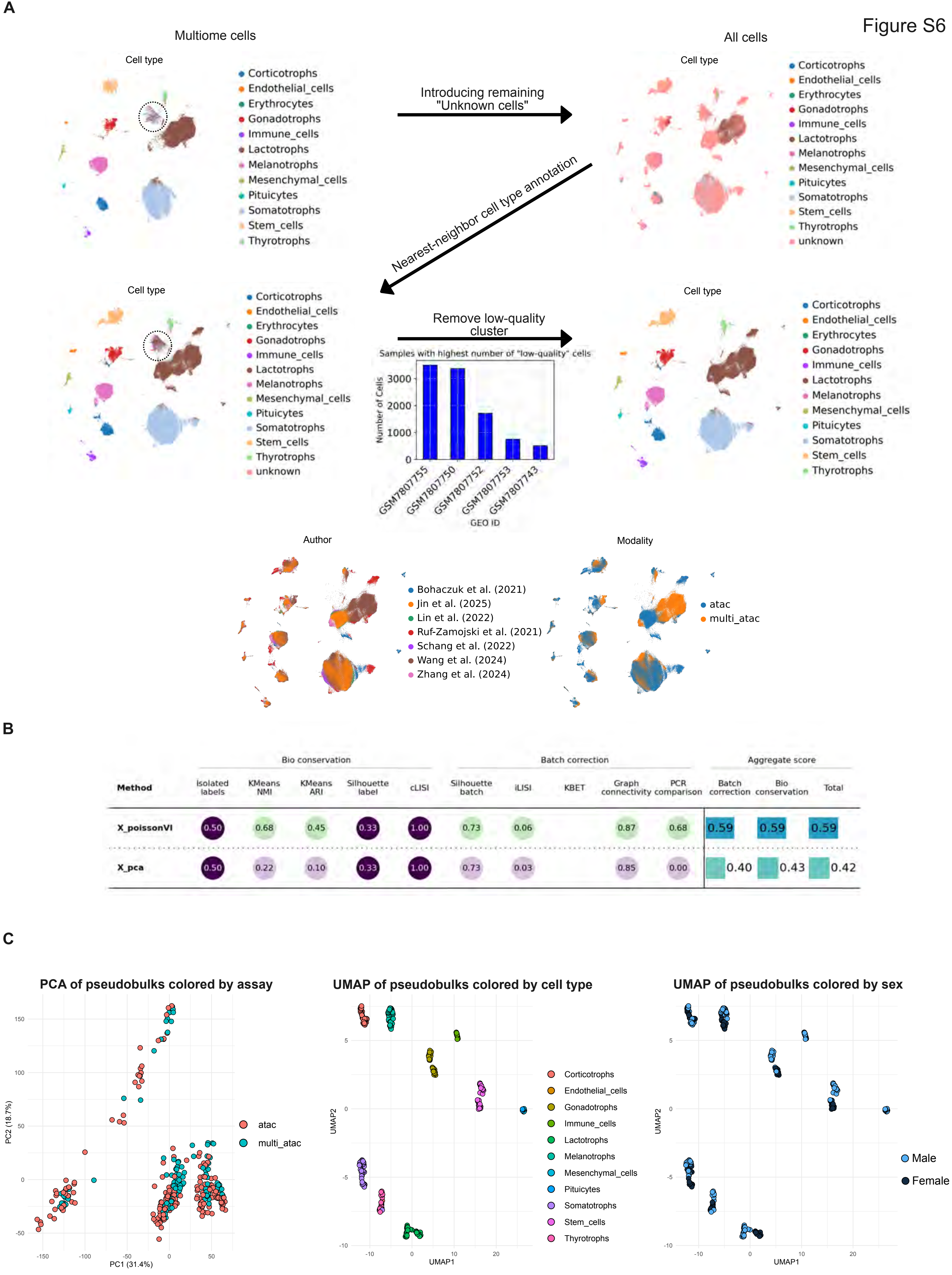
A
Multiome cells
All cells
Cell type
Corticotrophs
Endothelial_cells
Erythrocytes
Gonadotrophs
Immune_cells
Lactotrophs
Melanotrophs
Mesenchymal_cells
Pituicytes
Somatotrophs
Stem_cells
Thyrotrophs
unknown
Introducing remaining "Unknown cells"
Nearest-neighbor cell type annotation
Remove low-quality cluster
Samples with highest number of "low-quality" cells
Number of Cells
3000
2000
1000
0
GSM7807755
GSM7807750
GSM7807752
GSM7807753
GSM7807743
GEO ID
Author
Bohaczuk et al. (2021)
Jin et al. (2025)
Lin et al. (2022)
Ruf-Zamojski et al. (2021)
Schang et al. (2022)
Wang et al. (2024)
Zhang et al. (2024)
Modality
atac
multi_atac
B
Bio conservation
Batch correction
Aggregate score
Method
Isolated labels
KMeans NMI
KMeans ARI
Silhouette label
cLISI
Silhouette batch
iLISI
KBET
Graph connectivity
PCR comparison
Batch correction
Bio conservation
Total
X_poissonVI 0.50 0.68 0.45 0.33 1.00 0.73 0.06 0.87 0.68 0.59 0.59 0.59
X_pca 0.50 0.22 0.10 0.33 1.00 0.73 0.03 0.85 0.00 0.40 0.43 0.42
C
PCA of pseudobulks colored by assay
PC2 (18.7%)
PC1 (31.4%)
atac
multi_atac
UMAP of pseudobulks colored by cell type
UMAP1
UMAP2
Corticotrophs
Endothelial_cells
Gonadotrophs
Immune_cells
Lactotrophs
Melanotrophs
Mesenchymal_cells
Pituicytes
Somatotrophs
Stem_cells
Thyrotrophs
UMAP of pseudobulks colored by sex
Male
Female

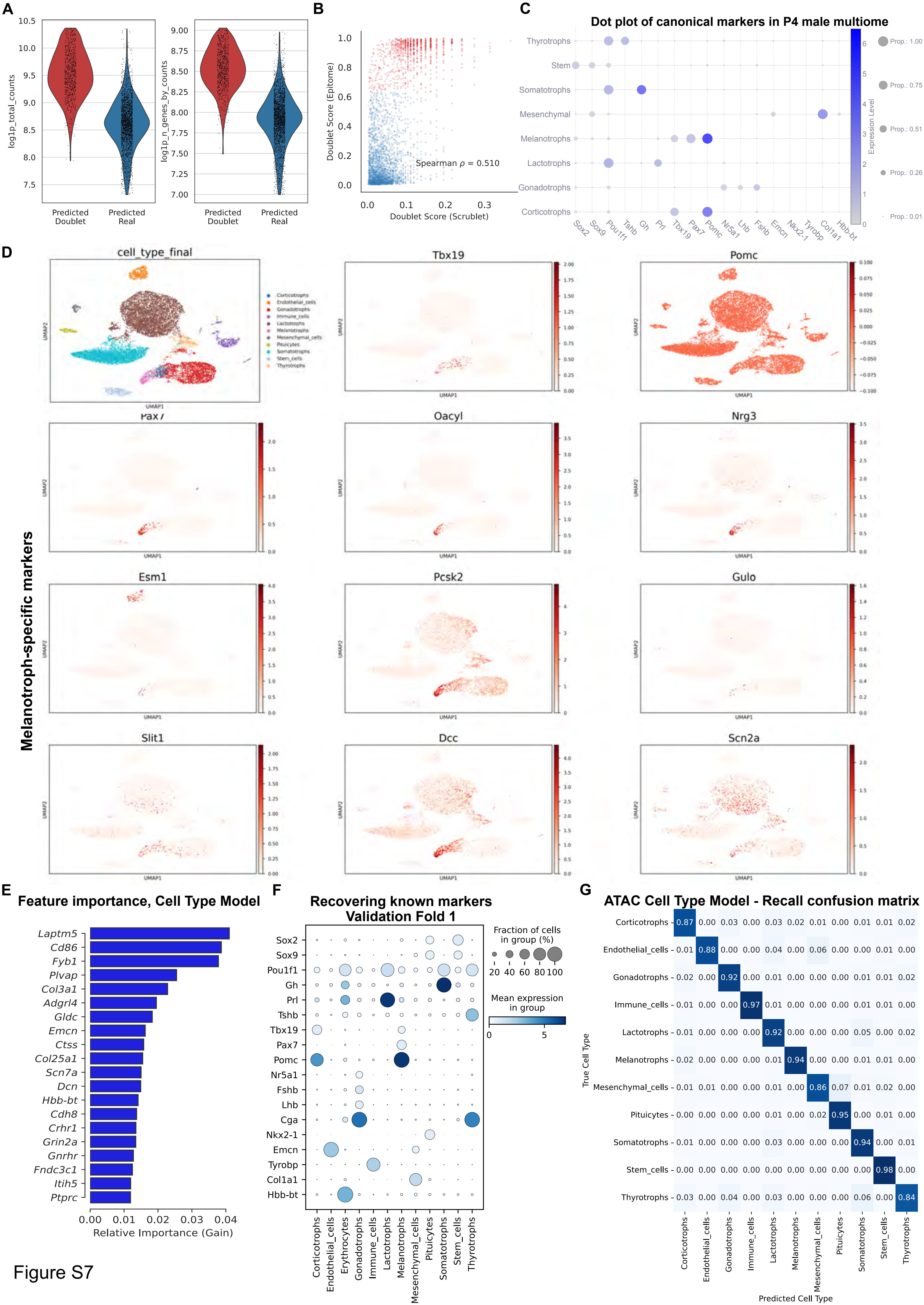
A
log1p_total_counts
log1p_n_genes_by_counts
Predicted Doublet
Predicted Real
B
Doublet Score (Epitome)
Doublet Score (Scrublet)
Spearman ρ = 0.510
C
Dot plot of canonical markers in P4 male multiome
Thyrotrophs
Stem
Somatotrophs
Mesenchymal
Melanotrophs
Lactotrophs
Gonadotrophs
Corticotrophs
Sox2 Sox9 Pou1f1 Tshb Gh Prl Tbx19 Pax7 Pomc Nr5a1 Lhb Fshb Emcn Nkx2-1 Tyrobp Col1a1 Hbb-bt
Expression Level
Prop.: 1.00
Prop.: 0.75
Prop.: 0.51
Prop.: 0.26
Prop.: 0.01
D
cell_type_final
Corticotrophs
Endothelial_cells
Gonadotrophs
Immune_cells
Lactotrophs
Melanotrophs
Mesenchymal_cells
Pituicytes
Somatotrophs
Stem_cells
Thyrotrophs
Tbx19
Pomc
Pax7
Oacyl
Nrg3
Esm1
Pcsk2
Gulo
Slit1
Dcc
Scn2a
UMAP1
UMAP2
Melanotroph-specific markers
E
Feature importance, Cell Type Model
Laptm5
Cd86
Fyb1
Plvap
Col3a1
Adgrl4
Gldc
Emcn
Ctss
Col25a1
Scn7a
Dcn
Hbb-bt
Cdh8
Crhr1
Grin2a
Gnrhr
Fndc3c1
Itih5
Ptprc
Relative Importance (Gain)
F
Recovering known markers
Validation Fold 1
Sox2
Sox9
Pou1f1
Gh
Prl
Tshb
Tbx19
Pax7
Pomc
Nr5a1
Fshb
Lhb
Cga
Nkx2-1
Emcn
Tyrobp
Col1a1
Hbb-bt
Corticotrophs
Endothelial_cells
Erythrocytes
Gonadotrophs
Immune_cells
Lactotrophs
Melanotrophs
Mesenchymal_cells
Pituicytes
Somatotrophs
Stem_cells
Thyrotrophs
Fraction of cells in group (%)
20 40 60 80 100
Mean expression in group
G
ATAC Cell Type Model - Recall confusion matrix
Corticotrophs - 0.87 0.00 0.03 0.00 0.03 0.02 0.01 0.00 0.01 0.01 0.02
Endothelial_cells - 0.01 0.88 0.00 0.00 0.04 0.00 0.06 0.00 0.00 0.01 0.00
Gonadotrophs - 0.02 0.00 0.92 0.00 0.01 0.00 0.00 0.00 0.01 0.01 0.02
Immune_cells - 0.01 0.00 0.00 0.97 0.01 0.00 0.00 0.00 0.01 0.01 0.00
Lactotrophs - 0.01 0.00 0.00 0.00 0.92 0.00 0.00 0.00 0.05 0.00 0.02
Melanotrophs - 0.02 0.00 0.00 0.00 0.01 0.94 0.00 0.00 0.01 0.01 0.00
Mesenchymal_cells - 0.01 0.01 0.00 0.00 0.01 0.00 0.86 0.07 0.01 0.02 0.00
Pituicytes - 0.00 0.00 0.00 0.00 0.01 0.00 0.02 0.95 0.00 0.01 0.00
Somatotrophs - 0.01 0.00 0.00 0.00 0.03 0.00 0.00 0.00 0.94 0.00 0.01
Stem_cells - 0.00 0.00 0.00 0.00 0.00 0.00 0.00 0.00 0.00 0.98 0.00
Thyrotrophs - 0.03 0.00 0.04 0.00 0.03 0.00 0.00 0.00 0.06 0.00 0.84
True Cell Type
Predicted Cell Type
Corticotrophs
Endothelial_cells
Gonadotrophs
Immune_cells
Lactotrophs
Melanotrophs
Mesenchymal_cells
Pituicytes
Somatotrophs
Stem_cells
Thyrotrophs

Figure S7

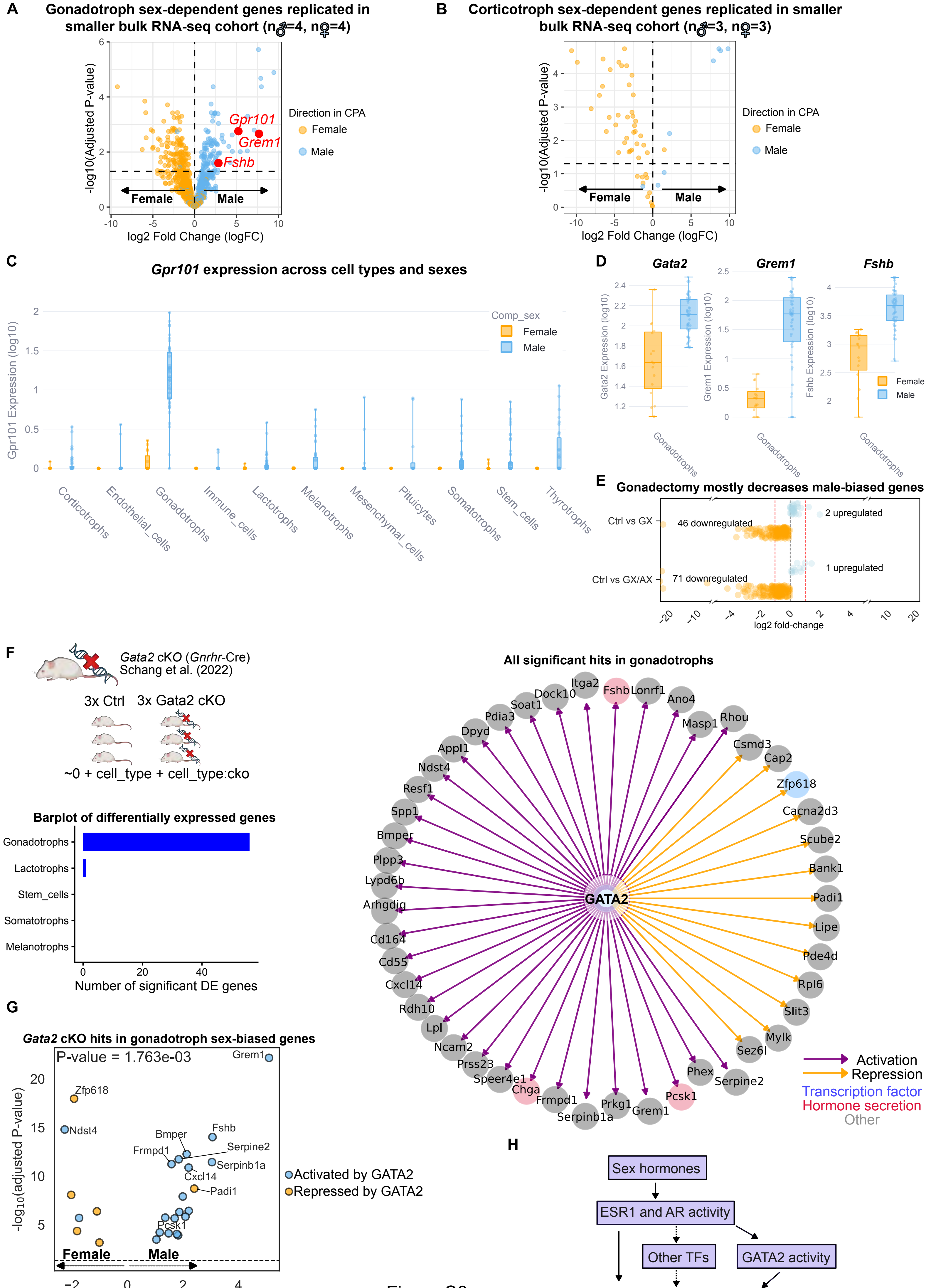
A
Gonadotroph sex-dependent genes replicated in smaller bulk RNA-seq cohort (n♂=4, n♀=4)
-log10(Adjusted P-value)
log2 Fold Change (logFC)
Gpr101
Grem1
Fshb
Female
Male
Direction in CPA
Female
Male
B
Corticotroph sex-dependent genes replicated in smaller bulk RNA-seq cohort (n♂=3, n♀=3)
-log10(Adjusted P-value)
log2 Fold Change (logFC)
Female
Male
Direction in CPA
Female
Male
C
Gpr101 expression across cell types and sexes
Gpr101 Expression (log10)
Comp_sex
Female
Male
Corticotrophs
Endothelial_cells
Gonadotrophs
Immune_cells
Lactotrophs
Melanotrophs
Mesenchymal_cells
Pituicytes
Somatotrophs
Stem_cells
Thyrotrophs
D
Gata2
Grem1
Fshb
Gata2 Expression (log10)
Grem1 Expression (log10)
Fshb Expression (log10)
Gonadotrophs
Female
Male
E
Gonadectomy mostly decreases male-biased genes
Ctrl vs GX
Ctrl vs GX/AX
46 downregulated
2 upregulated
71 downregulated
1 upregulated
log2 fold-change
F
Gata2 cKO (Gnrhr-Cre)
Schang et al. (2022)
3x Ctrl
3x Gata2 cKO
~0 + cell_type + cell_type:cko
Barplot of differentially expressed genes
Gonadotrophs
Lactotrophs
Stem_cells
Somatotrophs
Melanotrophs
Number of significant DE genes
All significant hits in gonadotrophs
GATA2
Itga2
Fshb
Lonrf1
Ano4
Dock10
Soat1
Pdia3
Dpyd
Appl1
Ndst4
Resf1
Spp1
Bmper
Plpp3
Lypd6b
Arhgdig
Cd164
Cd55
Cxcl14
Rdh10
Lpl
Ncam2
Prss23
Speer4e1
Chga
Frmpd1
Serpinb1a
Prkg1
Grem1
Pcsk1
Phex
Serpine2
Masp1
Rhou
Csmd3
Cap2
Zfp618
Cacna2d3
Scube2
Bank1
Padi1
Lipe
Pde4d
Rpl6
Slit3
Mylk
Sez6l
Activation
Repression
Transcription factor
Hormone secretion
Other
G
Gata2 cKO hits in gonadotroph sex-biased genes
P-value = 1.763e-03
-log10(adjusted P-value)
log2 Fold Change
Grem1
Zfp618
Ndst4
Bmper
Fshb
Frmpd1
Serpine2
Serpinb1a
Cxcl14
Padi1
Pcsk1
Female
Male
Activated by GATA2
Repressed by GATA2
H
Sex hormones
ESR1 and AR activity
Other TFs
GATA2 activity
Sex-biased gene regulation

Figure S8

Figure S9

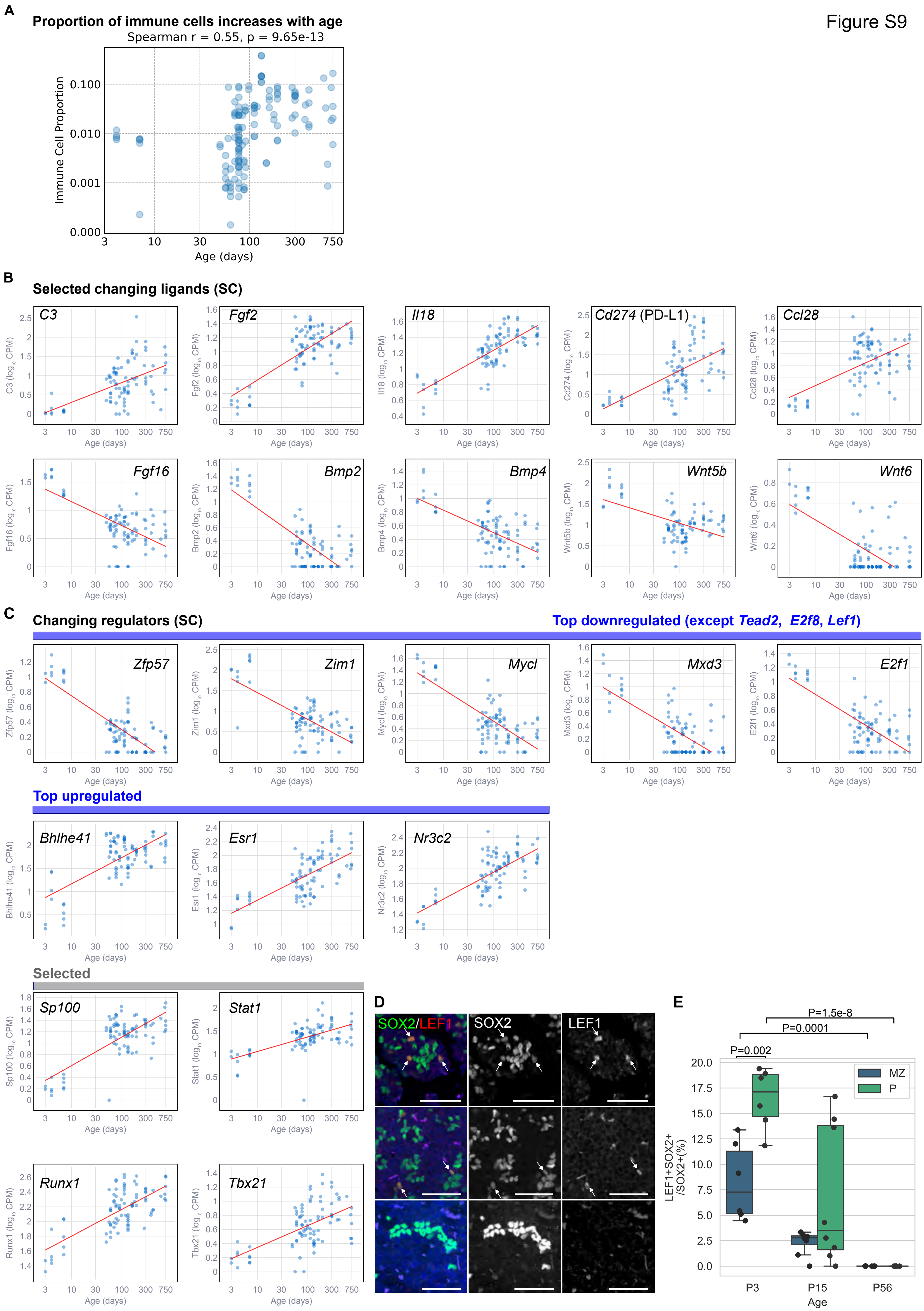
A
Proportion of immune cells increases with age
Spearman r = 0.55, p = 9.65e-13
Immune Cell Proportion
0.100
0.010
0.001
0.000
3
10
30
100
300
750
Age (days)
B
Selected changing ligands (SC)
C3
Fgf2
Il18
Cd274 (PD-L1)
Ccl28
Fgf16
Bmp2
Bmp4
Wnt5b
Wnt6
C
Changing regulators (SC)
Top downregulated (except Tead2, E2f8, Lef1)
Zfp57
Zim1
Mycl
Mxd3
E2f1
Top upregulated
Bhlhe41
Esr1
Nr3c2
Selected
Sp100
Stat1
Runx1
Tbx21
D
SOX2/LEF1
SOX2
LEF1
E
P=1.5e-8
P=0.0001
P=0.002
MZ
P
LEF1+SOX2+ /SOX2+(%)
P3
P15
P56
Age

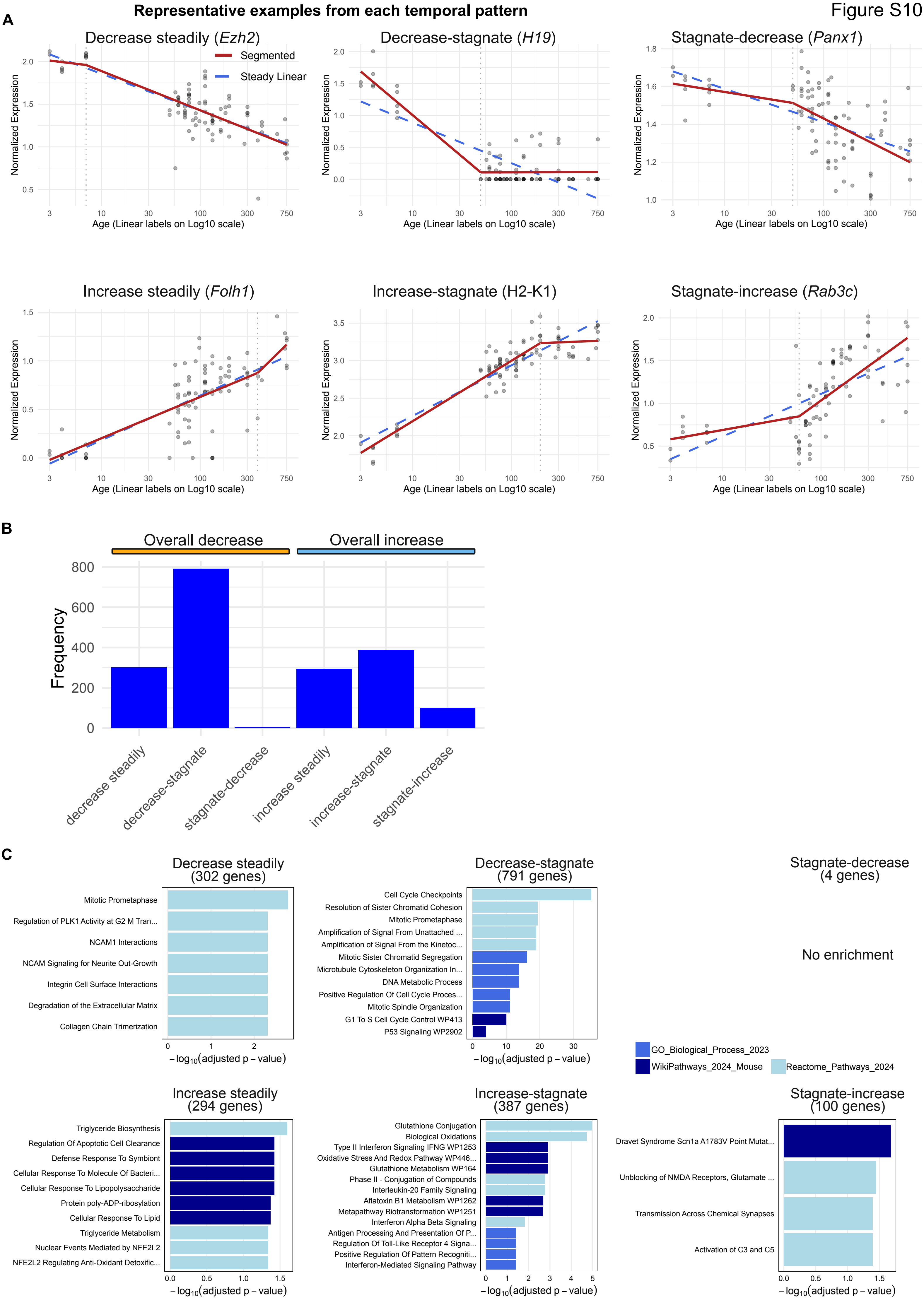
Representative examples from each temporal pattern
A
Decrease steadily (Ezh2)
Segmented
Steady Linear
Decrease-stagnate (H19)
Stagnate-decrease (Panx1)
Increase steadily (Folh1)
Increase-stagnate (H2-K1)
Stagnate-increase (Rab3c)
Normalized Expression
Age (Linear labels on Log10 scale)
B
Overall decrease
Overall increase
Frequency
decrease steadily
decrease-stagnate
stagnate-decrease
increase steadily
increase-stagnate
stagnate-increase
C
Decrease steadily (302 genes)
Mitotic Prometaphase
Regulation of PLK1 Activity at G2 M Tran...
NCAM1 Interactions
NCAM Signaling for Neurite Out-Growth
Integrin Cell Surface Interactions
Degradation of the Extracellular Matrix
Collagen Chain Trimerization
Decrease-stagnate (791 genes)
Cell Cycle Checkpoints
Resolution of Sister Chromatid Cohesion
Mitotic Prometaphase
Amplification of Signal From Unattached ...
Amplification of Signal From the Kinetoc...
Mitotic Sister Chromatid Segregation
Microtubule Cytoskeleton Organization In...
DNA Metabolic Process
Positive Regulation Of Cell Cycle Proces...
Mitotic Spindle Organization
G1 To S Cell Cycle Control WP413
P53 Signaling WP2902
Stagnate-decrease (4 genes)
No enrichment
GO_Biological_Process_2023
WikiPathways_2024_Mouse
Reactome_Pathways_2024
Increase steadily (294 genes)
Triglyceride Biosynthesis
Regulation Of Apoptotic Cell Clearance
Defense Response To Symbiont
Cellular Response To Molecule Of Bacteri...
Cellular Response To Lipopolysaccharide
Protein poly-ADP-ribosylation
Cellular Response To Lipid
Triglyceride Metabolism
Nuclear Events Mediated by NFE2L2
NFE2L2 Regulating Anti-Oxidant Detoxific...
Increase-stagnate (387 genes)
Glutathione Conjugation
Biological Oxidations
Type II Interferon Signaling IFNG WP1253
Oxidative Stress And Redox Pathway WP446...
Glutathione Metabolism WP164
Phase II - Conjugation of Compounds
Interleukin-20 Family Signaling
Aflatoxin B1 Metabolism WP1262
Metapathway Biotransformation WP1251
Interferon Alpha Beta Signaling
Antigen Processing And Presentation Of P...
Regulation Of Toll-Like Receptor 4 Signa...
Positive Regulation Of Pattern Recogniti...
Interferon-Mediated Signaling Pathway
Stagnate-increase (100 genes)
Dravet Syndrome Scn1a A1783V Point Mutat...
Unblocking of NMDA Receptors, Glutamate ...
Transmission Across Chemical Synapses
Activation of C3 and C5
−log10(adjusted p − value)

Figure S11

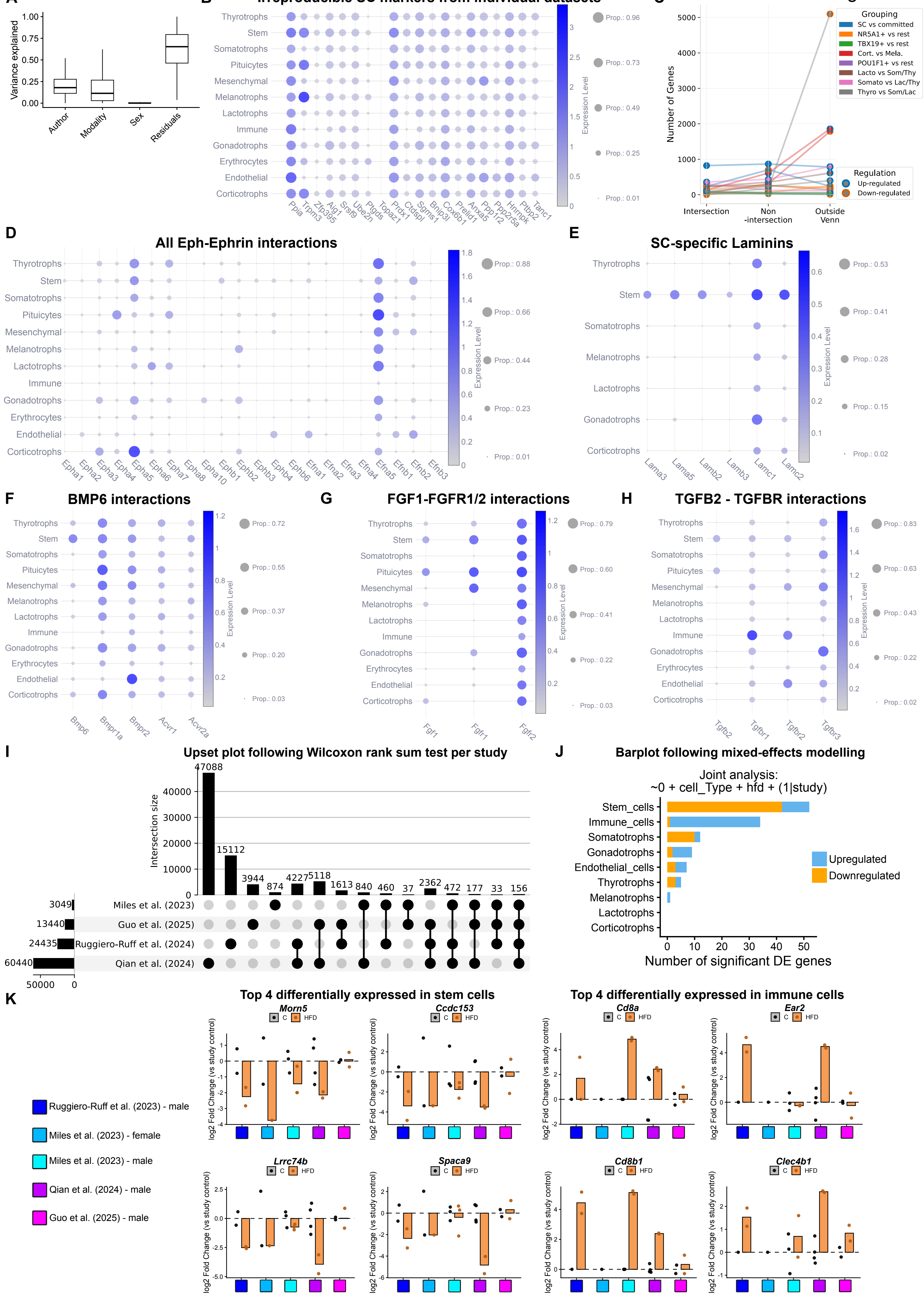
A
Variance explained
Author
Modality
Sex
Residuals
B
Irreproducible SC markers from individual datasets
Thyrotrophs
Stem
Somatotrophs
Pituicytes
Mesenchymal
Melanotrophs
Lactotrophs
Immune
Gonadotrophs
Erythrocytes
Endothelial
Corticotrophs
Ppia
Trpm3
Zfp395
Alg1
Srsf9
Ube2n
Ptgds
Topaz1
Prdx1
Ctdspl
Sgms1
Bnip3l
Cox6b1
Prelid1
Anxa5
Ppp1r2
Ppp2r5a
Hnrnpk
Ptbp2
Tanc1
Expression Level
Prop.: 0.96
Prop.: 0.73
Prop.: 0.49
Prop.: 0.25
Prop.: 0.01
C
Number of Genes
Intersection
Non -intersection
Outside Venn
Grouping
SC vs committed
NR5A1+ vs rest
TBX19+ vs rest
Cort. vs Mela.
POU1F1+ vs rest
Lacto vs Som/Thy
Somato vs Lac/Thy
Thyro vs Som/Lac
Regulation
Up-regulated
Down-regulated
D
All Eph-Ephrin interactions
Epha1
Epha2
Epha3
Epha4
Epha5
Epha6
Epha7
Epha8
Epha10
Ephb1
Ephb2
Ephb3
Ephb4
Ephb6
Efna1
Efna2
Efna3
Efna4
Efna5
Efnb1
Efnb2
Efnb3
Prop.: 0.88
Prop.: 0.66
Prop.: 0.44
Prop.: 0.23
Prop.: 0.01
E
SC-specific Laminins
Lama3
Lama5
Lamb2
Lamb3
Lamc1
Lamc2
Prop.: 0.53
Prop.: 0.41
Prop.: 0.28
Prop.: 0.15
Prop.: 0.02
F
BMP6 interactions
Bmp6
Bmpr1a
Bmpr2
Acvr1
Acvr2a
Prop.: 0.72
Prop.: 0.55
Prop.: 0.37
Prop.: 0.20
Prop.: 0.03
G
FGF1-FGFR1/2 interactions
Fgf1
Fgfr1
Fgfr2
Prop.: 0.79
Prop.: 0.60
Prop.: 0.41
Prop.: 0.22
Prop.: 0.03
H
TGFB2 - TGFBR interactions
Tgfb2
Tgfbr1
Tgfbr2
Tgfbr3
Prop.: 0.83
Prop.: 0.63
Prop.: 0.43
Prop.: 0.22
Prop.: 0.02
I
Upset plot following Wilcoxon rank sum test per study
Intersection size
47088
15112
3944
874
4227
5118
1613
840
460
37
2362
472
177
33
156
3049
13440
24435
60440
Miles et al. (2023)
Guo et al. (2025)
Ruggiero-Ruff et al. (2024)
Qian et al. (2024)
J
Barplot following mixed-effects modelling
Joint analysis:
~0 + cell_Type + hfd + (1|study)
Stem_cells
Immune_cells
Somatotrophs
Gonadotrophs
Endothelial_cells
Thyrotrophs
Melanotrophs
Lactotrophs
Corticotrophs
Upregulated
Downregulated
Number of significant DE genes
K
Top 4 differentially expressed in stem cells
Top 4 differentially expressed in immune cells
Morn5
Ccdc153
Cd8a
Ear2
Lrrc74b
Spaca9
Cd8b1
Clec4b1
C
HFD
log2 Fold Change (vs study control)
Ruggiero-Ruff et al. (2023) - male
Miles et al. (2023) - female
Miles et al. (2023) - male
Qian et al. (2024) - male
Guo et al. (2025) - male

Figure S12

A

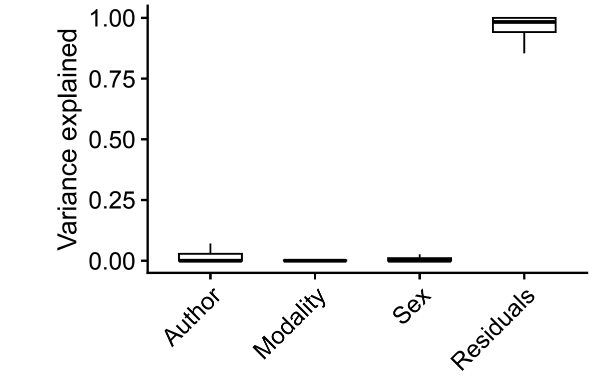

B

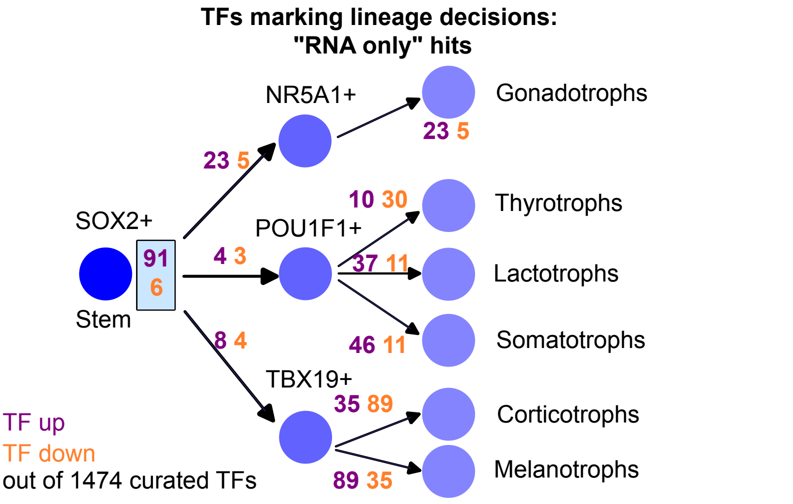

C

Table of "RNA only" hits

Stem cells vs committed cells
Aebp2, Ahr, Atf3, Bach2, Bnc2, Carhsp1, Cebpb, Creb3l4, Creb5, Dbp, Dmrta1, Dmrta2, E2f5, Egr1, Ets1, Ets2, Etv4, Fosl1, Foxc1, Foxj1, Foxm1, Glis2, Hes1, Hey1, Hey2, Heyl, Hmga2, Hmgb1, Id1, Id2, Id3, Id4, Ikzf2, Irf6, Isl2, Jdp2, Jun, Jund, Lhx3, Lmx1b, Mafb, Maff, Mafk, Mecom, Mef2b, Meis2, Msx1, Myc, Nfatc1, Nfatc4, Nfe2l3, Nr1d1, Nr1h2, Nr2f2, Nr2f6, Nr4a3, Otx1, Ovol2, Pax6, Ppara, Prdm16, Prrx1, Prrx2, Rarb, Rarg, Sall1, Sall2, Smad7, Sp100, Tcf7l1, Tef, Terf1, Tfeb, Tgif1, Tgif2, Trp63, Tsc22d2, Tsc22d3, Zeb2, Zfp217, Zfp219, Zfp422, Zfp467, Zfp599, Zfp7, Zfp707, Zfp710, Zfp711, Zfp768, Zfp870, Zfp993, Arnt2, Insm1, Mzf1, Plag1, Plagl1, Prox1

NR5A1+ lineage vs other pituitary cell types
Aff2, Aff3, Foxp2, Glis1, Hes6, Irf8, Lhx4, Nr0b1, Nr0b2, Pbx4, Pgr, Sox13, Sox5, Sp140, St18, Tcf24, Tcf7, Zfp41, Zfp579, Zfp661, Zfp820, Zfp872, Zfpm1, E2f7, Ets1, Etv5, Gli2, Zbtb7c

TBX19+ lineage vs other pituitary cell types
Egr4, Esrrb, Hopx, Nr4a2, Prdm1, Scx, Zfp474, Zim3, Lef1, Meis2, Pitx2, Thrb

Melanotrophs vs Corticotrophs
Table S12

POU1F1+ lineage vs other pituitary cell types
Klf14, Rxrg, Shox2, Vdr, Klf16, Npas2, Zbtb18

Lactotrophs vs Somatotrophs, Thyrotrophs
Table S12

Somatotrophs vs Lactotrophs, Thyrotrophs
Table S12

Thyrotrophs vs Somatotrophs, Lactotrophs
Table S12

D

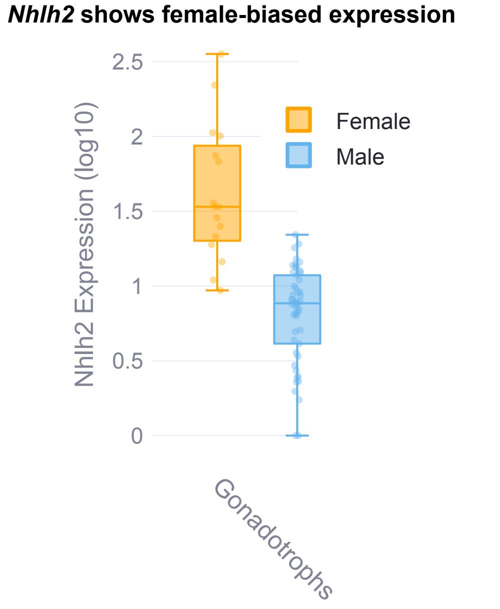

E

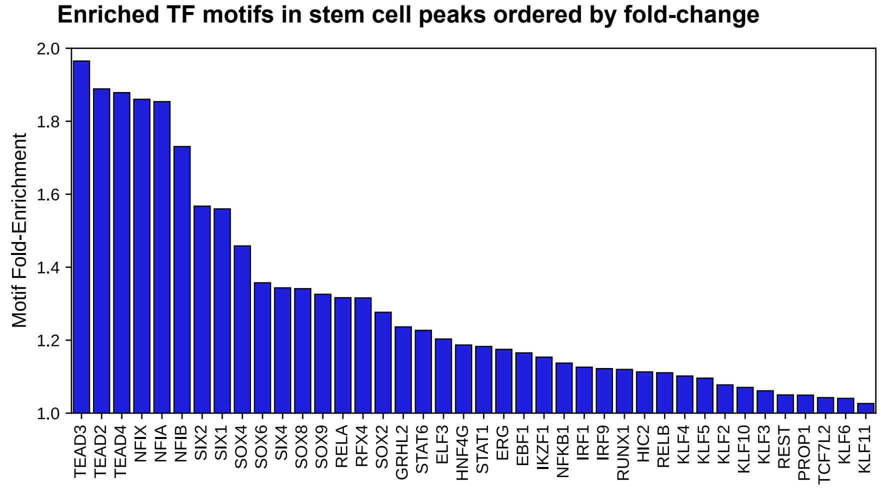

F

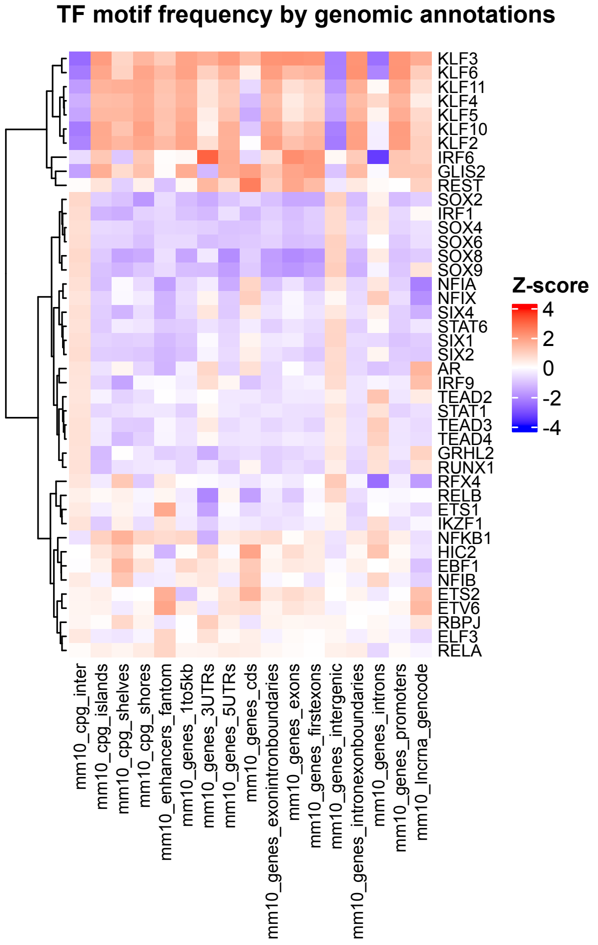

G

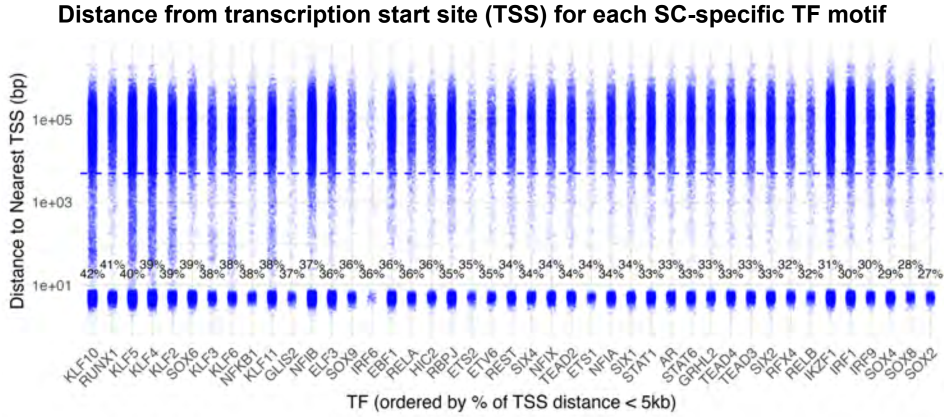

Supplement: 1 [file NIHMS2190707-supplement-1.pdf]
